# Antiviral activity of newly synthesized pyrazole derivatives against Newcastle disease virus

**DOI:** 10.1038/s41598-025-03495-6

**Published:** 2025-05-28

**Authors:** Ahmed El-Sewedy, Alaa R. I. Morsy, Eman A. El-Bordany, Naglaa F. H. Mahmoud, Safwa Z. Mohamed, Sayed K. Ramadan

**Affiliations:** 1 Chemistry Department, Faculty of Science, Ain Shams University, Cairo, 11566 Egypt; 2 Central Laboratory for Evaluation of Veterinary Biologics (CLEVB), Agricultural Research Center, Cairo, Egypt

**Keywords:** Antiviral, 5-Chloropyrazole, Newcastle disease virus, Molecular Docking, Modeling pharmacokinetics, Biological techniques, Drug discovery, Diseases, Chemistry

## Abstract

Newcastle disease virus (NDV) is a highly contagious poultry disease that affects the respiratory, nervous, and digestive systems, causing significant losses to the poultry industry. Pyrazole-based scaffolds had significant potential as antiviral agents targeting various pathogens. Thus, a series of 4-substituted pyrazole derivatives were synthesized by reacting 5-chloro-4-formyl-3-methyl-1-phenylpyrazole with some nitrogen and carbon-based nucleophiles. The antiviral efficacy of these compounds was evaluated against NDV by assessing their ability to inhibit virus-induced haemagglutination. Notably, hydrazone **6** and thiazolidinedione derivative **9** achieved complete (100%) protection against NDV with 0% mortality, while the pyrazolopyrimidine derivative **7** provided 95% protection. Additionally, tetrazine **4** and chalcone **11** conferred 85% and 80% protection, respectively. Molecular docking simulation targeting immune receptor TLR4 protein (PDB ID: 3MU3) revealed that compound **6** achieved the highest docking score, surpassing both the reference drug (amantadine) and the co-crystallized ligand (LP4), primarily through hydrophobic interactions with PHE 46 residue. Compound **9** formed two hydrogen bonds with THR 122 and exhibited hydrophobic interaction with TYR 117, whereas compound **7** interacted hydrophobically with THR 122. Pharmacokinetic modeling using the BOILED-Egg model indicated that some compounds are likely to cross the blood-brain barrier (yellow region), while others remain in the white area. Impressively, the compounds also demonstrated desirable drug-likeness profiles. These findings suggest that the synthesized compounds hold promise as potent antiviral candidates.

**Supplementary Information:**

The online version contains supplementary material available at 10.1038/s41598-025-03495-6.

## Introduction

Newcastle disease (ND) is an acute highly contagious viral disease of poultry that affects the respiratory, nervous, and digestive systems, causing significant losses to the poultry industry^1^. Newcastle disease virus (NDV) is a viral pathogen capable of infecting more than 200 species of wild birds and domestic poultry^2^. Over the last decade, ND has caused continuous devastating effects on the global poultry industry; therefore, the world organization for animal health (WOAH) listed it as a notifiable terrestrial animal disease, emphasizing its international economic significance^3^. The incidence of ND has increased because of improper vaccination programs with vaccination failure, the presence of immunosuppressive diseases, and the genetic mutations of the virus, which lead to changes in biological characteristics and pathogenicity, even in vaccinated flocks^4^.

Poultry flocks encountered by ND are responsible for high rates of economic losses^5,6^. The disease is characterized by respiratory, neurological, and gastrointestinal symptoms, leading to losses including high mortality rate up to 100% in unvaccinated flocks, a drop in egg production (quantity and quality), weight loss, and growth retardation^7^. The symptoms of NDV usually appear within two to fifteen days after a bird gets infected, but sometimes it can rise to four weeks. In countries with large poultry industries, outbreaks of NDV resulted in millions of dollars in losses per outbreak. Based on their pathogenicity for chickens, NDV strains are classified into highly pathogenic (velogenic), intermediate (mesogenic), and apathogenic (lentogenic) strains^8,9^.

The global poultry industry plays a pivotal role in providing eggs and meat for human consumption. However, outbreaks of viral disease, especially NDV, within poultry farms have detrimental effects on various zootechnical parameters, such as body weight gain, feed intake, feed conversion ratio, as well as the quality of egg and meat production. Cases of vaccine failure have been reported in regions where highly pathogenic strains of NDV are prevalent. The disease is well-documented, with 20 genotypes and more than 236 susceptible avian species reported worldwide^10–12^. It spreads when infected birds release the virus through their mouth and rear opening, and other birds can catch it by breathing it in or eating it^13^. The presentation of NDV symptoms can vary based on the specific virus strain and the species of bird affected.

The ongoing challenge in the development of NDV vaccines lies in the ever-changing nature of genetically varied genotypes that are widely dispersed across different regions^14^. Understanding the structure and properties of NDV is crucial for developing effective antiviral strategies. Because the number of NDV cases is increasing and viruses are changing upon time, it is crucial to have effective ways of applying new chemicals as antivirals to control and prevent disease^2^. Commonly, vaccines including live attenuated and inactivated formulations are employed to protect poultry from NDV^15^. Considerable research efforts have been devoted to the detection of potent antiviral agents^16–20^.

The viral genome is around 15,200 base pairs (bp) in length and encodes six different proteins: nucleocapsid protein (NP); phosphoprotein (P); matrix protein (M); large RNA polymerase (L); fusion protein (F); and haemagglutinin-neuraminidase (HN). Two other proteins (V and W) could also be coded through P protein mRNA editing^21,22^. The phylogenetic analysis of F gene sequences of NDVs divided them into two classes (I and II): class I includes avirulent viruses, with a natural reservoir of aquatic wild birds, but one virulent isolate has been included^23^; whereas class II contains viruses that have higher genetic and virulence variability with 20 genotypes (I–XXI, except the recombinant sequence genotype XV) and are known to infect a wide range of domestic and wild birds^21,22,24^. NDV genotype VII is a highly pathogenic orthoavulavirus that has caused multiple outbreaks among poultry in Egypt since 2011.

Pyrazole compounds are a significant class of heterocyclic organic compounds imparting distinct chemical properties and biological activities, making pyrazoles a focal point of research in medicinal chemistry, agricultural chemistry, and material sciences^25–31^. The pyrazole ring system is a planar structure with considerable electron density around the nitrogen atoms. This configuration allows pyrazoles to engage in various chemical reactions, enabling the synthesis of wide derivatives with tailored properties. Pyrazole derivatives exhibit a wide spectrum of biological properties, making them valuable in the pharmaceutical industry. They are known for their antiviral, antitumor, antimicrobial, anti-inflammatory, and insecticidal properties^32–38^.

**Fig. 1:**
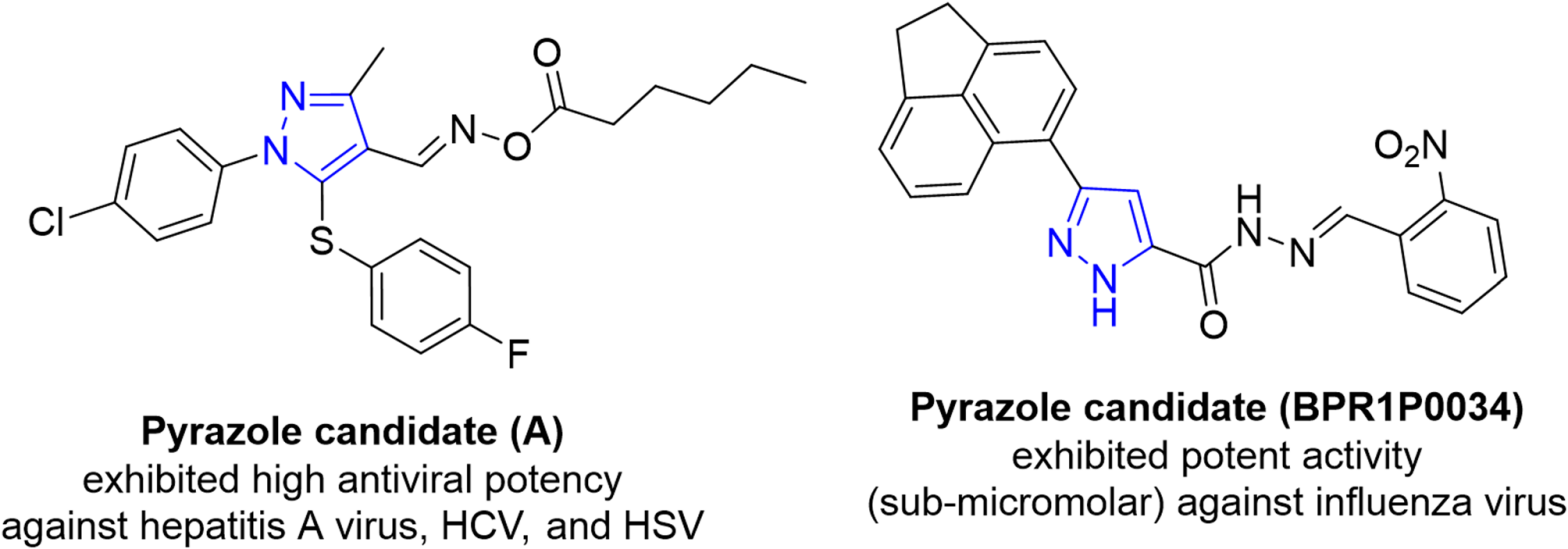
Antiviral agents (**A** and **BPR1P0034**) bearing a pyrazole core.

For instance, diverse pyrazole-containing systems exhibited potent antiviral therapeutics against targets like HIV (human immunodeficiency virus), HCV (hepatitis C virus), HSV-1 (Herpes simplex virus), NNRTI (Non-nucleoside reverse transcriptase inhibitors), H1N1 (a swine-origin influenza A), and H5N1 (Highly Pathogenic Avian Influenza A)^16,29,33,39^. Recently, antiviral research was primarily focused on the development of nucleoside and nonnucleoside analogues^16,40^. For example, pyrazole **(A)** exhibited a high antiviral potency against hepatitis A virus, HCV, and HSV (Fig. 1)^40,41^. Their unique chemical structure allows for extensive modification, leading to the development of compounds with specific and enhanced properties. Thus, substituted pyrazoles represent important key intermediates for the synthesis of therapeutically active drugs.

Outstanding research into pyrazole derivatives continues to discover new potential uses and benefits, solidifying their importance in science and industry. The design and incorporation of other pharmacophores with a pyrazole in the molecule might lead to innovative potent therapeutic agents. It was also reported that 1,3-diphenylpyrazole derivatives exhibited 95–100% protection of chicks against NDV^42^. Also, the pyrazole-including agent, BPR1P0034^43^ has potent activity (sub-micromolar) against the influenza virus (cf. Figure 1), which implies an opportunity for developing new effective antiviral agents. In this research and prompted by our strategy^44–53^, this work attempted to develop new nonnucleoside antiviral agents by synthesizing 4-substituted pyrazole candidates with potential antiviral activity against ND virus in specific pathogen free (SPF) chicken embryos and immune boosting properties of these substances in SPF chicks. supported by molecular docking simulation and modeling pharmacokinetics studies.

### Rationale and design

**Fig. 2:**
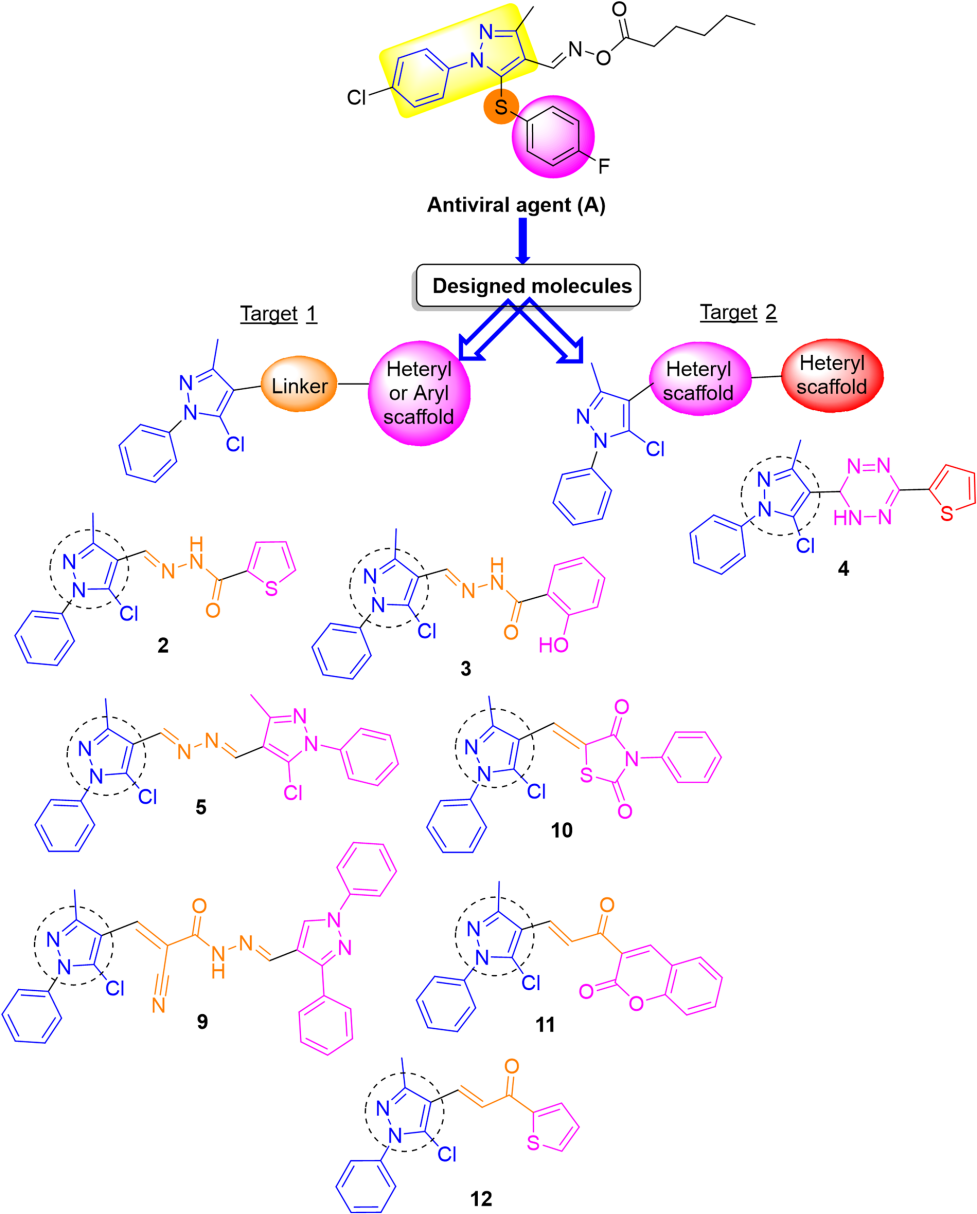
Rationale and design of the chemical structures of antiviral agent (A) and target candidates.

Our rationale and design were based on a structural diversification through conserving the pyrazole core and incorporation of certain side chains or heterocyclic moieties, to attain potential antiviral efficacy. Pyrazole has been described as a crucial scaffold due to its presence in many pharmacologically active compounds. In our search to develop prospective antiviral compounds, insight was drawn from pyrazole’s pharmacophoric features, which have shown antiviral efficacy (cf. Figure 2). To design and produce a new series of 4-substituted pyrazole derivatives, the fundamental pharmacophoric features of pyrazole were kept while heterocyclic and linker side chain moieties were integrated, which are as follows: (i) A pyrazole core (chromophore), which was linked with antiviral activity, was retained in the design, (ii) The linker was modified by incorporating hydrazonyl or α,β-unsaturated carbonyl scaffolds in order to establish more hydrogen bonds during interaction with virus proteins, (iii) To increase the antiviral activity of these candidates, cores of thiophene, tetrazine, pyrazole, thiazolidine, and coumarin were incorporated to the design, which were found to have promising effects in this view. By linking these features, this work intended to produce some 4-substituted pyrazole derivatives with improved antiviral effects (Fig. 2).

## Results and discussion

### Chemistry

**Fig. 3:**
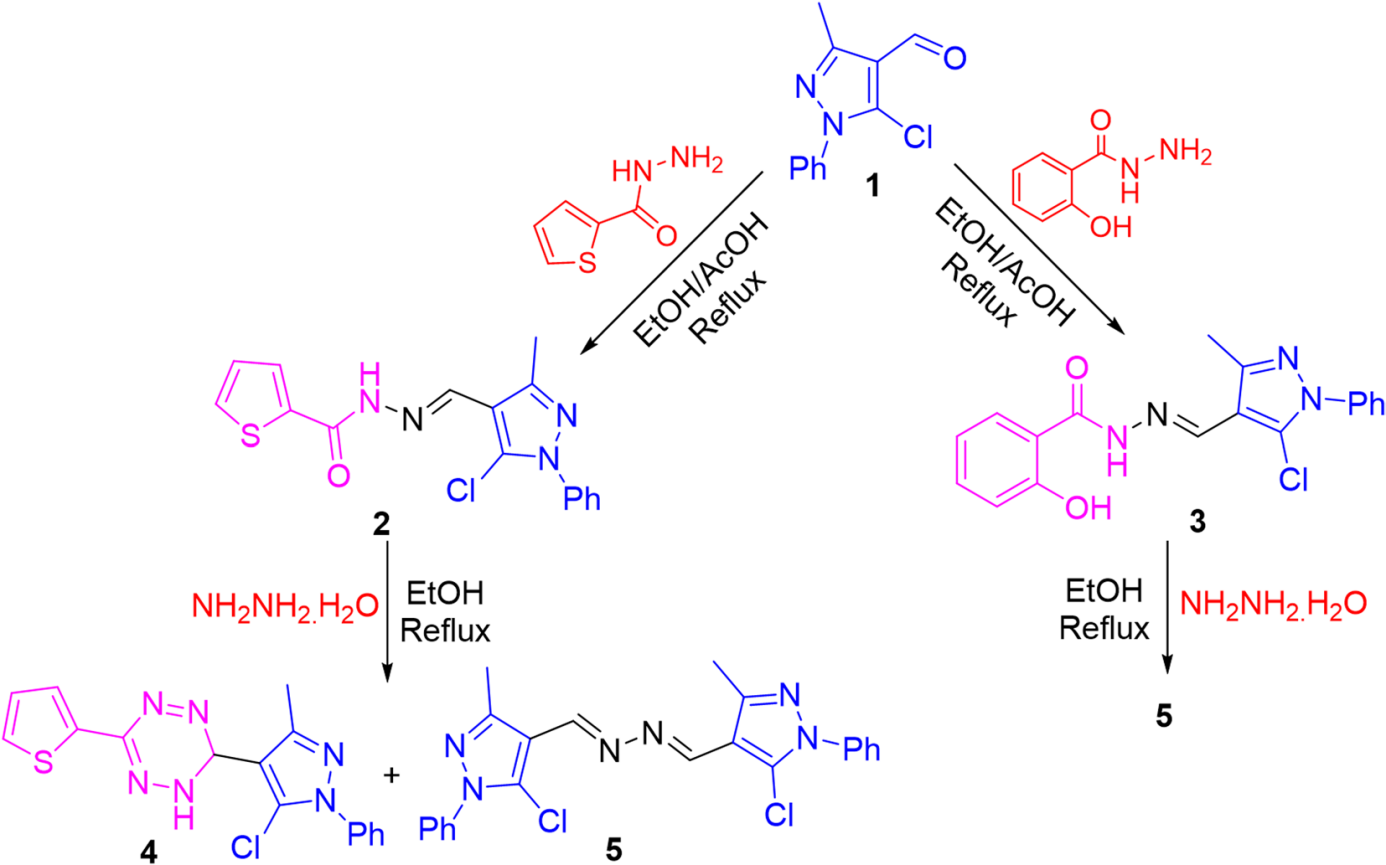
Synthesis of two hydrazones and their behavior toward hydrazine hydrate.

**Fig. 4:**
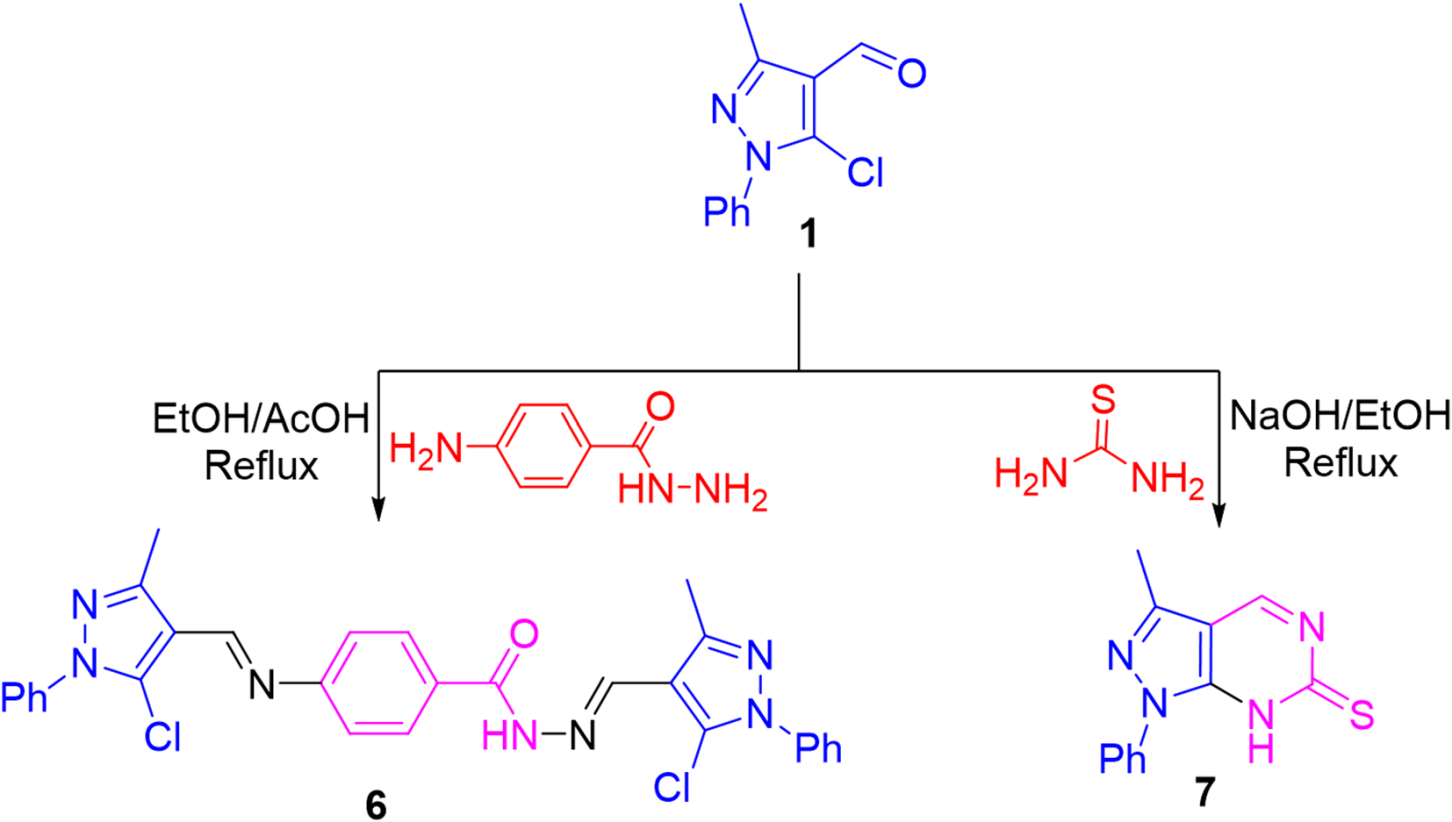
Condensation of pyrazole aldehyde **1** with 4-aminobenzohydrazide and thiourea.

The key aldehyde, 5-chloro-4-formyl-3-methyl-1-phenylpyrazole **1**^54^ was allowed to react with various nitrogen and carbon-centered nucleophiles to obtain diverse pyrazole-based candidates (cf. Figures 3 and 4). First, condensation of aldehyde **1** with thiophene-2-carbohydrazide and 2-hydroxybenzohydrazide afforded the corresponding hydrazone candidates **2** and **3**, respectively. Absorption bands for NH, C = O, and C = N groups appeared in IR of hydrazone **2**, while in hydrazone **3**, an extra band for OH group appeared. ^1^H NMR of hydrazone **2** displayed three characteristic singlet signals as follows: the first is integrated for three protons in the upfield region corresponding to methyl proton, the second is integrated for one proton in the downfield region corresponding to methine proton (CH = N), the third is an exchangeable signal in the downfield corresponding to NH proton.

Hydrazinolysis of hydrazone **2** with hydrazine hydrate in refluxing ethanol produced a mixture of tetrazine **4** and bis-pyrazolylhydrazine **5** derivatives. The ^1^H NMR of tetrazine derivative **4** displayed two singlet signals corresponding to CH and NH of tetrazine moiety, in addition to a singlet signal of methyl protons. Otherwise, hydrazinolysis of **3** under the same conditions afforded the bis-pyrazolylhydrazine derivative **5**. The later compound was confirmed by comparison with an authentic sample prepared from refluxing an ethanolic solution of aldehyde **1** with hydrazine hydrate^55^.

Noteworthy, treating aldehyde **1** with 4-aminobenzohydrazide in refluxing ethanol under acid catalyzed media achieved the formation of bi-condensation product **6**. The IR of **6** lacked NH_2_ absorption and displayed carbonyl absorption. Further, its ^1^H NMR disclosed two singlet signals for two methine protons (2 CH = N) in addition to an exchangeable singlet signal for NH proton. In turn, refluxing aldehyde **1** with thiourea under basic conditions produced the heteroannulated product, pyrazolopyrimidine derivative **7** ^56^ (cf. Figure 4). Its ^1^H NMR showed a singlet signal for a methine proton (CH = N) in the downfield region and an exchangeable singlet signal corresponding to NH proton. The mass spectra of the compounds strongly supported the proposed structures.

On the other hand, treating aldehyde **1** with pyrazole-cyanoacetohydrazone^57^ in 1,4-dioxane and piperidine at room temperature produced α,β-unsaturated nitrile **8** as yellow crystals. In its IR chart, the absorption band of nitrile functionality appeared at relatively lower value (ν 2206 cm^− 1^), which was attributed to the conjugation with C = C moiety. Its ^1^H NMR displayed an exchangeable singlet for NH proton and a singlet signal for C5-H of pyrazolyl moiety. The space models of the isomeric structures of compound **8** confirmed the *Z*-configuration for the new olefinic double bond, which was probably due to the steric and field effects between the nitrile and pyrazole moieties (cf. Table S1 in S1_file).

Likewise, condensation of aldehyde **1** with 2,4-dioxo-3-phenylthiazolidine in refluxing acetic acid and fused sodium acetate afforded α,β-unsaturated carbonyl compound **9**. The space models of the isomeric structures of compound **9** confirmed the *E*-configuration for the new olefinic double bond, which was probably due to the steric effect of the pyrazole moiety (cf. Table S1).

**Fig. 5:**
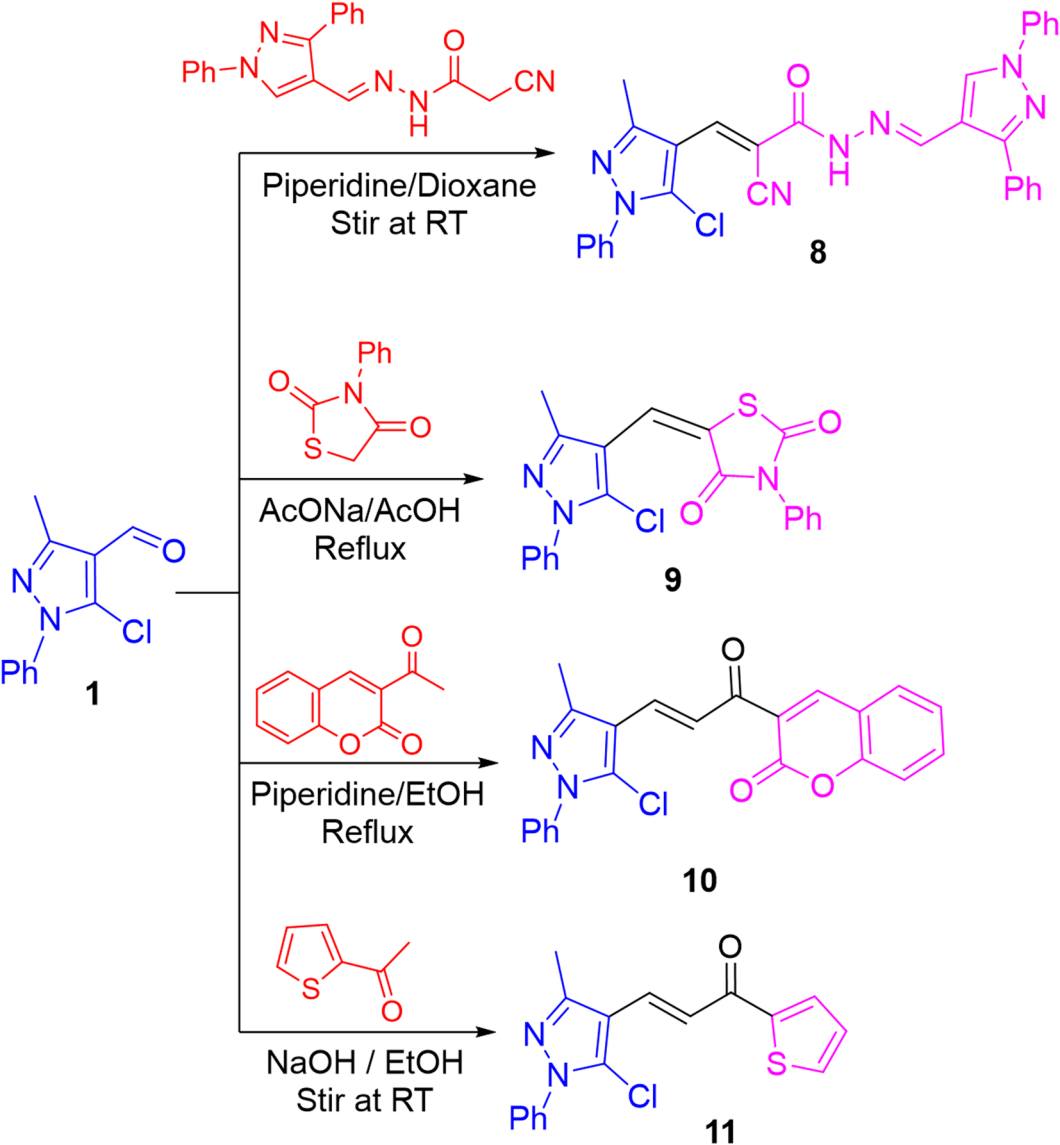
Condensation of pyrazole aldehyde **1** with some carbon nucleophiles.

From the biological and synthetic point of view, chalcones are known to be crucial intermediates in organic synthesis serving as building blocks for many heterocyclic systems with pharmacological importance^30,46^. Also, based on the biological potential of coumarin and thiophene derivatives^58–60^, chalcone derivatives bearing a moiety of coumarin **10** and thiophene **11** were efficiently synthesized upon treating the aldehyde **1** with 3-acetylcoumarin and 2-acetylthiophene, respectively (cf. Figure 5). The carbonyl of coumarin moiety (in compound **10**) appeared in its IR spectrum at ν 1721 cm^− 1^. Also, in its ^1^H NMR spectrum, a singlet signal for C4-H of coumarin moiety was displayed at δ 8.59 ppm. Their mass spectra provided the molecular ion peaks besides M + 2 isotopic peaks. The space models of the isomeric structures of chalcones **10** and **11** confirmed the *E*-configurations for the olefinic double bond, which were probably due to the steric effects of the pyrazole moiety with carbonyl group (cf. Table S1).

### Antiviral activity evaluation

Aiming to treat Newcastle disease, many vaccines have been appointed against NDV based on inactivated and attenuated viruses but suited useless due to the genetic changes in the virus. NDV genotype VII is a highly pathogenic Orthoavulavirus that has caused multiple outbreaks among poultry in Egypt since 2011. Haemagglutination is the aggregation of RBCs (red blood cells) in suspension with the presence of certain haemagglutinating virus particles. This phenomenon is a result of the attachment of specific outer viral peplomeres (haemagglutinins) with specific receptors present on the surface of RBCs. This characteristic feature can be used in the detection of viruses. The inhibition of haemagglutination caused by these viruses is the detection of their activity’s inhibition.

Amantadine was selected as the antiviral reference drug due to its well-established mechanism of action as a viral M2 ion channel blocker, primarily used against influenza A virus. Its ability to interfere with viral uncoating and replication makes it a valuable tool for studying early stages of the viral life cycle. Additionally, Amantadine has been reported to exhibit broader antiviral and neuroprotective properties, including modulatory effects on host cellular pathways such as autophagy and ion channel function. These characteristics make it an appropriate reference compound for evaluating antiviral efficacy and potential host-virus interactions in the present study.

Toxicity assays conducted on embryonated chicken SPF eggs revealed that the 50% cytotoxic concentration (CC_50_) of the tested compounds ranged from 200 to 800 µL per egg (cf. Table 1). The 50% inhibitory concentration (IC_50_) was determined as the concentration required to completely inhibit viral effects in 50% of the eggs. The selectivity index (SI) for each compound was calculated as a ratio of CC_50_ to IC_50_^57^. The tested compounds demonstrated promising antiviral activity against NDV. When embryonated eggs were inoculated with a mixture of NDV and each compound individually, virus replication was notably impacted. Serial 12-fold dilutions (10^1^ to 10^12^) of the mixtures were applied, and the allantoic fluid from each group received varying concentrations. The results showed a reduction in virus titer (cf. Table 2).

**Table 1:** The different CC_50_, IC_50_, and SI values in embryonated chicken SPF eggs and their embryonic toxicity against NDV.

Compds.	^a^CC_50_	^b^IC_50_	^c^SI (%)
**2**	> 800	≤ 10	80
**3**	> 700	≤ 8	87.5
**4**	> 400	≤ 5	80
**6**	> 200	≤ 2	100
**7**	> 500	≤ 5.5	90.9
**9**	> 300	≤ 3	100
**10**	> 800	≤ 9	88
**11**	> 500	≤ 6	83
**Amantadine**	> 300	≤ 3	100

*NDV* newcastle disease virus.

^a^CC_50_: Cytotoxicity concentration fifty.

^b^IC_50_: Inhibitory concentration fifty ^c^ SI: Selectivity index.

**Table 2:** Haemagglutination (HA) activity of the tested compounds against NDV in embryonated chicken eggs (ECES) using serial 12-fold virus dilutions (10^1^ to 10^12^).

Virus dilution	NDV alone	Number of embryos (out of 5)						
		Compounds						
		4	6	7	9	10	11	Amantadine
10^1^	5/5	5/5	5/5	5/5	5/5	5/5	5/5	5/5
10^2^	5/5	5/5	5/5	5/5	5/5	5/5	5/5	5/5
10^3^	5/5	5/5	5/5	5/5	5/5	5/5	5/5	5/5
10^4^	5/5	5/5	5/5	5/5	5/5	5/5	5/5	5/5
10^5^	5/5	5/5	5/5	5/5	5/5	5/5	5/5	5/5
10^6^	5/5	5/5	4/5	5/5	3/5	5/5	5/5	5/5
10^7^	4/5	4/5	3/5	4/5	3/5	4/5	4/5	4/5
10^8^	3/5	3/5	2/5	2/5	2/5	3/5	3/5	2/5
10^9^	3/5	3/5	1/5	2/5	1/5	3/5	2/5	1/5
10^10^	1/5	1/5	0/5	0/5	0/5	1/5	1/5	0/5
10^11^	0/5	0/5	0/5	0/5	0/5	0/5	0/5	0/5
10^12^	0/5	0/5	0/5	0/5	0/5	0/5	0/5	0/5
HA-titer	10^10^	10^9^	10^8.5^	10^8.6^	10^8.5^	10^8.7^	10^10^	10^8.5^

Each row represents the HA results following serial 12-fold virus dilutions (10^1^ to 10^12^). Columns indicate the number of embryos (out of 5) showing HA activity after inoculation with NDV alone or with mixtures of NDV and individual test compounds and amantadine as a reference antiviral agent. HA activity was assessed in the allantoic fluid after incubation. The HA titer represents the last dilution at which HA was observed in at least one of the five eggs. A decrease in HA titer compared to the virus control (NDV alone) indicates inhibition of viral replication by the compound.

### Bioassay of immune boosting properties of different compounds in SPF chicks

Using amantadine as a reference, the tested compounds were screened for their mortality and protection percentages using day post challenge (DPC) against NDV with vaccinated group (20 chicks). The mortality percentage was calculated from 1st to 6th DPC as (the number of dead birds / total number of birds) × 100. Thus, the protection percent of the vaccine was calculated from 1st to 6th DPC as (the number of live birds / total number of birds) × 100. Also, the protection percent can be calculated as (100 – Mortality). The results revealed that hydrazone **6** and thiazolidinedione derivative **9** exhibited 0% mortality and 100% protection against NDV while pyrazolopyrimidine **7** showed 95% protection (5% mortality). In turn, tetrazine derivative **4** disclosed 85% protection (15% mortality) and chalcone **11** exhibited 80% protection (20% mortality) (Table 3).

Blood samples were collected individually from jugular vein at 28 days post vaccination for potency test by calculation of the HI antibody titer in serum of vaccinated chicks (20 chicks). Haemagglutination inhibition (HI) titer values^62^ of the tested compounds with respect to amantadine as a standard compound were displayed in Table 4. On the other hand, an immunostimulant is any substance that enhances or stimulates the body’s immune response. Immunostimulants can be natural (like certain bacterial components) or synthetic (like specific drugs or compounds), and they work by activating various components of the immune system.

Comparison of humeral response of the vaccinated group (first group vaccinated only) and other vaccinated groups, which separately received the tested compounds as immunostimulant, revealed that hydrazone **6** has special immune boosting properties as it elevates the antibody titer in the serum of vaccinated chicks. Accordingly, hydrazone and thiazolidinedione derivatives would be considered promising antiviral candidates and can be added to NDV vaccines to raise the treatment percentage and then decrease the mortalities and the economics losses. Also, this prevents disease introduction, reduces susceptibility over time, and facilitates the export of poultry products.

**Table 3:** Mortality and protection percentages against NDV challenge virus using day post challenge (dead birds / total number of birds).

Vaccinated groups (20 chicks)	1st DPC*	2nd DPC	3rd DPC	4th DPC	5th DPC	6th DPC	Number of dead birds	Mortality %	Protection %
**4**	0/20	2/20	1/20	0/20	0/20	0/20	3	15	85
**6**	0/20	0/20	0/20	0/20	0/20	0/20	0	0	100
**7**	0/20	0/20	0/20	0/20	1/20	0/20	1	5	95
**9**	0/20	0/20	0/20	0/20	0/20	0/20	0	0	100
**11**	0/20	1/20	2/20	1/20	0/20	0/20	4	20	80
**+ve control Vaccinated group only**	0/20	0/20	0/20	1/20	0/20	0/20	1	5	95
**-ve Control group chicks**	0/20	4/20	5/20	6/20	4/20	1/20	20	100	0
**Amantadine**	0/20	0/20	1/20	1/20	0/20	0/20	2	10	90

*DPC day post challenge.

**Table 4:** Haemagglutination Inhibition (HI) titer values of the tested compounds with respect to amantadine as a standard compound.

Compds.	HI titer
**4**	6.7 log2
**6**	7.1 log2
**7**	6.8 log2
**9**	7.0 log2
**10**	6.4 log2
**11**	6.6 log2
Amantadine	7.1 log2
Vaccinated group only	6.6 log2

### Molecular Docking simulation

A molecular docking approach was applied to offer the possible antiviral mechanism of action through displaying the interaction of a compound with active pockets of the proper target^63,64^. The immunostimulant property refers to the ability of LPS (lipopolysaccharide) to prompt a strong immune response. LPS is a component of the outer membrane of Gram-negative bacteria and is recognized by the innate immune system as a pathogen-associated molecular pattern (PAMP). LPS exerts the immunostimulant effect through:

(i) Recognition by Immune Receptors: LPS binds to Toll-like receptor 4 (TLR4) on immune cells, specifically in complex with MD-2, a co-receptor that helps in LPS recognition.
(ii) Signal Transduction: Upon binding, this complex initiates a signaling cascade inside the immune cell, leading to the activation of transcription factors such as NF-κB.
(iii) Cytokine Production: This signaling results in the production of pro-inflammatory cytokines (e.g., TNF-α, IL-6, IL-1β) that help combat infections by recruiting and activating more immune cells.
(iv) Innate Immune Activation: LPS acts rapidly, stimulating the innate immune system, which serves as the first line of defense against pathogens.

Because of this ability to potently activate immune responses, LPS is often used experimentally to mimic infection or to test the immune-stimulating capacity of compounds. However, excessive stimulation by LPS can also lead to hyperinflammation or sepsis, so its effects must be carefully studied. The host immune response against LPS is prompted by myeloid differentiation factor 2 (MD-2) in association with Toll-like receptor 4 (TLR4) on the cell surface^65^. The MD-2/TLR4-mediated LPS response is regulated by the evolutionarily correlated complex of MD-1 and Toll-like receptor homolog RP105 through a hydrophobic cavity between the two beta-sheets. A lipid-like moiety was observed inside the cavity, proposing the possibility of a direct MD-1/LPS interaction.

In molecular docking or drug design studies, compounds interacting with TLR4 or LPS-binding pathways are evaluated for their potential immunomodulatory or antiviral effects by influencing these immune activation pathways. The immune receptor TLR4 was chosen for molecular docking due to its critical role in innate immunity and its ability to recognize pathogen-associated molecular patterns including LPS. As a key receptor in mediating inflammatory responses, TLR4 activation has been implicated in the pathogenesis of various infections. Targeting TLR4 through docking studies allows the exploration of potential interactions that may modulate it signaling pathway, offering insight into therapeutic strategies aimed at controlling excessive immune dysregulation.

Thus, molecular docking analysis of the promising pyrazole-based compounds **6**,** 7**, and **9** was investigated against target immune receptor TLR4 (PDB ID: 3MU3) active sites, aiming to account for LPS-induced responses. The binding affinity was measured by the binding energy (S-score, kcal/mol) and hydrogen bonds. For better understanding, the reference drug (amantadine) was also docked into the TLR4 active pockets. All complexes were docked in the same groove of binding pocket of the native co-crystallized ligand (LP4) (Tables 5 and 6). Table 6 displays the amino acids involved in binding interactions between compounds and active sites of protein including hydrogen bonding and hydrophobic interactions. Also, Table 6 offers 2D and 3D representation interactions of the promising compounds with the target TLR4 enzyme.

The docking results showed that the amino acid residues PHE 46, THR 122, and TYR 117 were implicated in receptor binding through hydrogen bonds and hydrophobic interactions. Herein, the key amino acid THR 122 was common in the interactions with the reference (amantadine) and co-crystallized ligands (LP4) as depicted in Table 5. Thus, compound **6** exhibited the best docking score (-7.2322 kcal/mol) compared to amantadine (-4.1456 kcal/mol) and co-crystallized ligand (-9.7099 kcal/mol). The interaction of **6** with target TLR4 was through hydrophobic interaction with PHE 46 amino acid. Next, compound **9** exhibited binding energy of -6.1678 kcal/mol through two hydrogen bonding with the key amino acid THR 122 and hydrophobic interaction with TYR 117. Otherwise, compound **7** showed hydrophobic interaction with THR 122.

**Table 5:** Docking results and binding amino acids with compounds **6**,** 7**, and **9** to TLR4 active sites compared to amantadine and LP4.

Compds.	S-score (kcal/mol)	RMSD (Å)	Ligand	Amino acids involved in bonding*	
				H-bonding (Bond length, Å)	Hydrophobic interaction (Bond length, Å)
**6**	-7.2322	1.4510	6-ring	-	PHE 46 (3.79)
**7**	-5.1555	0.7509	5-ring	-	*THR 122* (4.02)
**9**	-6.1678	1.7057	O 6 O 6 C 8	*THR 122* (3.29) *THR 122* (2.85) -	- - TYR 117 (4.60)
**Amantadine**	-8.1456	1.8184	N 26 N 26	*THR 122* (3.47) *THR 122* (3.12)	-
**Co-crystallized ligand (LP4)**	-9.7099	1.7208	N2 6 O44 100 O42 97 O42 97 O47 104 O47 104 C25 53	*THR 122* (2.86) GLU 94 (2.64) *THR 122* (2.95) *THR 122* (2.74) LEU 104 (3.24) ILE 105 (2.74)	- - - - - - PHE 48 (4.47)

*The common interacting amino acids are italicized.

### Modeling pharmacokinetics

**Fig. 6:**
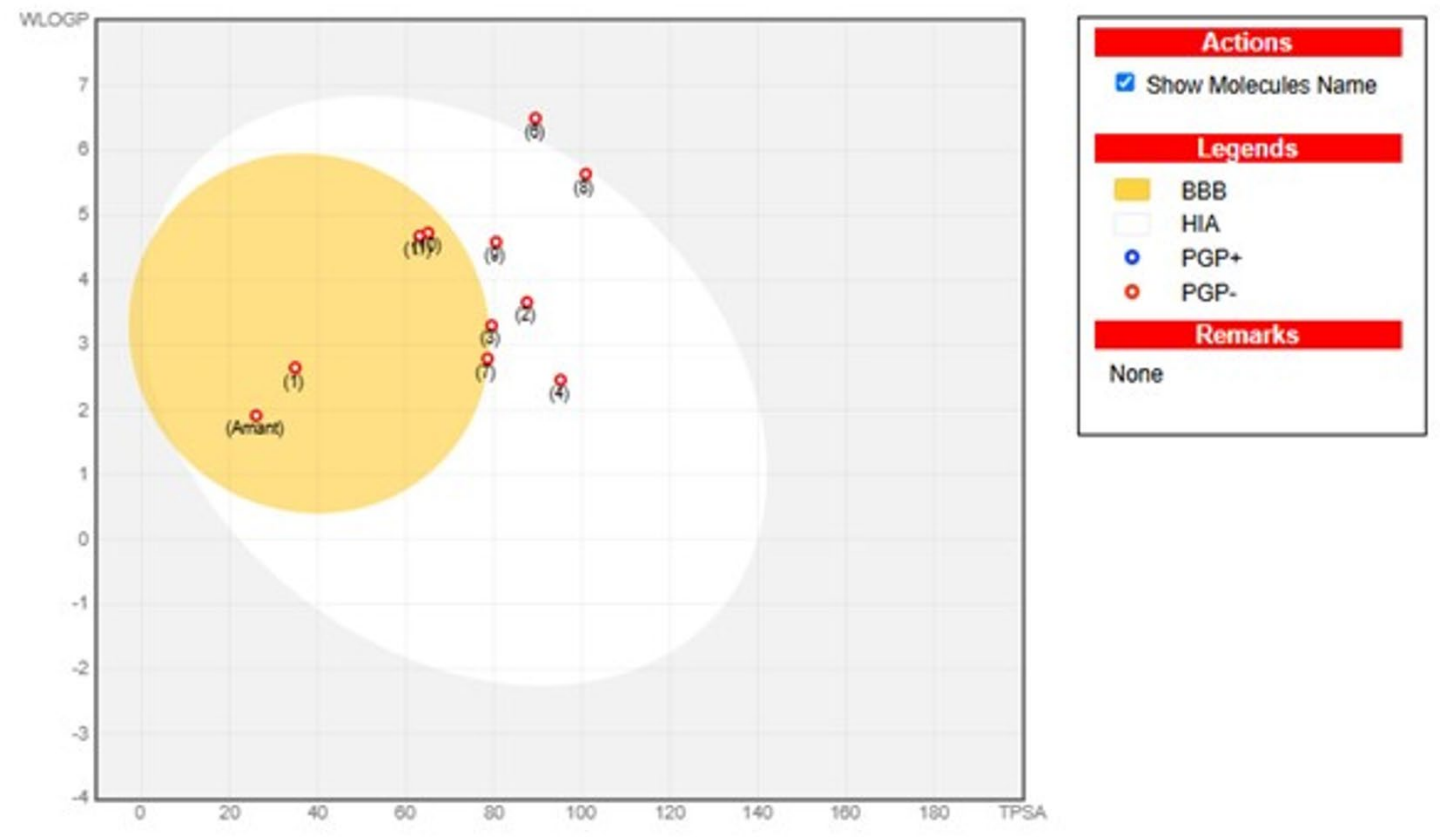
BOILED-Egg chart of compounds **1–11** with respect to Amantadine (Amant).

The pharmacokinetics properties of the prepared candidates **1–11** were assessed utilizing the SwissADME free web tool compared to the reference, Amantadine (cf. Figures S1-S11 in S1_file and Table S2 in S3_file). A thorough analysis of ADME (absorption, distribution, metabolism, and excretion) profiles and key physicochemical properties of compounds in addition to their drug-likeness evaluations offered a comprehensive recognizing of their pharmacological properties^66,67^. ADME analysis is also worthy insights into the biological behavior of compounds. All compounds are assessed as non-inhibitors of CYP2D6, and hence side effects (i.e., liver dysfunction) are not anticipated upon their administration. Recognizing the absorption properties, compounds **2**,** 3**,** 4**,** 8**, and **9** uncovered gastrointestinal tract (GIT) absorption upon its being in the BOILED-Egg white area chart as shown in Fig. 6. Whilst compounds **1**,** 7**,** 10**, and **11** were presented in the chart yellow area and predicted to penetrate the blood-brain barrier. They are not potential substances for permeability glycoprotein (PGP), indicated by red. Thus, these compounds revealed desirable drug-likeness.

The pharmacokinetics and antiviral assessment of the examined compounds, compared to Amantadine, showed crucial factors affecting their potential activity. These key factors include TPSA (topological polar surface area), lipophilicity (Consensus Log P_o/w_), and GI (gastrointestinal) absorption, which affect their bioavailability at the target place. The comparison of these key factors and antiviral SI values of the examined compounds was depicted in Table 7. Typically, lower TPSA values are associated with better membrane permeability, enhancing compound’s antiviral activity. Compared to Amantadine, all compounds exhibited favorite TPSA values (< 140 Å^2^. The slightly higher TPSA values of some compounds than Amantadine suggested other factors like lipophilicity or specific interactions with receptors, may reinforce any potential decrease in permeability. Also, all compounds showed Consensus Log P_o/w_ values higher than Amantadine. For example, compound **6** exhibited TPSA value of 89.46 Å^2^ and low GI absorption but it had the highest Consensus Log P_o/w_ value (5.64), improving its lipophilic interactions and antiviral activity.

**Table 6:** The 2D and 3D interactions of compounds **6**, **7**, and **9** with TLR4 active sites compared to amantadine and co-crystallized ligand (LP4).

Compds.	2D	3D
**6**		
**7**		
**9**		
**Amantadine**		
**Co-crystallized ligand (LP4)**		

*The docked compounds were colored in cyan, while amantadine and LP4 were colored in green and purple, respectively.

**Table 7:** Judgement of SI values and key ADME properties of the examined compounds and amantadine.

Compds.	SI*	TPSA**	Consensus Log *P*_o/w_	GI absorption	Bioavailability score
**2**	80	87.52	3.50	High	0.55
**3**	87.5	79.51	3.09	High	0.55
**4**	80	95.17	4.01	High	0.55
**6**	100	89.46	5.64	Low	0.17
**7**	90.9	78.59	2.47	High	0.55
**9**	100	80.50	4.09	High	0.55
**10**	88	65.10	4.40	High	0.55
**11**	83	63.13	4.31	High	0.55
**Amantadine**	100	26.02	2.18	High	0.55

*SI: Selectivity index ** TPSA: Topological polar surface area.

## Conclusion

A series of pyrazole-based candidates was prepared upon treatment of 5-chloro-4-formyl-3-methyl-1-phenylpyrazole with certain nitrogen and carbon-centered nucleophiles. The antiviral activity of the prepared compounds was examined against NDV, which revealed that hydrazone **6** and thiazolidinedione derivative **9** exhibited 0% mortality and 100% protection against NDV while pyrazolopyrimidine derivative **7** displayed 95% protection. In turn, tetrazine derivative **4** showed 85% and chalcone **11** exhibited 80% protection. These results were supported by molecular docking simulation with TLR4 enzyme. The docking results showed that compound **6** exhibited the best docking score (-7.2322 kcal/mol) compared to the reference agent, amantadine (-8.1456 kcal/mol) and co-crystallized ligand, LP4 (-9.7099 kcal/mol), through hydrophobic interaction with PHE 46 amino acid. While compound **9** exhibited binding energy of -6.1678 kcal/mol through two hydrogen bonding with THR 122 and hydrophobic interaction with TYR 117. Otherwise, compound **7** showed hydrophobic interaction with THR 122. Regarding modeling pharmacokinetics, all compounds exhibited favorite TPSA values and compounds **1**,** 7**,** 10**, and **11** were presented in the BOILED-Egg chart yellow area and predicted to penetrate the blood-brain barrier, exhibiting desirable drug-likeness and oral bioavailability properties. Thus, hydrazone and thiazolidinedione derivatives would be considered promising antiviral candidates and can be added to NDV vaccines to raise the treatment percent and then decrease the mortality percent and the economics losses.

## Materials and methods

Melting points were measured in open capillary tubes on a MEL-TEMP II electrothermal melting point apparatus. The elemental analyses were performed on a Perkin-Elmer 2400 CHN elemental analyzer (Perkin-Elmer, Waltham, MA) at Faculty of Science, Ain Shams University. Infrared spectra (ν, cm^−1^) were recorded utilizing KBr wafer technique on Fourier Transform Infrared Thermo Electron Nicolet iS10 Spectrometer (Thermo Fisher Scientific Inc. Waltham, MA) at Faculty of Science, Ain Shams University. Electron impact mass spectra (EIMS) were run on direct probe controller inlet part to single quadrupole mass analyzer in Thermo Scientific GC-MS MODEL (ISQ LT) using Thermo X-CALIBUR software at regional center for mycology and biotechnology (RCMB), Al-Azhar University, Cairo, Egypt. ^1^H NMR spectra (δ, ppm) were recorded on BRUKER 400 *MHz* Spectrometer at Faculty of Pharmacy, Ain Shams University, with tetramethyl silane as an internal standard, using DMSO-*d*_6_ as a solvent (Figures S12-S50). Thin-layer chromatography (TLC) was run utilizing TLC aluminum sheets silica gel 60 F_254_ (Merck, Whitehouse Station, NJ).

### N’-((5-Chloro-3-methyl-1-phenyl-1H-pyrazol-4-yl)methylene)thiophene-2-carbohydrazide (2)

A solution of aldehyde **1** ^54^ (0.01 mol) and thiophene-2-carbohydrazide (0.01 mol) in absolute ethyl alcohol (25 mL) and glacial acetic acid (0.2 mL) was refluxed for 4 h. The formed precipitate was filtered and recrystallized from ethyl alcohol to produce yellow crystals; mp. 178–180 ^o^C; yield 91%. IR (KBr, ν, cm^−1^): 3274 (NH), 3075, 3063 (CH aromatic), 2929 (CH aliphatic), 1657 (C = O), 1608 (C = N). ^1^H NMR (400 *MHz*, DMSO-*d*_*6*_) *δ* (ppm): 2.50 (s, 3 H, CH_3_), 7.22 (t, 1 H, CH, *J* = 7.5 *Hz*), 7.52–7.62 (m, 5 H, Ar-H), 7.89 (d, 1 H, CH, *J* = 7.8 *Hz*), 8.13 (d, 1 H. CH, *J* = 8.4 *Hz*), 8.45 (s, 1 H, CH = N), 11.81 (*br*.s, 1 H, NH). EIMS (70 eV, *m/z*, %): 346.40 (M + 2, 5), 344.36 (M^+^^.^, 17), 340.39 (73), 313.47 (47), 262.22 (100), 248.32 (68), 234.45 (63), 100.27 (49), 77.22 (74), 56.17 (61). Anal. Calcd for C_16_H_13_ClN_4_OS (344.82): C, 55.73; H, 3.80; N, 16.25; Found: C, 55.60; H, 3.74; N, 16.27%.

### N’-((5-Chloro-3-methyl-1-phenyl-1H-pyrazol-4-yl)methylene)-2-hydroxybenzohydrazide (3)

A solution of aldehyde **1** (0.01 mol) and 2-hydroxybenzohydrazide (0.01 mol) in absolute ethyl alcohol (25 mL) and glacial acetic acid (0.2 mL) was refluxed for 4 h. The formed precipitate was filtered and recrystallized from ethyl alcohol to give yellow crystals; mp. 198–200 ^o^C; yield 78%. IR (KBr, ν, cm^−1^): 3350 (*br*. OH), 3209 (NH), 3073 (CH aromatic), 2926 (CH aliphatic), 1638 (C = O), 1605 (C = N). ^1^H NMR (400 *MHz*, DMSO-*d*_*6*_) *δ* (ppm): 2.51 (s, 3 H, CH_3_), 6.97 (d, 1 H, Ar-H, *J* = 7.4 *Hz*), 7.44–7.61 (m, 7 H, Ar-H), 7.89 (d, 1 H, Ar-H, *J* = 7.2 *Hz*), 8.49 (s, 1 H, CH = N), 11.83 (*br*.s, 1 H, NH), 11.97 (*br*.s, 1 H, OH). EIMS (70 eV, *m/z*, %): 356.13 (M + 2, 4), 354.15 (M^+^^.^, 14), 351.24 (33), 348.46 (38), 305.11 (41), 283.89 (79), 272.68 (42), 269.42 (100), 160.23 (53), 111.51 (39), 78.79 (37), 69.08 (35). Anal. Calcd for C_18_H_15_ClN_4_O_2_ (354.79): C, 60.94; H, 4.26; N, 15.79; Found: C, 60.82; H, 4.20; N, 15.80%.

### Hydrazinolysis of hydrazone 2

A mixture of hydrazone **2** (0.01 mol) and hydrazine hydrate (0.015 mol, 80%) in absolute ethyl alcohol (25 mL) was refluxed for 4 h. The formed precipitate while heating was filtered and recrystallized from ethyl alcohol to afford *1*,*2-bis((5-chloro-3-methyl-1-phenyl-1 H-pyrazol-4-yl)methylene)hydrazine* **(5)** as beige crystals, mp. 249–251 °C [Lit^55^ mp, 250–252 ^o^C] (Identity: mp., mixed mp., TLC, and IR). The mother liquor was concentrated and then left to stand at room temperature. The precipitated solid was filtered and recrystallized from petroleum ether (60–80) to give tetrazine derivative **4**.

### 6-(5-Chloro-3-methyl-1-phenyl-1H-pyrazol-4-yl)-3-(thiophen-2-yl)-1,6-dihydro-1,2,4,5-tetrazine (4)

Yellow crystals; mp. 200–202 °C; yield 38%. IR (KBr, ν, cm^− 1^): 3177 (NH), 3062, 3027 (CH aromatic), 2955, 2922 (CH aliphatic), 1625 (C = N). ^1^H NMR (400 *MHz*, DMSO-*d*_*6*_) *δ* (ppm): 2.51 (s, 3 H, CH_3_), 4.26 (s, 1 H, CH), 7.14 (t, 1 H, Ar-H, *J* = 7.2 *Hz*), 7.26 (m, 1 H, Ar-H), 7.53–7.74 (m, 6 H, Ar-H), 8.59 (*br*.s, 1 H, NH). EIMS (70 eV, *m/z*, %): 358.02 (M + 2, 12), 356.07 (M^+^^.^, 38), 341.21 (49), 306.33 (37), 283.93 (100), 228.17 (41), 200.33 (31), 132.35 (21), 98.13 (17), 95.51 (35), 83.23 (51). Anal. Calcd for C_16_H_13_ClN_6_S (356.83): C, 53.86; H, 3.67; N, 23.55; Found: C, 53.72; H, 3.59; N, 23.58%.

### (E/Z)-N’-((5-Chloro-3-methyl-1-phenyl-1H-pyrazol-4-yl)methylene)-4-(((5-chloro-3-methyl-1-phenyl-1 H-pyrazol-4-yl)methylene)amino)benzohydrazide (6)

A solution of aldehyde **1** (0.01 mol) and 4-aminobenzohydrazide (0.01 mol) in absolute ethyl alcohol (25 mL) and glacial acetic acid (0.2 mL) was refluxed for 4 h. The formed precipitate was filtered and recrystallized from ethanol to give yellow crystals; mp. 226–228 °C; yield 77%. IR (KBr, ν, cm^−1^): 3210 (NH), 3059 (CH aromatic), 2970 (CH aliphatic), 1669 (C = O). ^1^H NMR (400 *MHz*, DMSO-*d*_*6*_) *δ* (ppm): 2.32 (s, 6 H, 2 CH_3_), 7.40–8.02 (m, 14 H, Ar-H), 8.49 (s, 1 H, *CH* = N-NH), 8.67 (s, 1 H, CH = N), 11.78 (*br*.s, 1 H, NH). EIMS (70 eV, *m/z*, %): 560.21 (M + 4, 15), 558.34 (M + 2, 7), 556.78 (M^+^^.^, 22), 467.06 (39), 451.01 (63), 445.21 (36), 443.11 (43), 422.97 (57), 419.14 (95), 408.18 (43), 385.84 (55), 344.15 (57), 211.16 (100), 121.13 (88), 58.93 (75). Anal. Calcd for C_29_H_23_Cl_2_N_7_O (556.45): C, 62.60; H, 4.17; N, 17.62; Found: C, 62.51; H, 4.11; N, 17.65%.

### 3-Methyl-1-phenyl-1,7-dihydro-6H-pyrazolo[3,4-d]pyrimidine-6-thione (7)

A solution of aldehyde **1** (0.01 mol) and thiourea (0.01 mol) in alcoholic sodium hydroxide (20 mL, 20%) was refluxed for 8 h. The reaction mixture was allowed to stand at room temperature and then poured onto ice/water and acidified with diluted hydrochloric acid (10%). The solid obtained was filtered, washed with water, dried, and recrystallized from ethanol to give yellow crystals; mp. 181–183 °C [Lit^56^ mp. 182–184 °C]; yield 65%. IR (KBr, ν, cm^− 1^): 3361 (NH), 3073 (CH aromatic), 2924 (CH aliphatic), 1629 (C = N), 1262 (C = S). ^1^H NMR (400 *MHz*, DMSO-*d*_*6*_) *δ* (ppm): 2.34 (s, 3 H, CH_3_), 7.29 (t, 1 H, Ar-H, *J* = 7.5 *Hz*), 7.45–7.87 (m, 2 H, Ar-H), 8.11 (d, 2 H, Ar-H, *J* = 7.4 *Hz*), 9.57 (s, 1 H, CH pyrimidine), 13.8 (*br*.s, 1 H, NH). EIMS (70 eV, *m/z*, %): 242.45 (M^+^^.^, 7), 222.75 (72), 220.54 (62), 202.05 (49.6), 193.99 (43), 181.67 (54), 176.06 (94), 144.99 (66), 137.08 (59), 131.56 (41), 108.14 (46), 104.17 (73), 101.7 (100), 83.67 (49), 77.99 (57). Anal. Calcd. for C_12_H_10_N_4_S (242.30): C, 59.48; H, 4.16; N, 23.12; Found: C, 59.40; H, 4.12; N, 23.14%.

### 3-(5-Chloro-3-methyl-1-phenyl-1H-pyrazol-4-yl)-2-cyano-N’-((1,3-diphenyl-1H-pyrazol-4-yl)methylene)acrylohydrazide (8)

A solution of aldehyde **1** (0.01 mol) with 2-cyano-*N*’-((1,3-diphenylpyrazol-4-yl)methylene)-acetohydrazide^57^ (0.01 mol) in 1,4-dioxane and piperidine (0.1 mL) was stirred for 8 h at room temperature. The solid product was formed, filtered, and recrystallized from 1,4-dioxane to give yellow crystals; mp. 188–191 °C; yield 63%. IR (KBr, ν, cm^− 1^): 3242 (NH), 3052 (CH aromatic), 2930 (CH aliphatic), 2206 (C ≡ N), 1678 (C = O), 1614 (C = N). ^1^H NMR (400 *MHz*, DMSO-*d*_*6*_) *δ* (ppm): 2.51 (s, 3 H, CH_3_), 7.18–8.14 (m, 15 H, Ar-H), 8.39 (s, 1 H, CH=), 8.69 (s, 1 H, C5-H pyrazole), 9.21 (s, 1 H, CH = N), 11.68 (*br*.s, 1 H, NH). EIMS (70 eV, *m/z*, %): 534.08 (M + 2, 4), 532.01 (M^+^^.^, 13), 505.10 (48), 493.67 (37), 422.44 (47), 413.57 (31), 410.3 (52), 366.44 (32), 354.53 (43), 269.06 (50), 219.84 (37), 208.5 (38), 180.93 (37), 138.34 (49), 112.08 (35), 85.21 (100). Anal. Calcd for C_30_H_22_ClN_7_O (532.00): C, 67.73; H, 4.17; N, 18.43; Found: C, 67.60; H, 4.10; N, 18.46%.

### 5-((5-Chloro-3-methyl-1-phenyl-1H-pyrazol-4-yl)methylene)-3-phenylthiazolidine-2,4-dione (9)

A mixture of aldehyde **1** (0.01 mol) and 3-phenylthiazolidine-2,4-dione (0.01 mol) in glacial acetic acid and anhydrous sodium acetate (0.01 mol) was refluxed for 10 h. The formed precipitate was filtered and recrystallized from ethanol to give brown crystals; mp. 160–162 °C; yield 58%. IR (KBr, ν, cm^− 1^): 3140, 3062 (CH aromatic), 2987, 2854 (CH aliphatic), 1720, 1677 (C = O vibrational coupling). ^1^H NMR (400 *MHz*, DMSO-*d*_*6*_) *δ* (ppm): 2.45 (s, 3 H, CH_3_), 7.54–7.62 (m, 10 H, Ar-H), 9.91 (s, 1 H, CH=). EIMS (70 eV, *m/z*, %): 397.31 (M + 2, 4), 395.45 (M^+^^.^, 11), 378.32 (20), 377.23 (22), 336.23 (48), 159.13 (30), 157.55 (22), 131.29 (21), 93.07 (100), 92.16 (27), 78.05 (32), 77.15 (55), 76.29 (21), 66.13 (33), 65.26 (25), 63.29 (21). Anal. Calcd. for C_20_H_14_ClN_3_O_2_S (395.86): C, 60.68; H, 3.56; N, 10.62; Found: C, 60.57; H, 3.49; N, 10.59%.

### 3-(3-(5-Chloro-3-methyl-1-phenyl-1H-pyrazol-4-yl)acryloyl)-2H-chromen-2-one (10)

A mixture of aldehyde **1** (0.01 mol) and 3-acetylchromen-2-one (0.01 mol) in absolute ethanol (20 mL) containing piperidine (0.1 mL) was refluxed for 3 h. The formed precipitate was filtered and recrystallized from butanol to get yellow crystals; mp. 260–262 ^o^C; yield 43%. IR (KBr, ν, cm^− 1^): 3064 (CH aromatic), 2977, 2958 (CH aliphatic), 1721 (C = O coumarin), 1656 (C = O ketone). ^1^H NMR (400 *MHz*, DMSO-*d*_*6*_) *δ* (ppm): 2.54 (s, 3 H, CH_3_), 7.45–7.62 (m, 11 H, Ar-H + CH = CH), 8.59 (s, 1 H, C4-H coumarin). EIMS (70 eV, *m/z*, %): 392.80 (M + 2, 8), 390.41 (M^+^^.^, 24), 367.45 (55), 313.13 (28), 252.17 (33), 227.21 (35), 185.10 (45), 157.83 (34), 123.40 (81), 107.05 (64), 97.85 (100), 95.08 (98). Anal. Calcd for C_22_H_15_ClN_2_O_3_ (390.82): C, 67.61; H, 3.87; N, 7.17; Found: C, 67.53; H, 3.80; N, 7.20%.

### 3-(5-Chloro-3-methyl-1-phenyl-1H-pyrazol-4-yl)-1-(thiophen-2-yl)prop-2-en-1-one (11)

A mixture of aldehyde **1** (0.01 mol) and 2-acetylthiophene (0.01 mol) in ethanolic sodium hydroxide (10 mL, 20%) was stirred for 3 h at an ambient temperature. The formed precipitate was filtered and recrystallized from ethyl alcohol to furnish yellow crystals, mp. > 300 °C, yield 56%. IR (KBr, ν, cm^−1^): 3065 (CH aromatic), 2983, 2922 (CH aliphatic), 1649 (C = O). ^1^H NMR (400 *MHz*, DMSO-*d*_*6*_) *δ* (ppm): 2.53 (s, 3 H, CH_3_), 7.32 (t, 1 H, CH, *J* = 7.4 *Hz*), 7.50–7.56 (m, 2 H, Ar-H), 7.59–7.63 (m, 5 H, Ar-H), 8.08 (d, 1 H, CH=, *J* = 6.5 *Hz*), 8.17 (d, 1 H. CH=, *J* = 6.5 *Hz*). EIMS (70 eV, *m/z*, %): 330.20 (M + 2, 20), 328.48 (M^+^^.^, 51), 296.54 (30), 242.35 (45), 205.51 (100), 195.02 (48), 88.99 (58), 85.48 (73). Anal. Calcd for C_17_H_13_ClN_2_OS (328.81): C, 62.10; H, 3.99; N, 8.52; Found: C, 62.01; H, 3.93; N, 8.54%.

### Antiviral activity

#### Materials

NDV Genotype 7D antigen accession No. KM288609 of 10^10^ EID50/mL was obtained from Central Laboratory for Evaluation of veterinary Biologics (CLEVB), Cairo, Egypt. Eight compounds were checked (**2**, **3**, **4**, **6**, **7**, **9**, **10**, and **11**). Embryonated SPF chicken eggs (ECES) one day old SPF come from the National Project for production of SPF eggs, Kom Oshim, Fayoum, Egypt. It was kept in the egg incubator at 37 °C with humidity 60–80% till the age of 9–11 days old. Freshly collected chicken erythrocytes (1 and 10%) were prepared in saline solution after several washes in HA assay.

### Methods

#### Cytotoxicity

Groups of nine days SPF embryonated chicken eggs were inoculated with different concentrations of each tested compound for the calculation of cytotoxicity concentration 50% (CC_50_). Uninoculated SPF eggs were always included as control of embryo. The eggs were inoculated *via* allantoic cavity and were incubated for six days post inoculation at 37 °C with humidity 70%. CC_50_ of each test compound was determined as the concentration of compound that induced any embryos mortalities or any deviation than normal control embryos in 50% of embryonated chicken eggs.

#### Inhibition concentration

Other groups of SPF embryonated chicken eggs were inoculated with a mixture of minimal cytotoxic concentration of different tested compounds with 10^10^ EID_50_/mL of NDV (0.2 mL per egg) for calculation of the antiviral inhibitory concentration 50% (IC_50_). Uninoculated SPF eggs were always included as control of embryo. The eggs were inoculated *via* allantoic cavity and were incubated for six days PI at 37 °C with humidity 70%. IC_50_ of tested compounds was assayed as the concentration of the compound that fully inhibited virus effect in 50% of embryonated chicken eggs.

#### CC_50_/IC_50_ assay

Pharmaceutical safety is an essential factor in the development of every medicament. It is essential to establish that an investigational product has antiviral activity at concentrations that can be achieved in vivo without inducing toxic effects to cells. Furthermore, in a cell culture model, the apparent antiviral activity of an investigational product can be the result of host cell death after exposure to the product. Thus, it is crucial to determine the cytotoxic potential of the formulation on the cell line used in the antiviral assays.

CC_50_ is the concentration of test compounds required to reduce cell viability by 50%. The cytotoxicity of the test compounds is best determined concurrently with uninfected cells to obtain CC_50_ values. Cytotoxicity tests use a series of increasing concentrations of the antiviral product to determine what concentration results in the death of 50% of the host cells. This value is the median cellular cytotoxicity concentration and is identified by CC_50_.

We recommend determining CC_50_ values in both stationary and dividing cells from multiple relevant human cell types and tissues to ascertain the potential for cell-cycle, species, or tissue-specific toxicities. In-vitro susceptibility of viruses to antiviral agents is typically measured as IC_50_, which is the concentration of antiviral that lowers 50% of the virus-induced cytopathic effect (CPE) and the number of plaques formed. The relative effectiveness of the investigational product in inhibiting viral replication compared to inducing cell death is defined as the therapeutic or selectivity index.

### Selectivity index (SI)

SI of the tested compound was expressed as CC_50_/IC_50_ was calculated using the method described^61^.

### Antiviral assay

Screening of antiviral activity of different compounds was performed using their non-cytotoxic concentration. Therefore, the investigation of different compounds as inhibitory agents against NDV replication (12-fold serial dilutions were performed) in SPF chicken embryos and their cytotoxicity was recorded. Nontoxic concentrations of tested compounds (lower than CC_50_) were checked for antiviral properties against NDV replication in SPF chicken embryos as follows:

(1) Mixing 0.2 mL of the NDV and 0.2 mL of each compound separately and incubation for 1 h at an ambient temperature.

(2) 12-fold serial dilutions of the mixtures were prepared.

(3) Eight groups (480 eggs) of 9 days old SPF were inoculated by varying serial dilutions (from 1st dilution to 12th dilution, each dilution in five eggs) of each mixture (0.2 mL per egg) were inoculated *via* allantoic route.

(4) Daily examination of the inoculated eggs, deaths through 24 h post inoculation (PI) were discarded, and mortality between 2nd day and 6th day recognized as specific death.

(5) The NDV infectivity in ECE was determined by haemagglutinating activity of the allantoic fluid of the inoculating eggs as measured by the micro technique of the haemagglutinating (HA) test^62^.

(6) Virus titer was calculated using method^62^ and comparison of the known NDV titer with the antiviral activity.

### Immune response of different compounds in SPF chicks

Evaluation of immune boosting properties of different compounds (**4**, **6**, **7**, **9**, and **11**) was examined in one day old SPF chicks. A total of 160 SPF chicks were divided into eight groups each contains 20 chicks (each group isolated in biosafety isolator). At seven days old, samples from each group were assessed for ND antibodies by HI test to detect maternal antibodies. All chicks were proved to be free from ND antibodies by HI test.

First group (vaccinated NDV ‘Lasota’ only): consist of 20 experimental chicks were vaccinated with inactivated NDV vaccine. The vaccine was injected with 0.5 mL/bird subcutaneously in the dorsal region of the neck, each group consists of 20 chicks were vaccinated with inactivated NDV vaccine. Six milliliters from diluted nontoxic concentration of each tested compound (**4**, **6**, **7**, **9**, and **11**), respectively (each group separated in biosafety isolator) was added to the drinking water for five groups and another three groups every day for 28 day post vaccination. Eighth group (20 chicks) was kept in separate isolator as non-vaccinated negative control.

Blood samples were collected separately from groups from jugular vein for estimation of the HI antibody titer in serum of vaccinated chicks at 28-day post vaccination by HA inhibition technique described^68^ using standard ND antigen (for HA) with the comparison of humeral response of vaccinated group and other vaccinated groups mixed with the tested compound as immunostimulant. After collection of blood samples vaccinated groups and control group were challenged with a local isolated strain of NDV (Genotype 7D NDV antigen accession No. KM288609) containing at least 10^10^ EID_50_/bird^69^.

## Electronic supplementary material

Below is the link to the electronic supplementary material.

Supplementary Material 1

Supplementary Material 2

Supplementary Material 3

## Data Availability

All data generated or analyzed during this study are included in this published article and its supplementary information files.
