# Supplementary material for: Antiviral activity of newly synthesized pyrazole derivatives against Newcastle disease virus
Source: Sci Rep. 2025 May 28;15:18745. doi: 10.1038/s41598-025-03495-6 (PMC12119990; doi:10.1038/s41598-025-03495-6)
Supplement: Supplementary file 1 — Supplementary Material 1 [file 41598_2025_3495_MOESM1_ESM.docx]

**Antiviral Activity of Newly Synthesized Pyrazole Derivatives Against Newcastle Disease Virus**

**Ahmed El-Sewedy ^1^, Eman A. El-Bordany ^1^, Naglaa F. H. Mahmoud ^1^, Alaa R. I. Morsy ^2^, Safwa Z. Mohamed ^2^, Sayed K. Ramadan ^1,*^**

^1^ Chemistry Department, Faculty of Science, Ain Shams University, Cairo, 11566, Egypt

^2^ Central Laboratory for Evaluation of Veterinary Biologics (CLEVB), Agricultural Research Center, Egypt

**E*-mail: [sayed.karam2008@sci.asu.edu.eg](mailto:sayed.karam2008@sci.asu.edu.eg)

**Supporting information:**

**Table S1**. Parameters of the possible isomers of compounds **8-11**.

| **Compds**. | **Isomers** | **E (kcal/mol)*** | **Dipole/dipole** | **1,4-VDW**** |
| --- | --- | --- | --- | --- |
| **8** |   *Z*-isomer | **54.0066** | -4.3982 | 29.1534 |
|  |   *E*-isomer | 59.4392 | -3.4251 | 28.8871 |
| **9** |   *Z*-isomer | 64.1975 | -0.5270 | 21.4147 |
|  |   *E*-isomer | **60.9977** | -1.9649 | 21.8746 |
| **10** |   *Z*-isomer | 68.9568 | 4.0585 | 26.2842 |
|  |   *E*-isomer | **59.5791** | 5.3560 | 26.4217 |
| **11** |   *Z*-isomer | 866.6631 | NA*** | 19.1106 |
|  |   *E*-isomer | **53.7592** | 1.6899 | 15.2078 |

* E: Total energy of the structure.

** VDW: van-der Waal interaction

*** NA: Not computed due to high VDW interactions


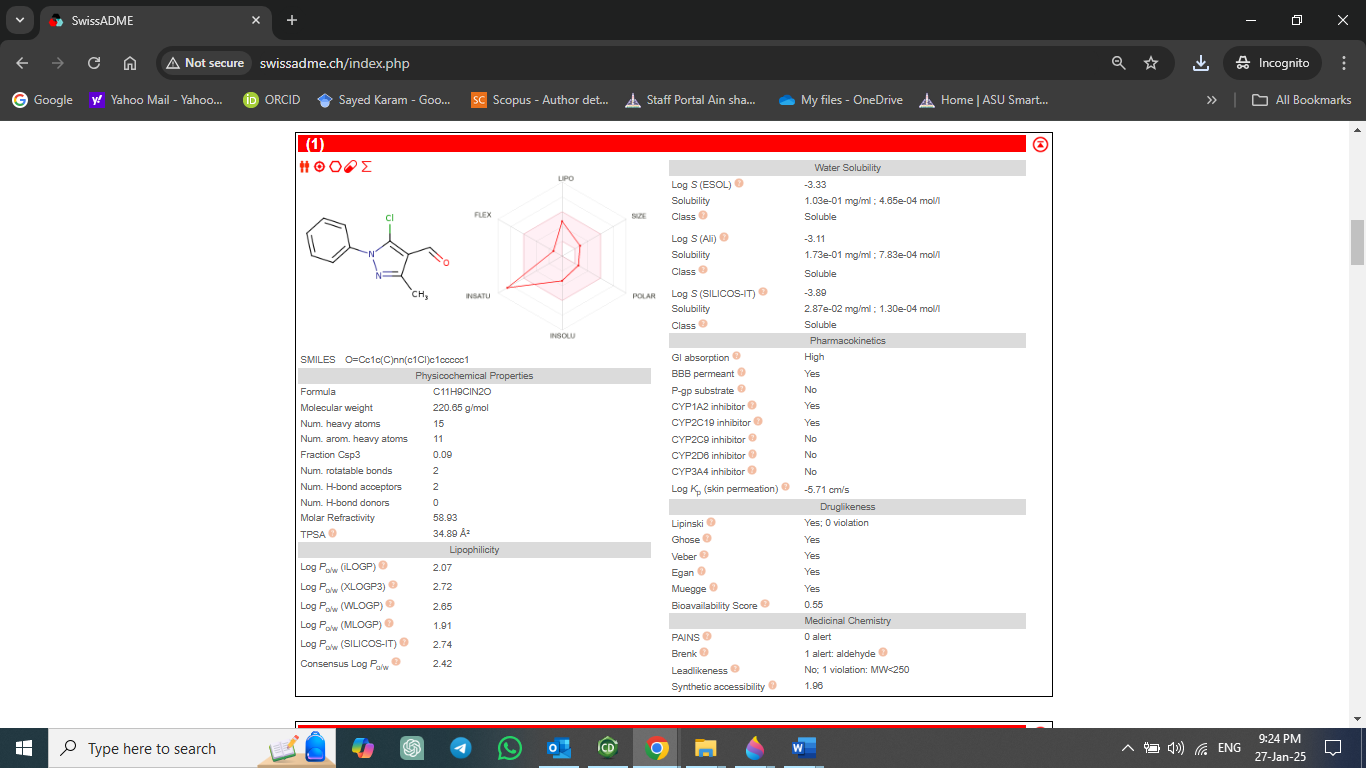


**Fig. S1**. ADME profile of compound **1**.


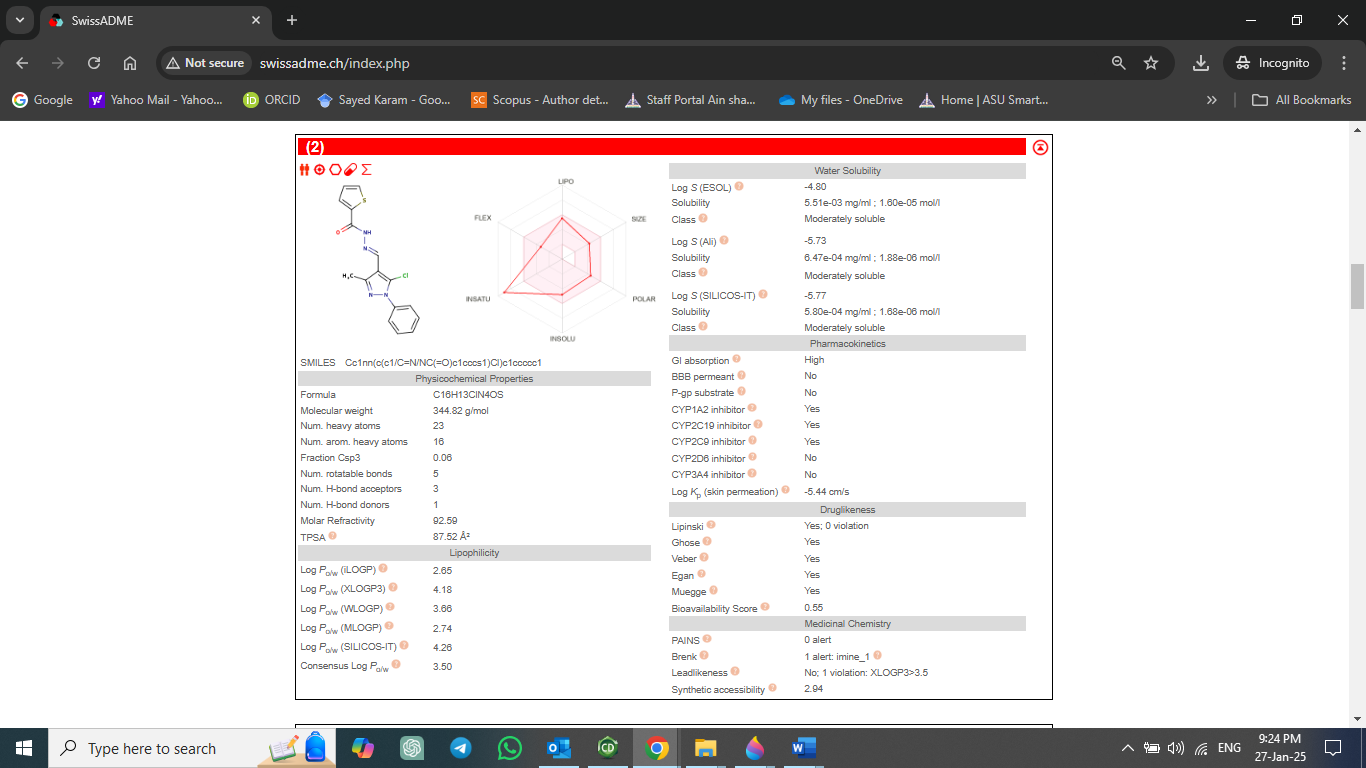


**Fig. S2**. ADME profile of compound **2**.


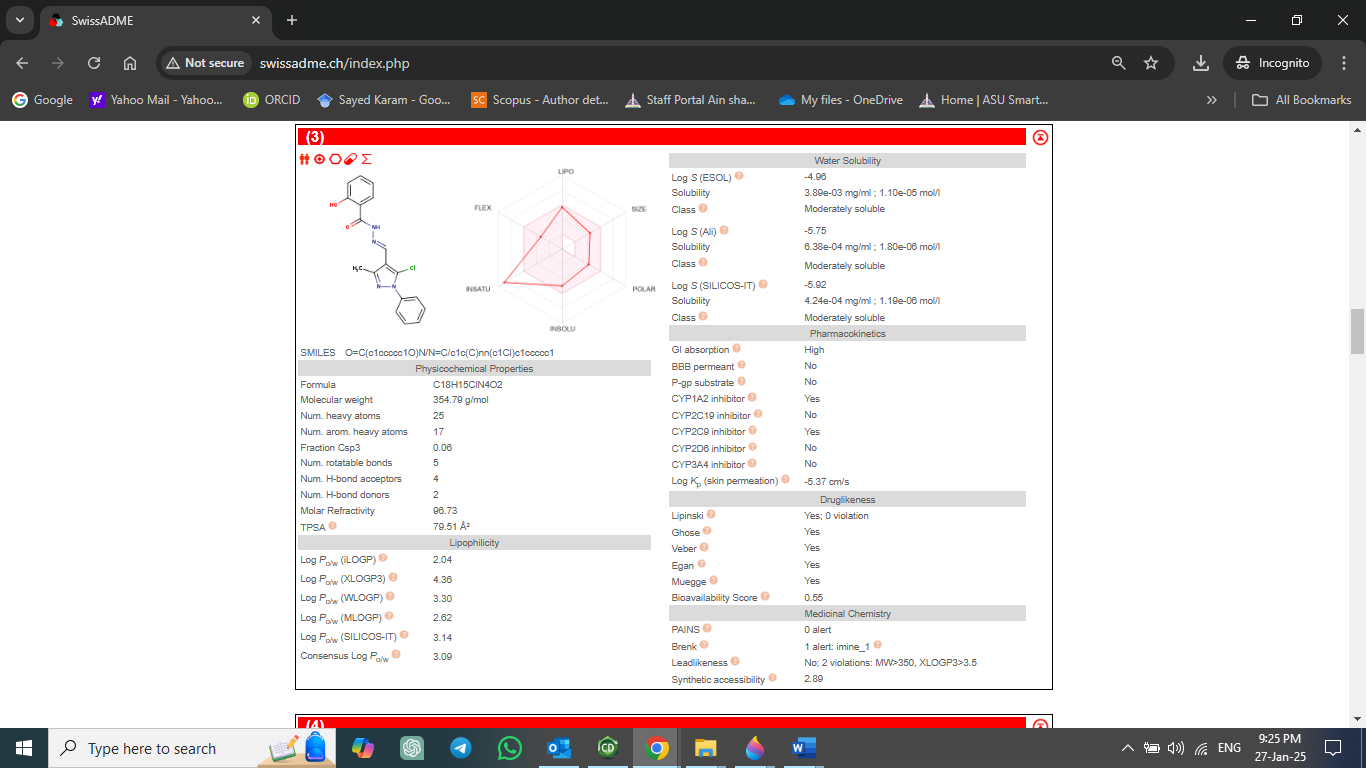


**Fig. S3**. ADME profile of compound **3**.


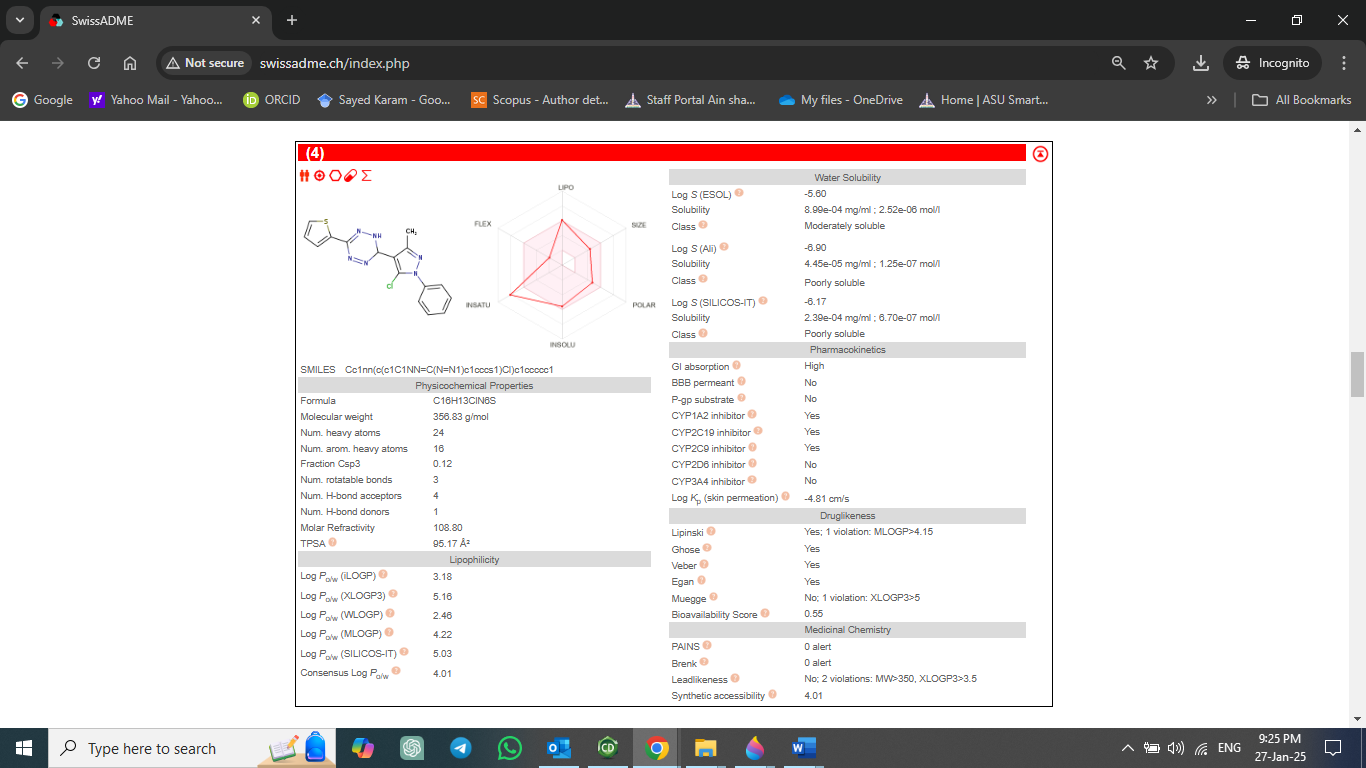


**Fig. S4**. ADME profile of compound **4**.


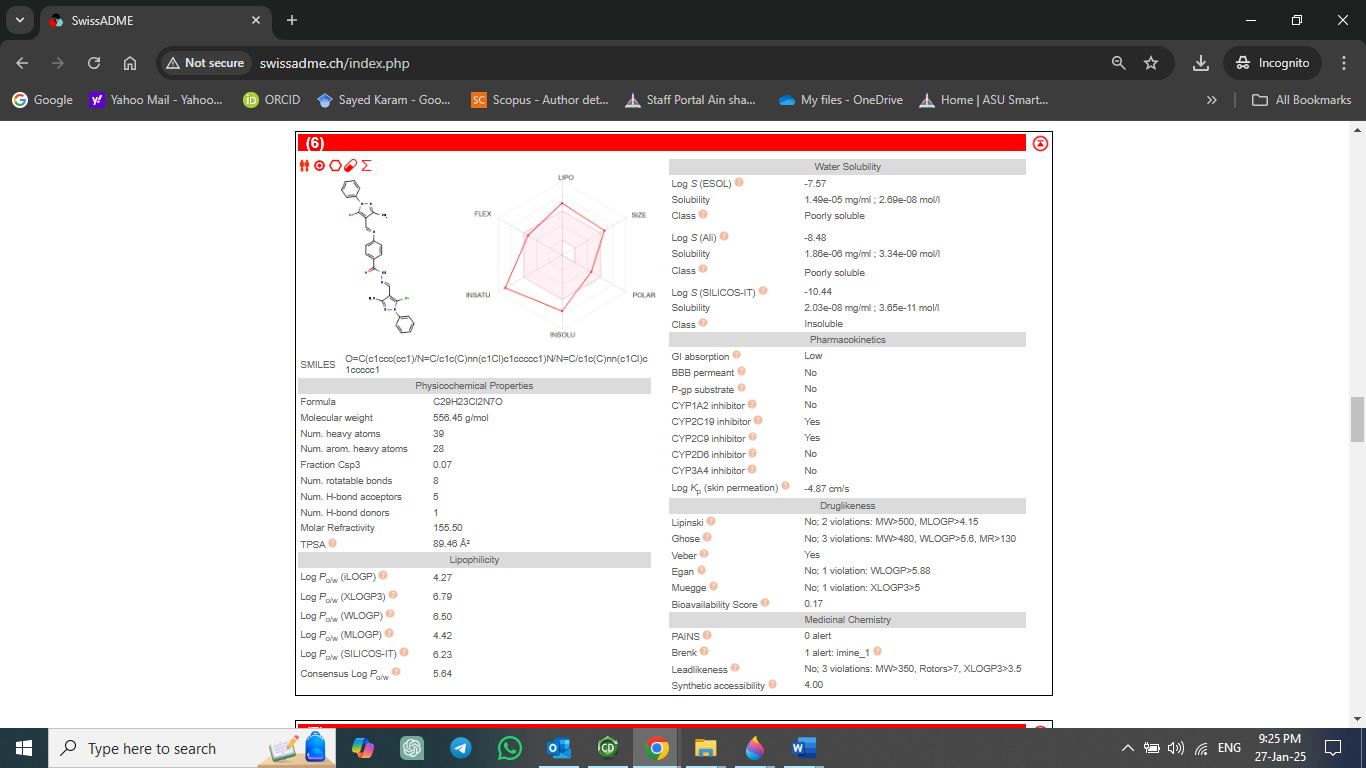


**Fig. S5**. ADME profile of compound **6**.


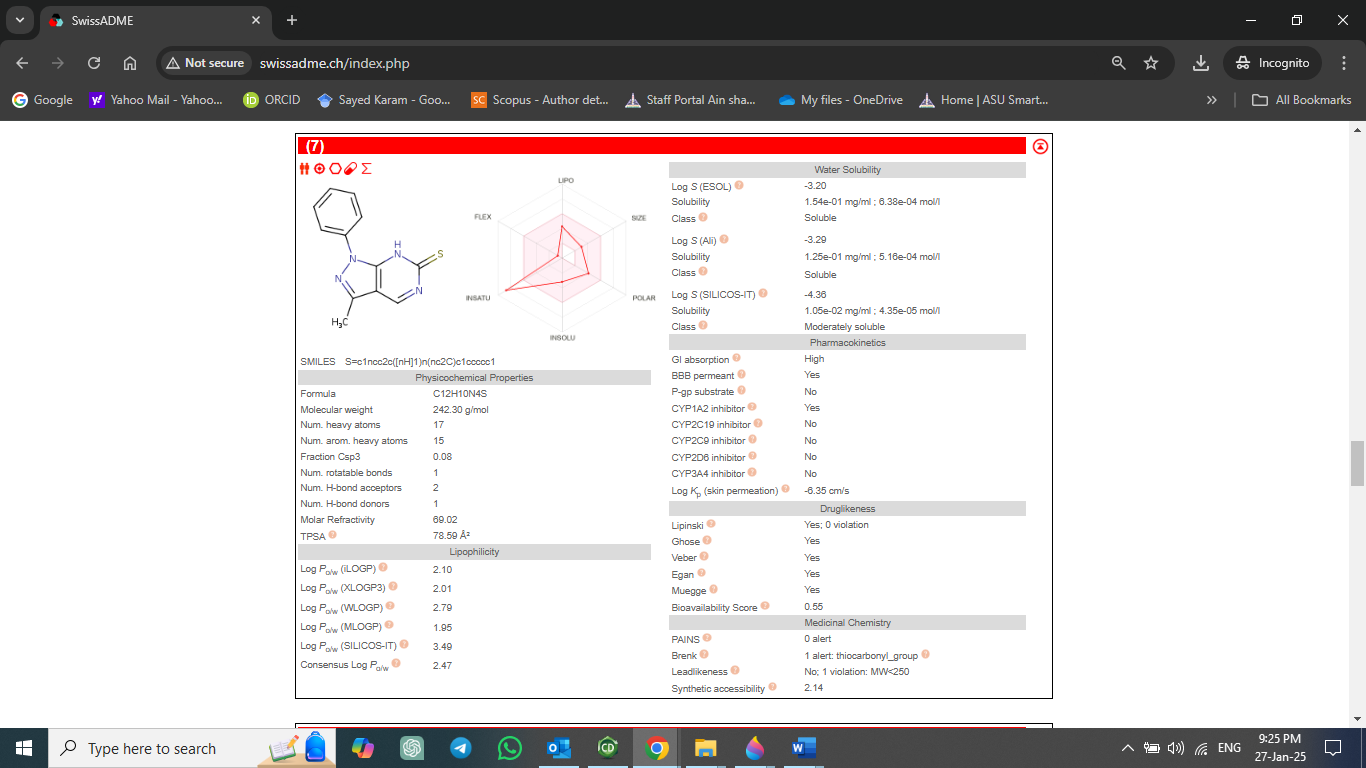


**Fig. S6**. ADME profile of compound **7**.


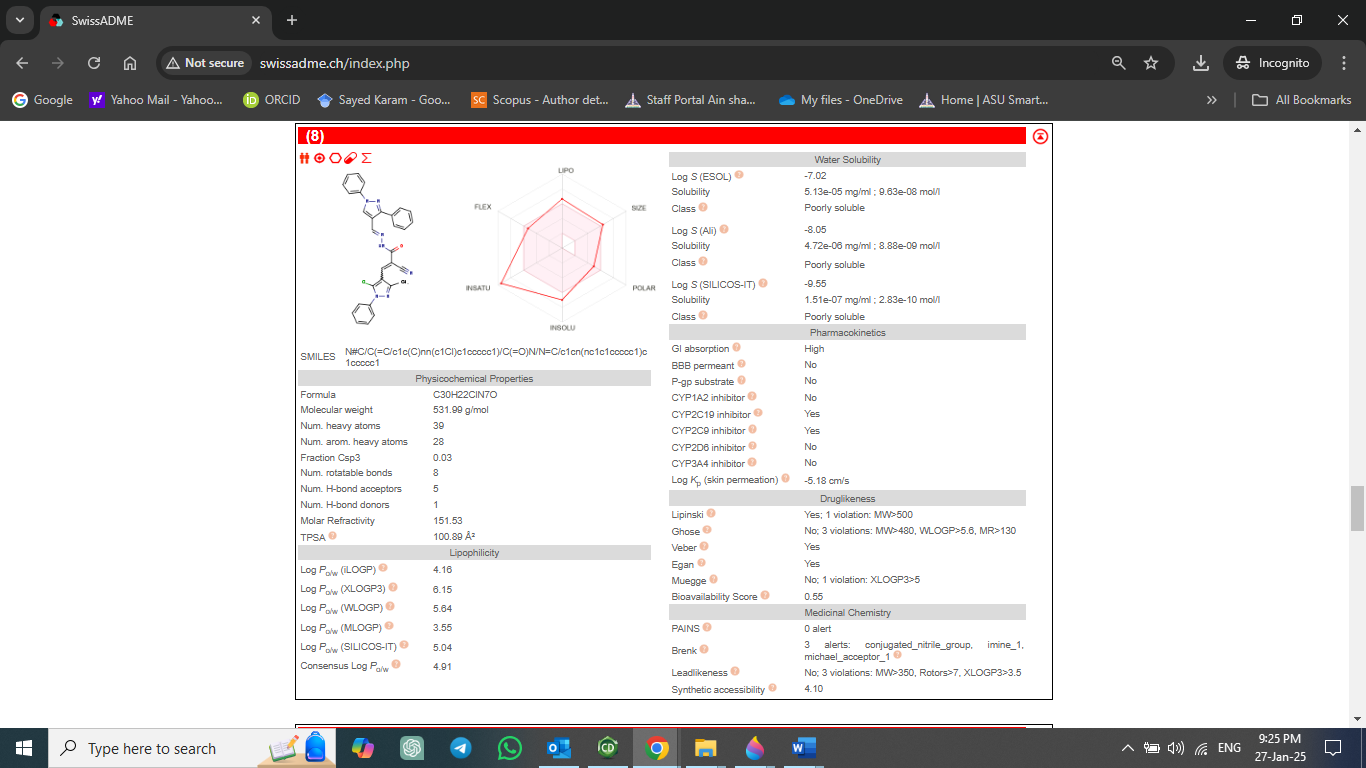


**Fig. S7**. ADME profile of compound **8**.


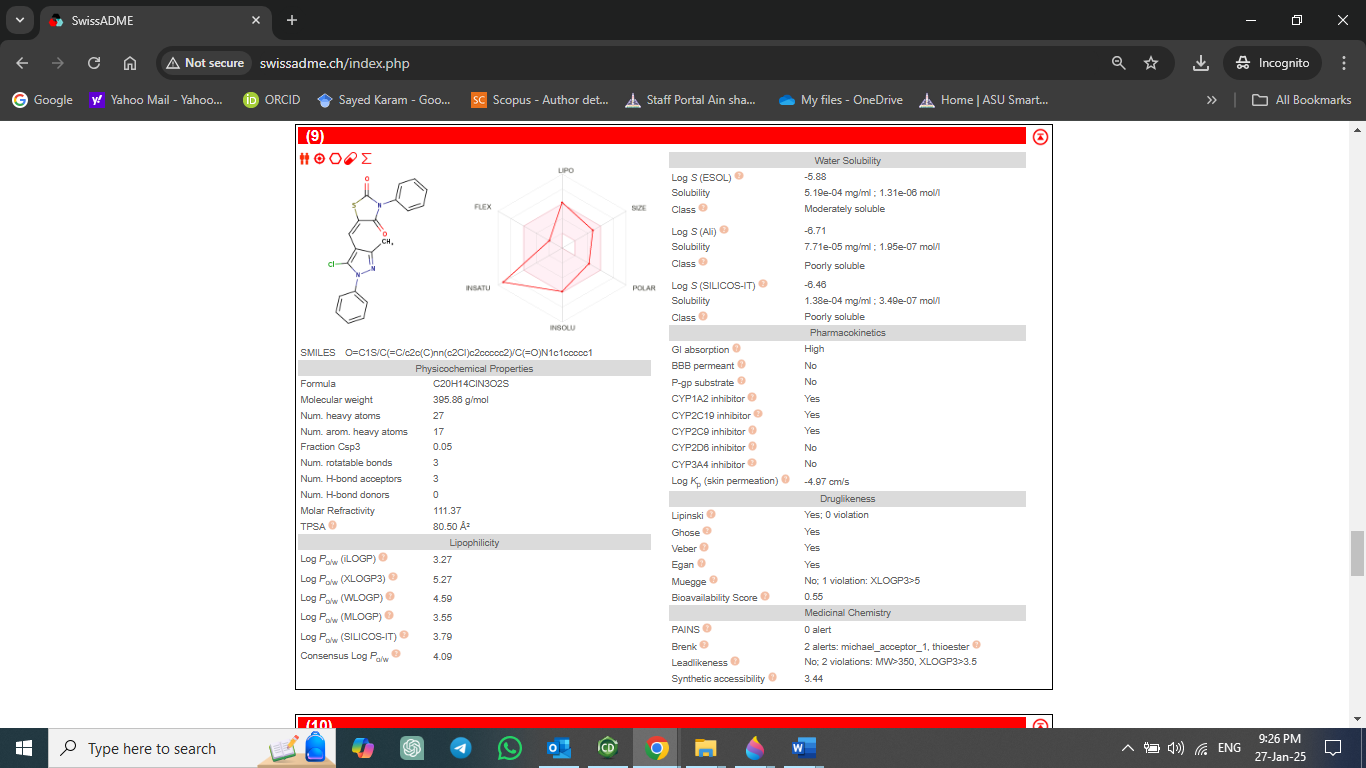


**Fig. S8**. ADME profile of compound **9**.


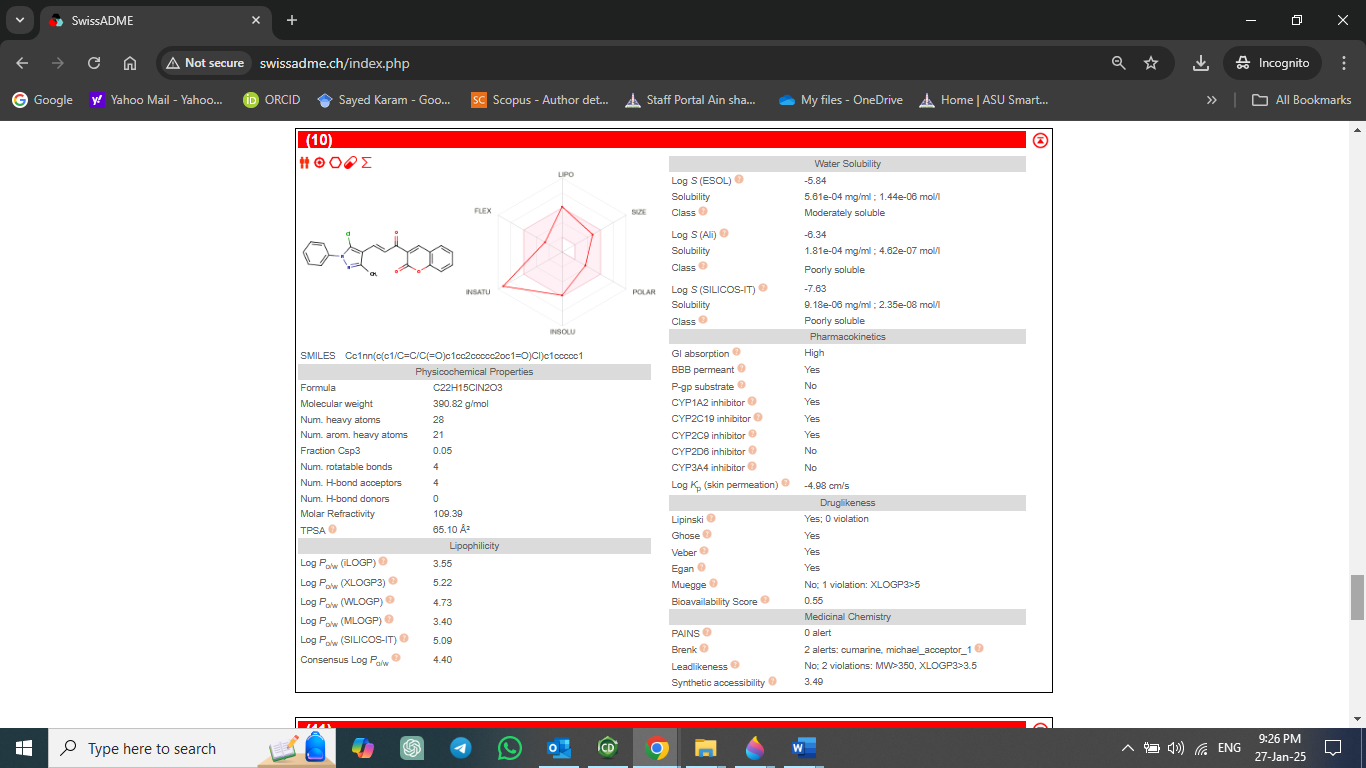


**Fig. S9**. ADME profile of compound **10**.


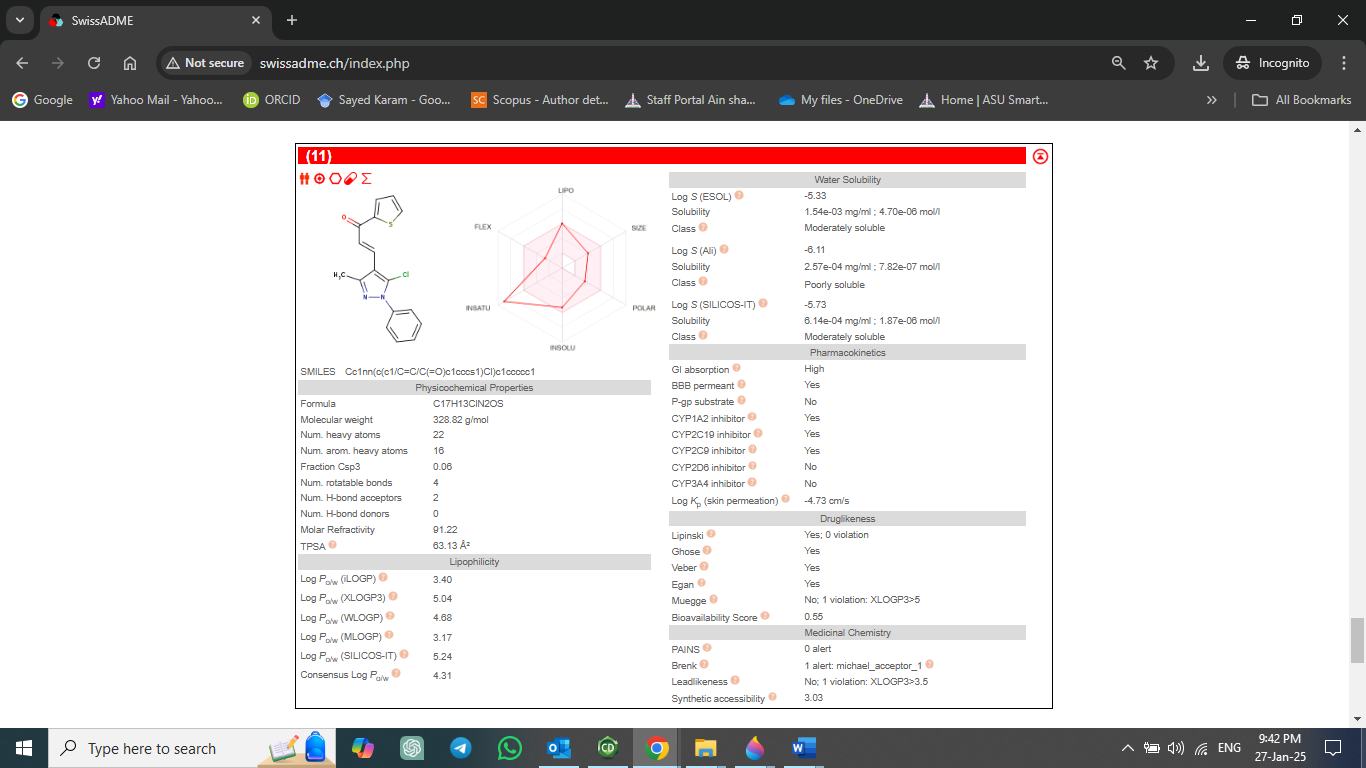


**Fig. S10**. ADME profile of compound **11**.


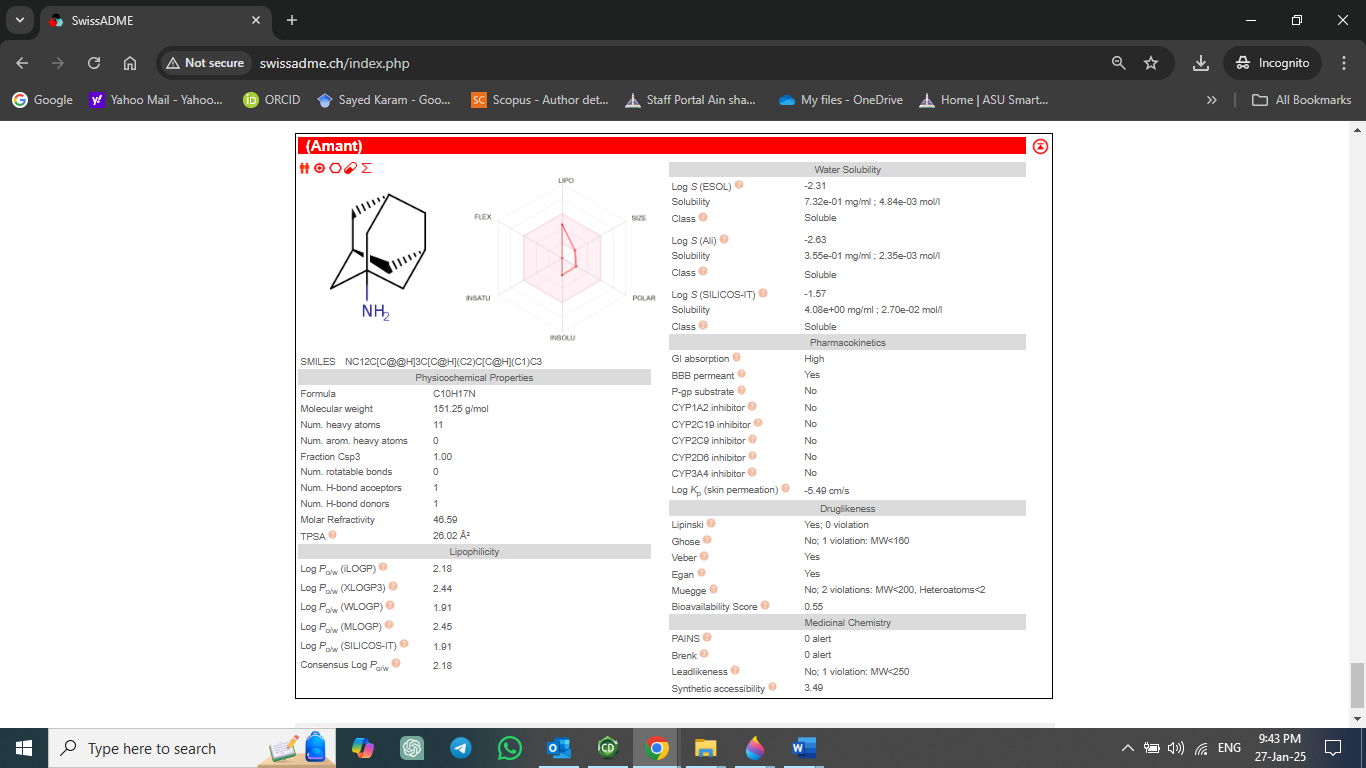


**Fig. S11**. ADME profile of Amantadine.
