# Supplementary material for: Antiviral activity of newly synthesized pyrazole derivatives against Newcastle disease virus
Source: Sci Rep. 2025 May 28;15:18745. doi: 10.1038/s41598-025-03495-6 (PMC12119990; doi:10.1038/s41598-025-03495-6)
Supplement: Supplementary file 2 — Supplementary Material 2 [file 41598_2025_3495_MOESM2_ESM.pdf]

Antiviral Activity of Newly Synthesized Pyrazole Derivatives Against Newcastle Disease Virus

Ahmed El-Sewedy [1], Alaa R. I. Morsy [2], Eman A. El-Bordany [1], Naglaa F. H. Mahmoud[1], Safwa Z. Mohamed [2], Sayed K. Ramadan [1,*]

[1] Chemistry Department, Faculty of Science, Ain Shams University, Cairo, 11566, Egypt

[2] Central Laboratory for Evaluation of Veterinary Biologics (CLEVB), Agricultural Research Center, Cairo, Egypt

*E-mail: sayed.karam2008@sci.asu.edu.eg

Spectral data:

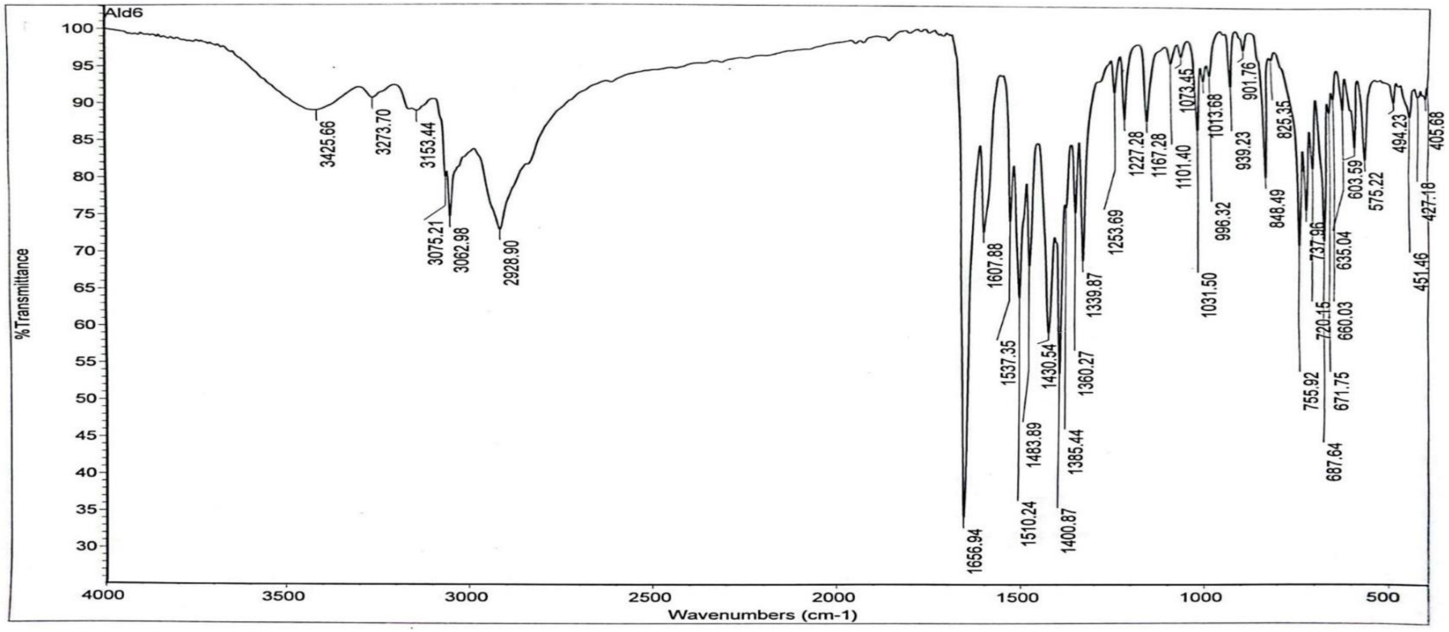

Fig. S12. IR spectrum of compound **2**

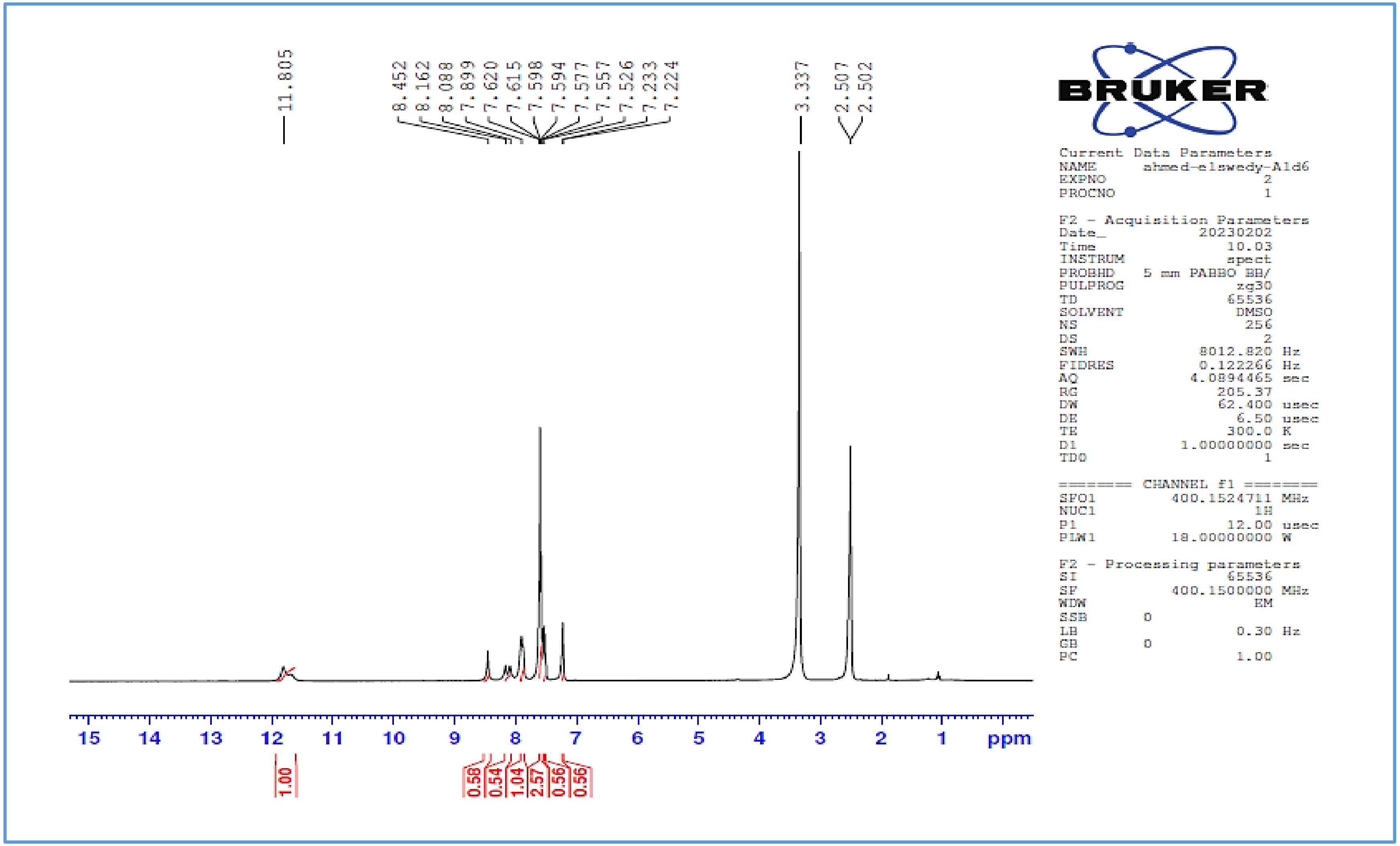

Fig. S13. ^{1}H NMR spectrum (DMSO-d_6) of compound **2**

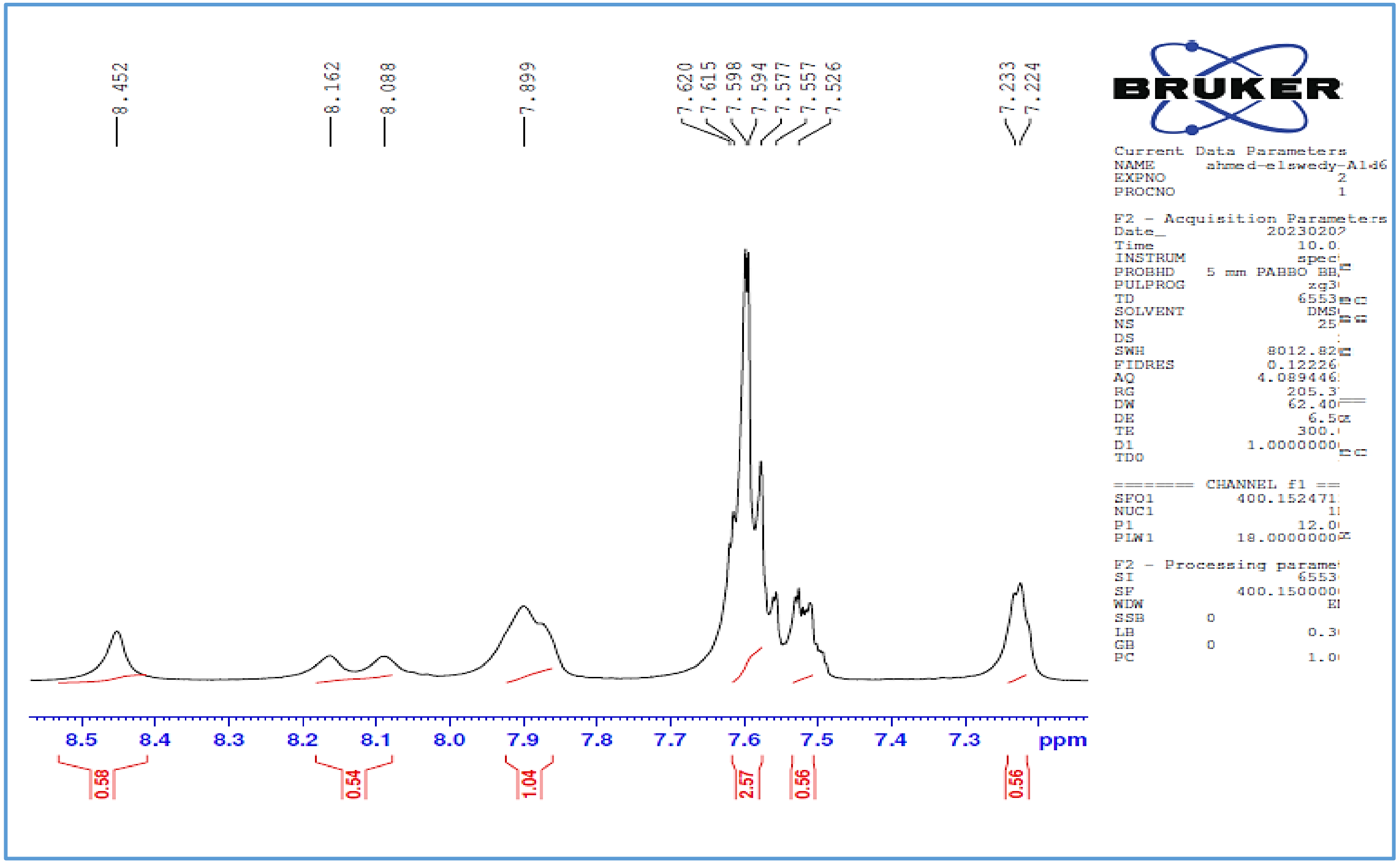

Fig. S14. Cont. ^{1}H NMR spectrum (DMSO-d_6) of compound **2**

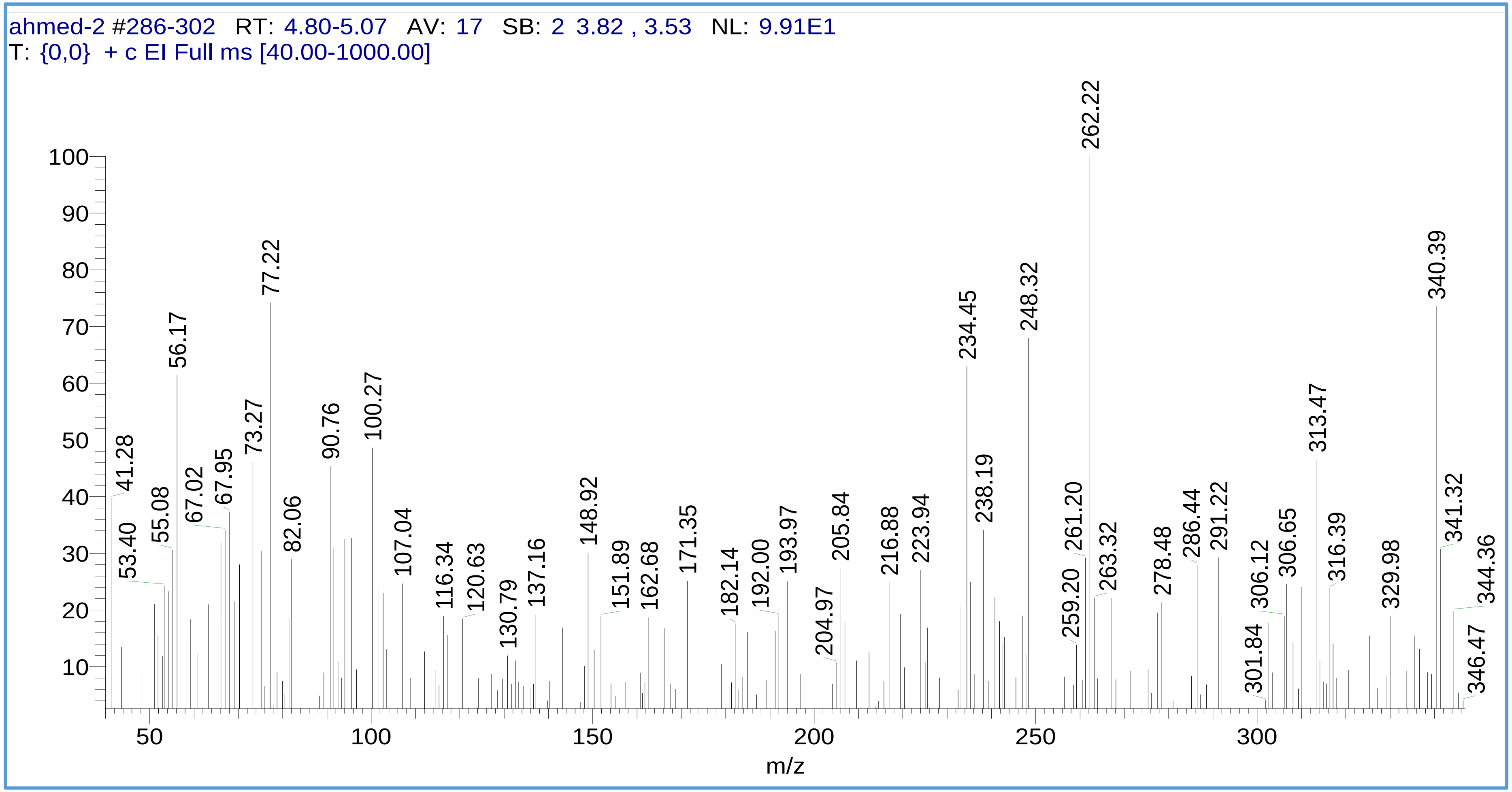

Fig. S15. EI-Mass spectrum of compound **2**

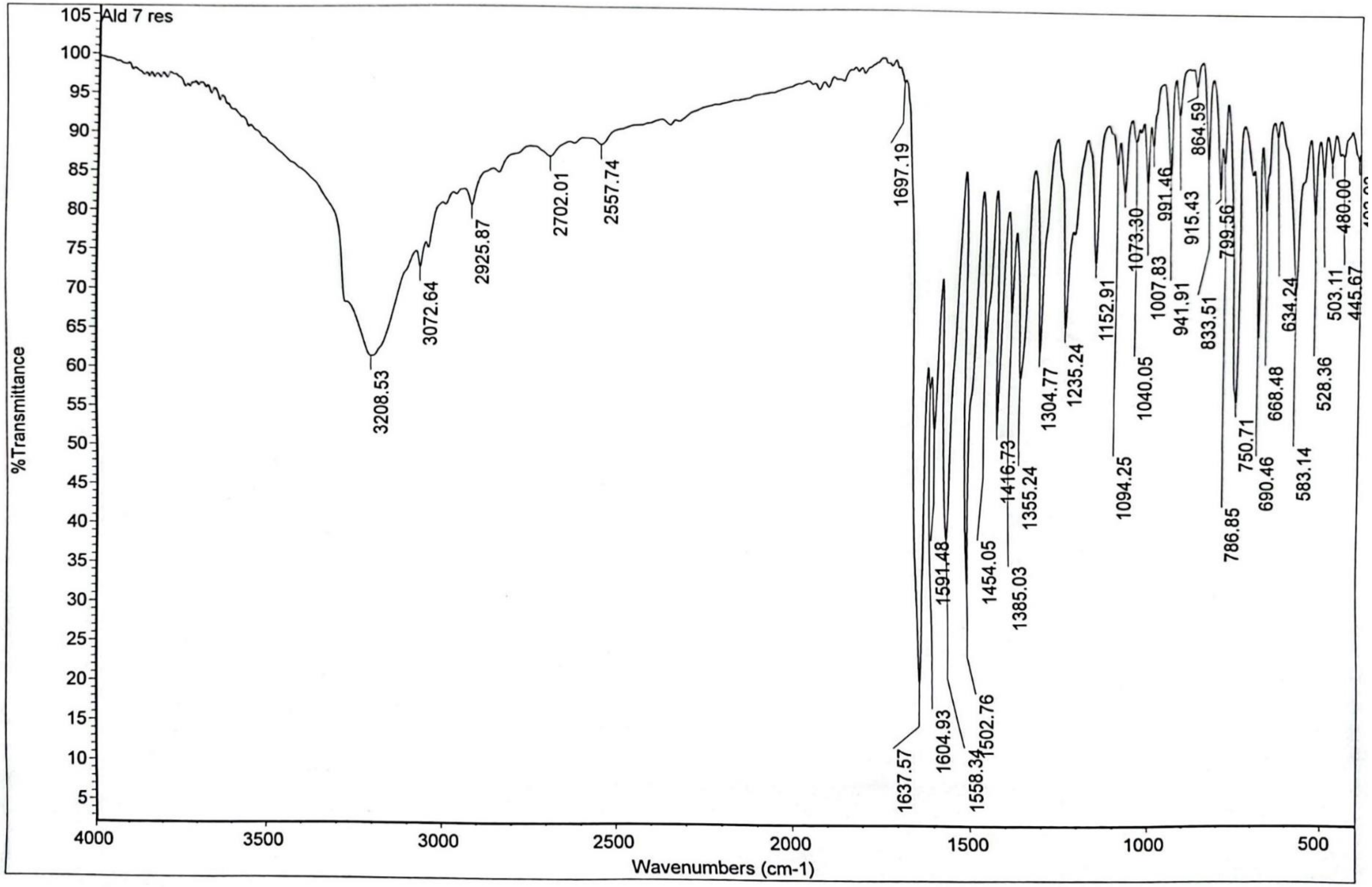

Fig. S16. IR spectrum of compound **3**

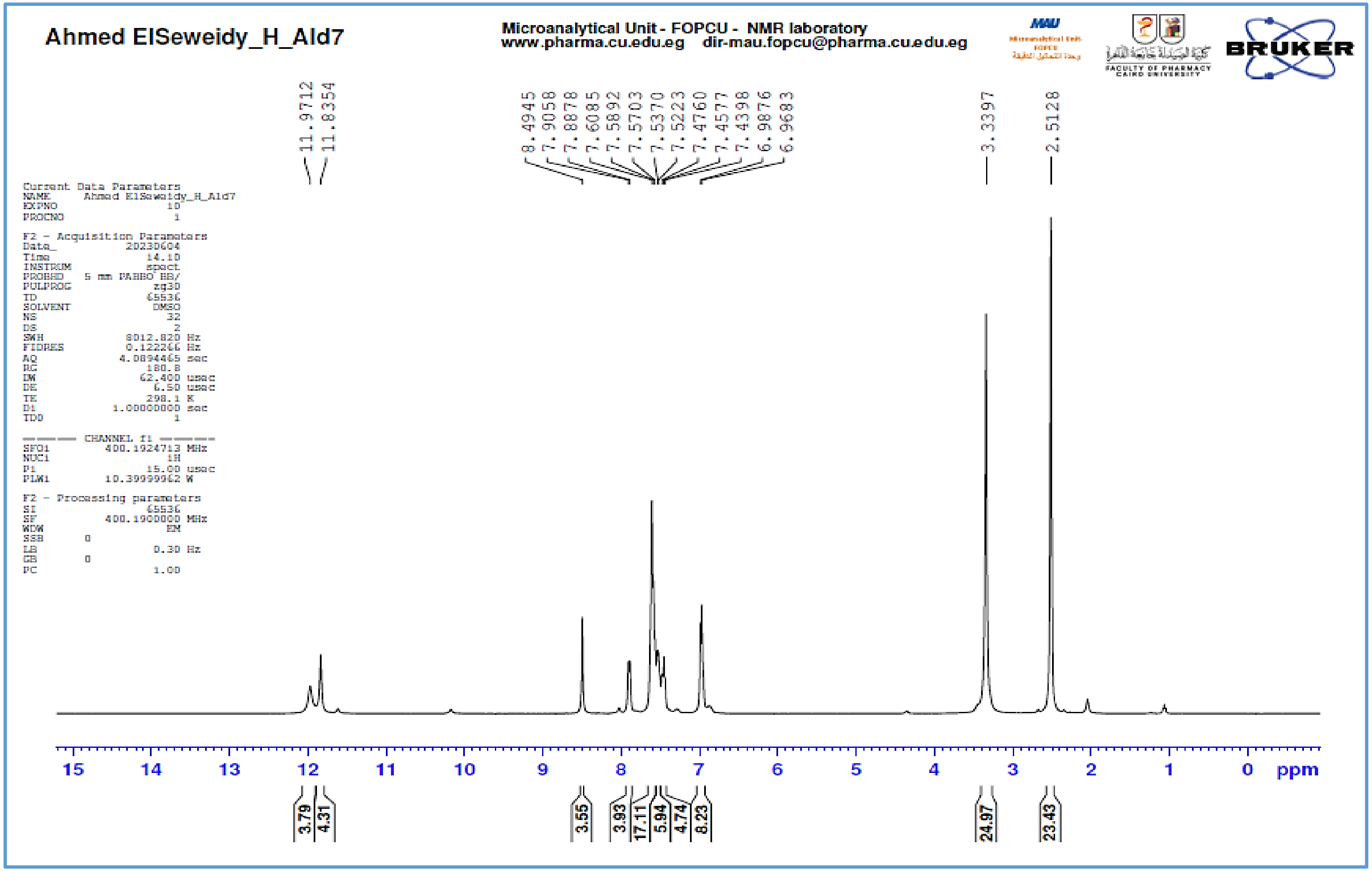

Fig. S17. ^{1}H NMR spectrum (DMSO-d_6) of compound **3**

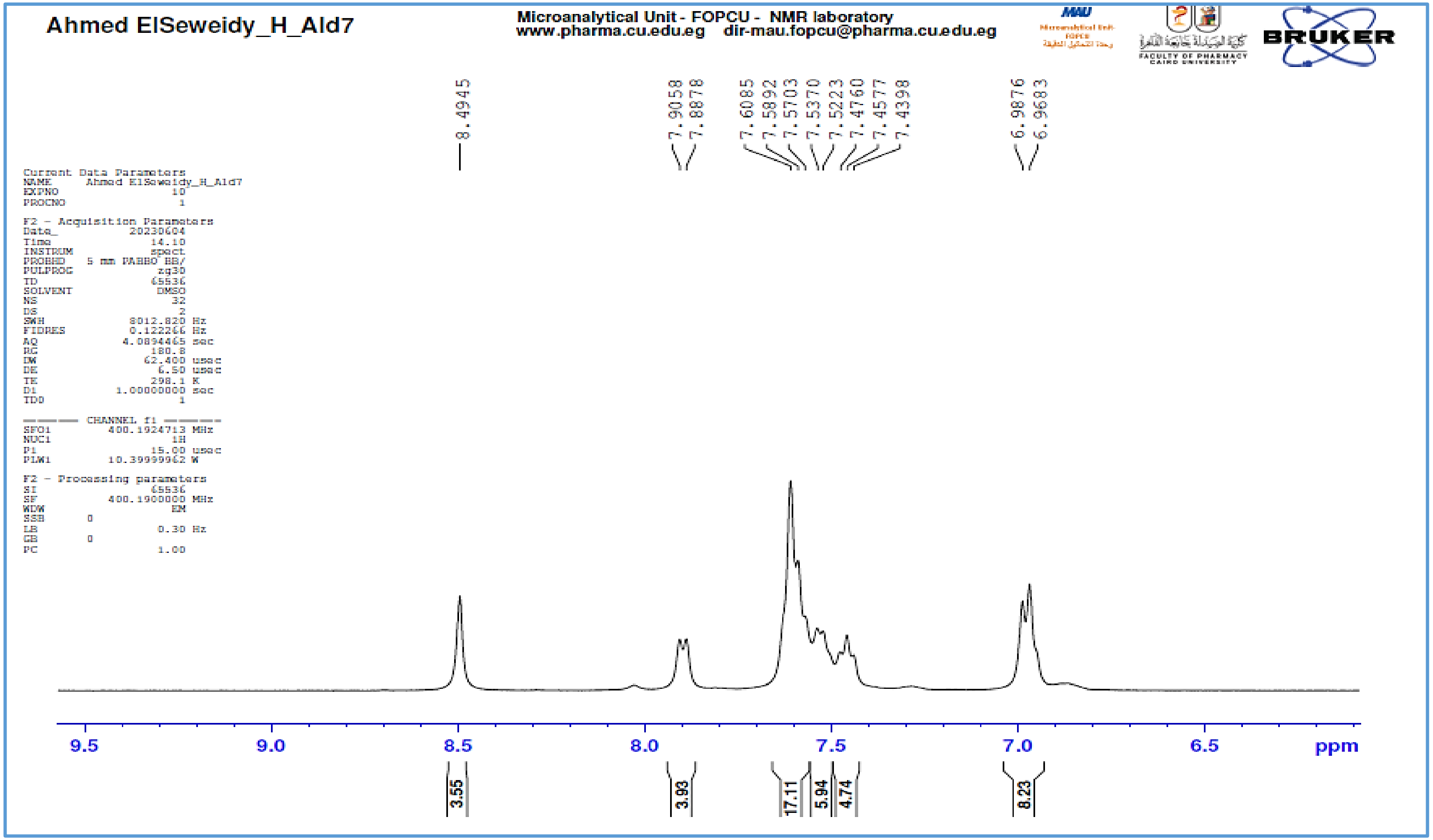

Fig. S18. Cont. ^{1}H NMR spectrum (DMSO-d_6) of compound **3**

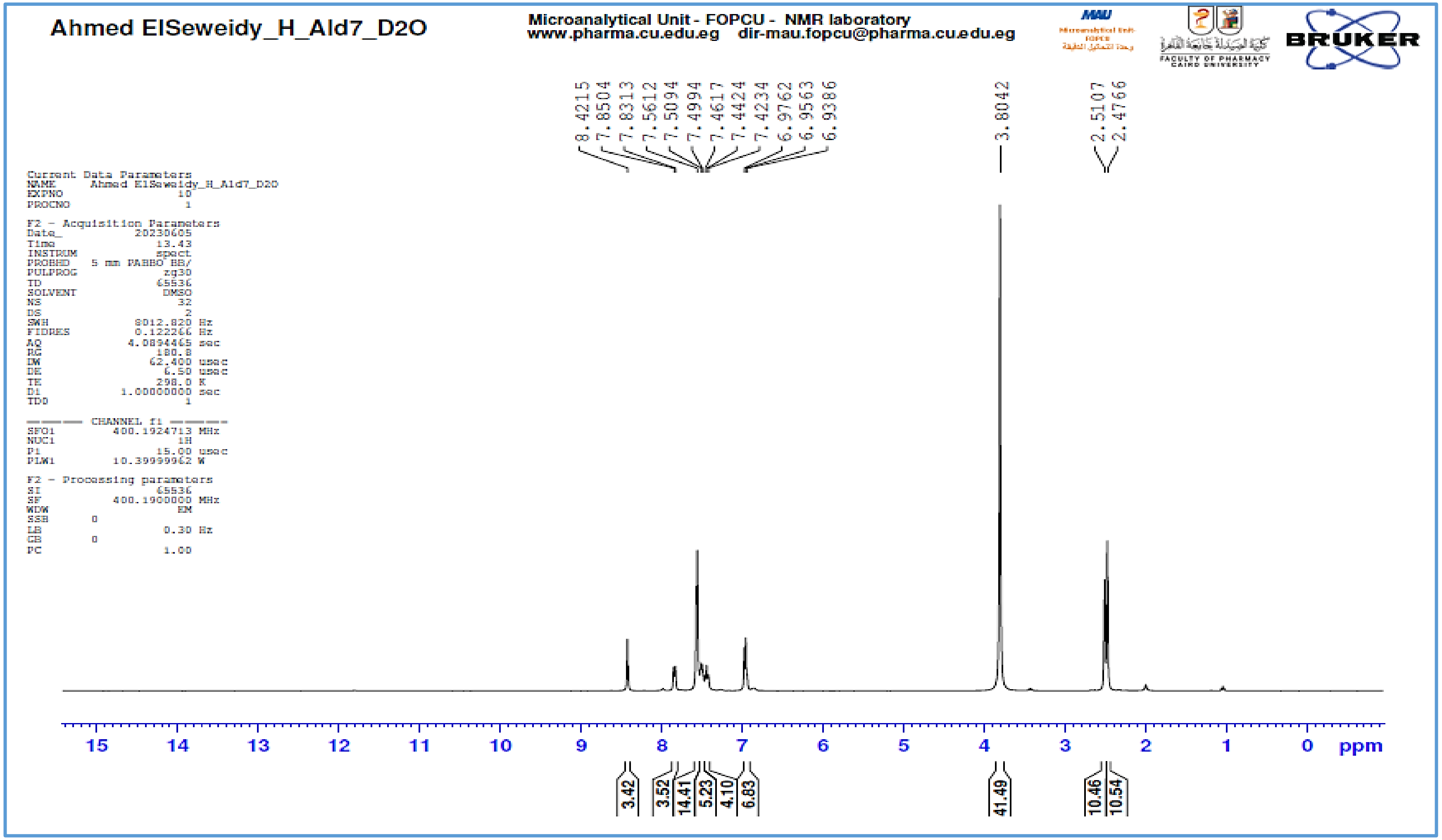

Fig. S19. ^{1}H NMR spectrum (DMSO-d_6+D_2O) of compound **3**

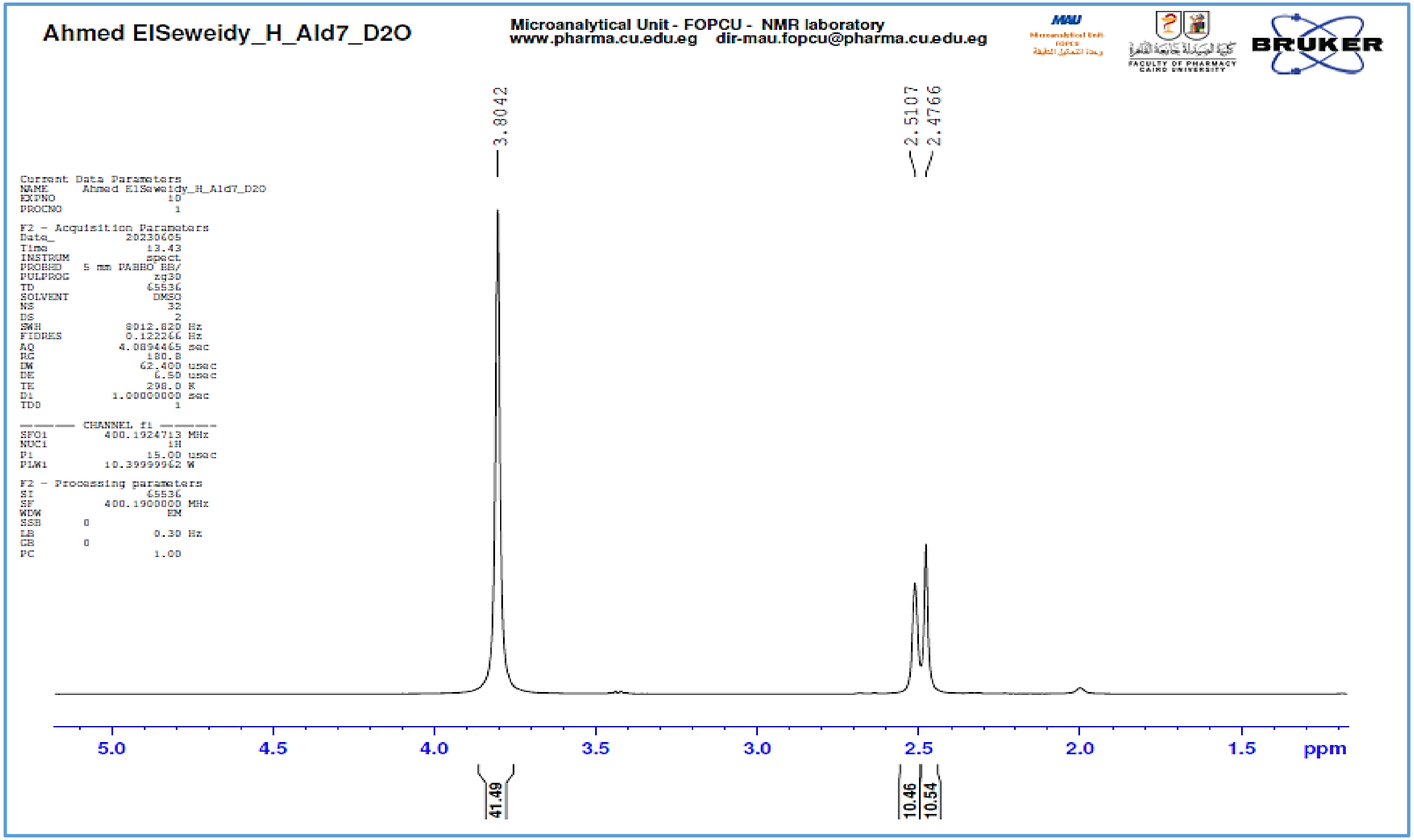

Fig. S20. Cont. ^{1}H NMR spectrum (DMSO-d_6+D_2O) of compound **3**

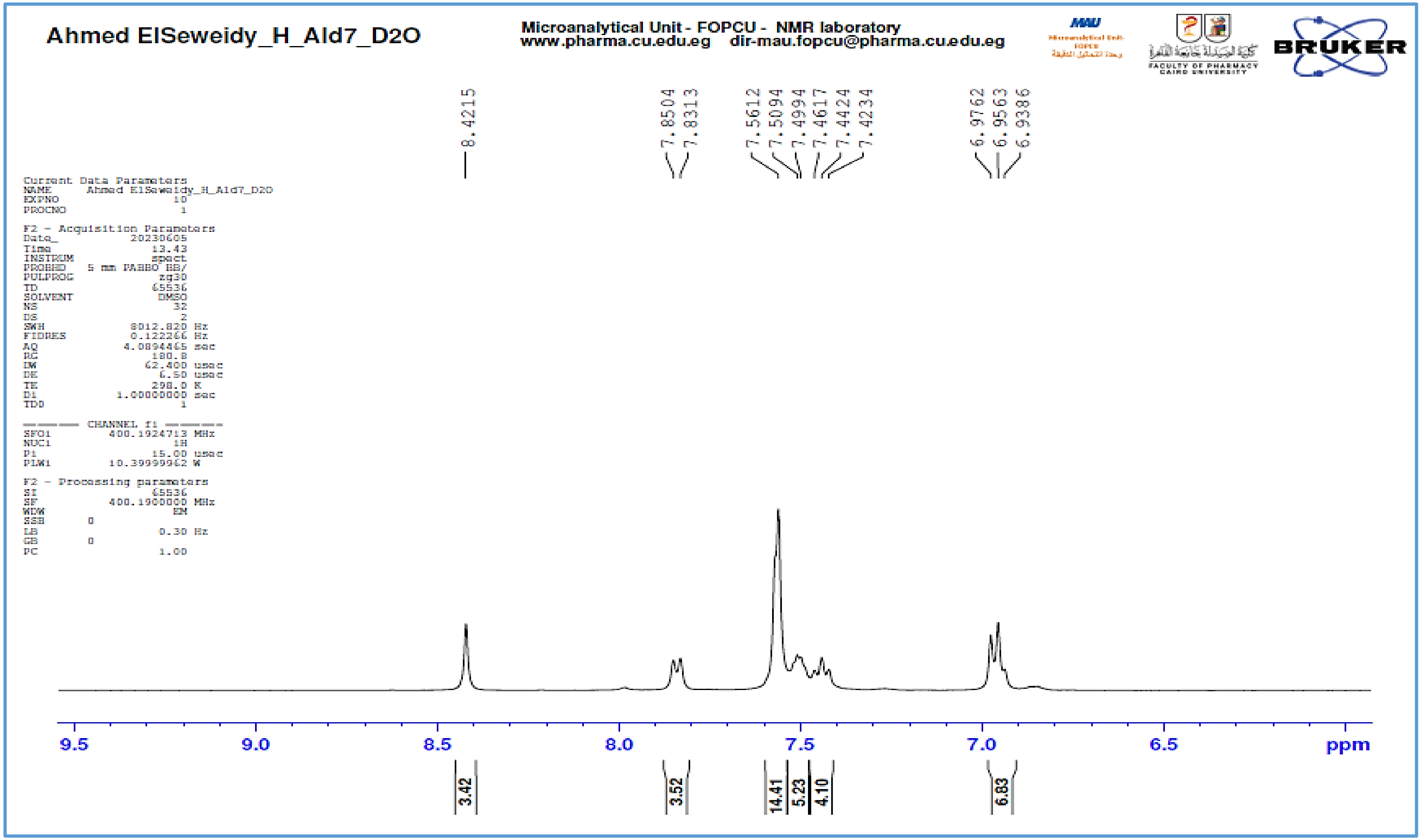

Fig. S21. Cont. ^{1}H NMR spectrum (DMSO-d_6+D_2O) of compound **3**

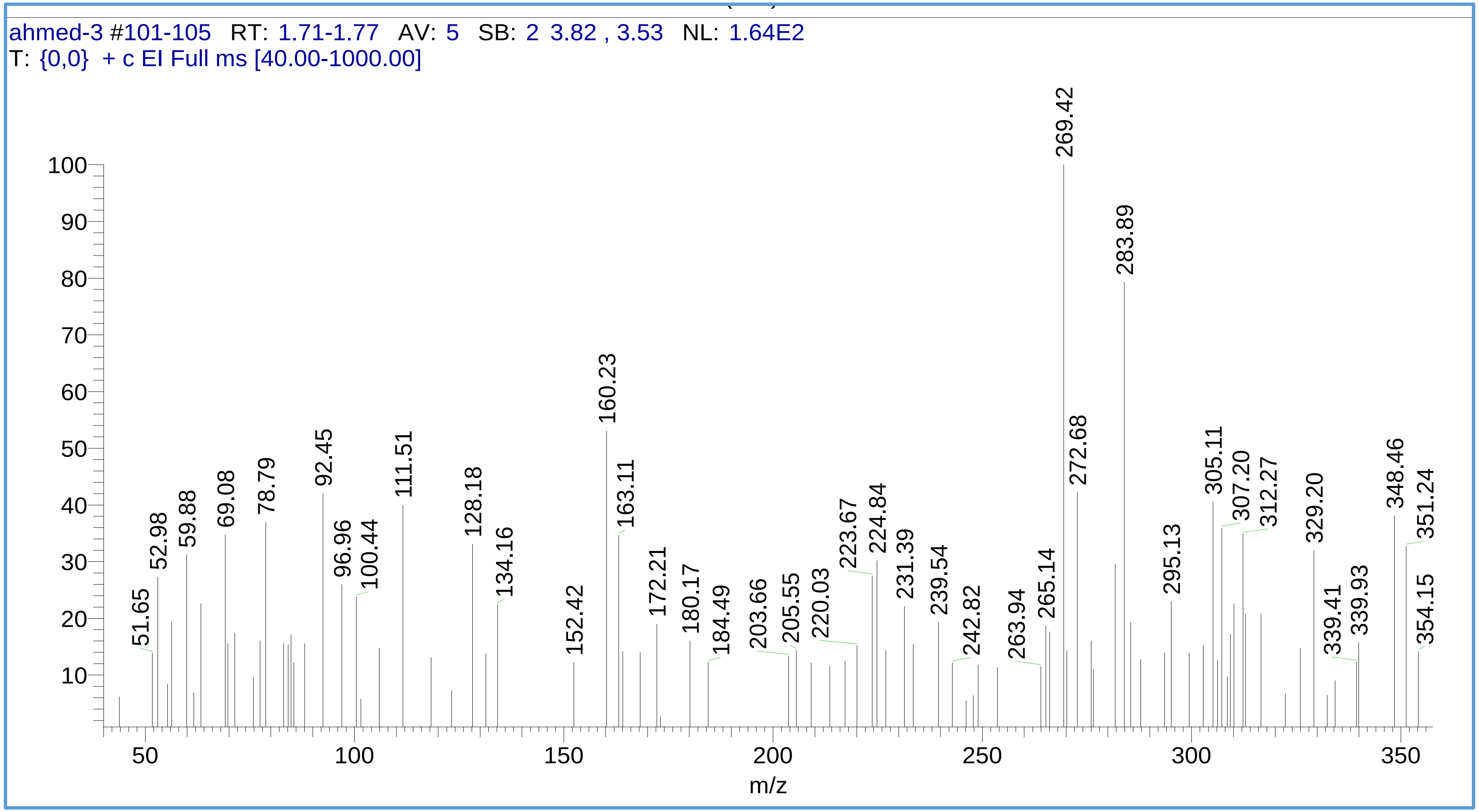

Fig. S22. EI-Mass spectrum of compound **3**

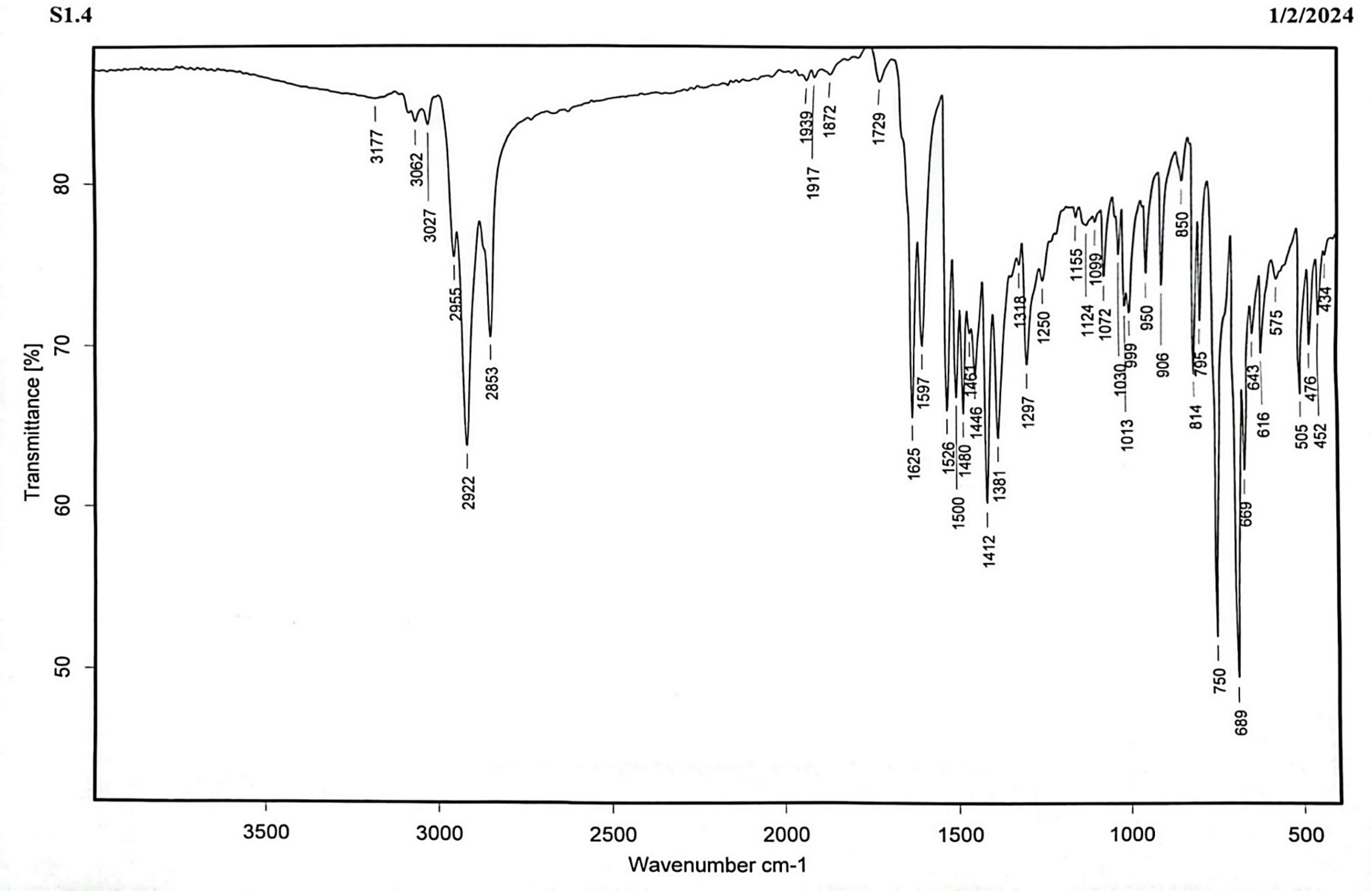

Fig. S23. IR spectrum of compound **4**

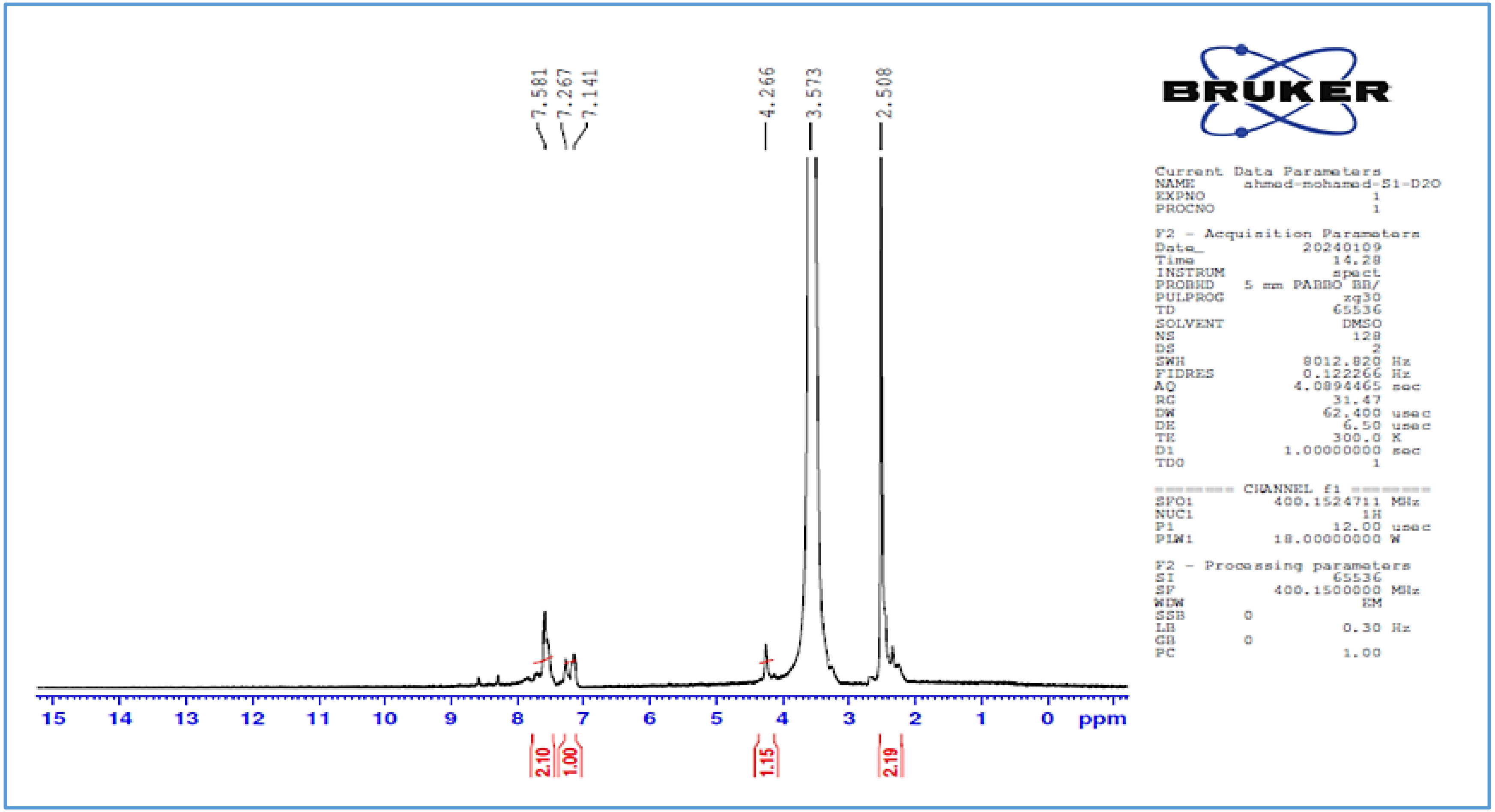

Fig. S24. ^{1}H NMR spectrum (DMSO-d_6) of compound **4**

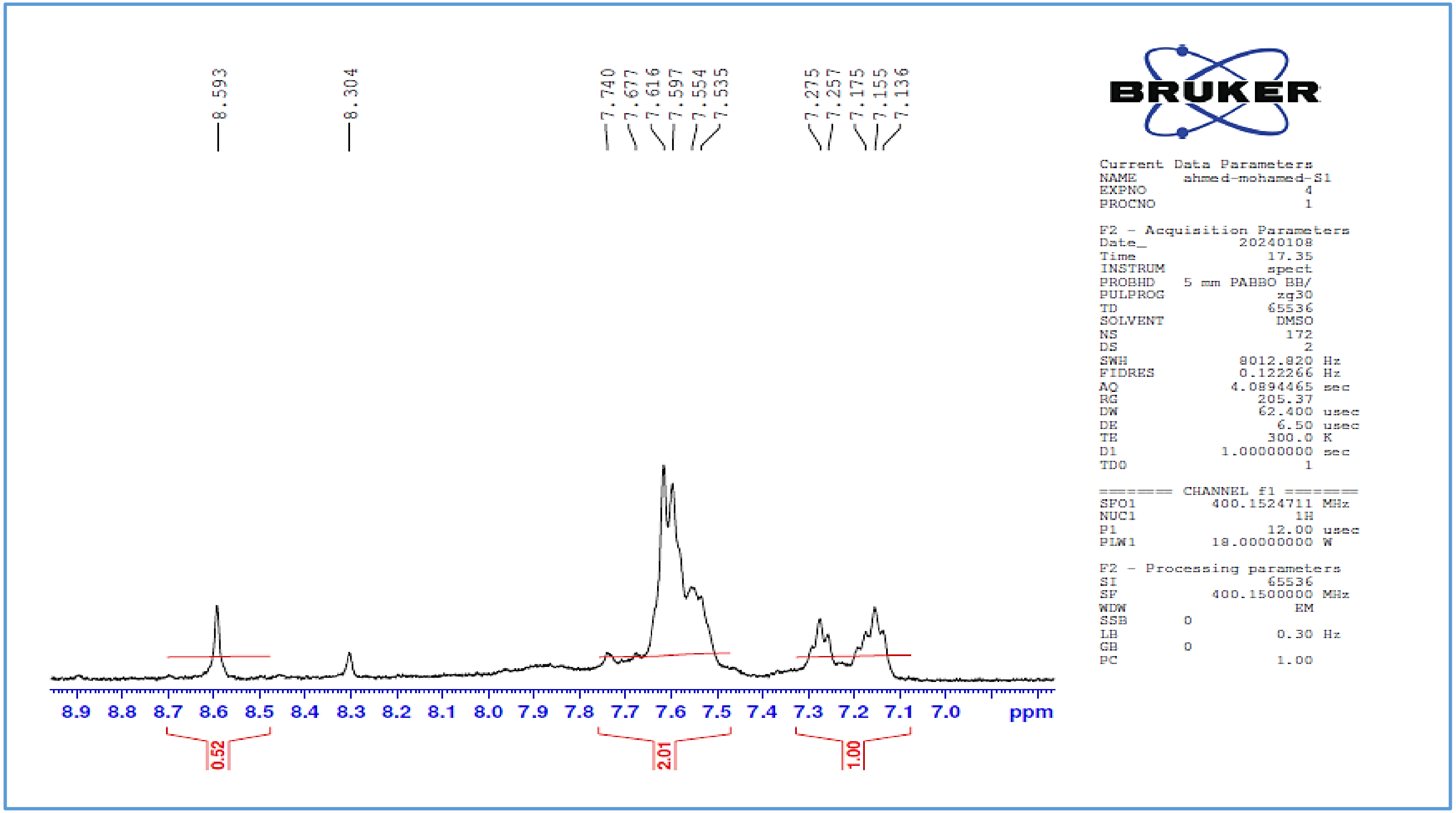

Fig. S25. Cont. ^{1}H NMR spectrum (DMSO-d_6) of compound **4**

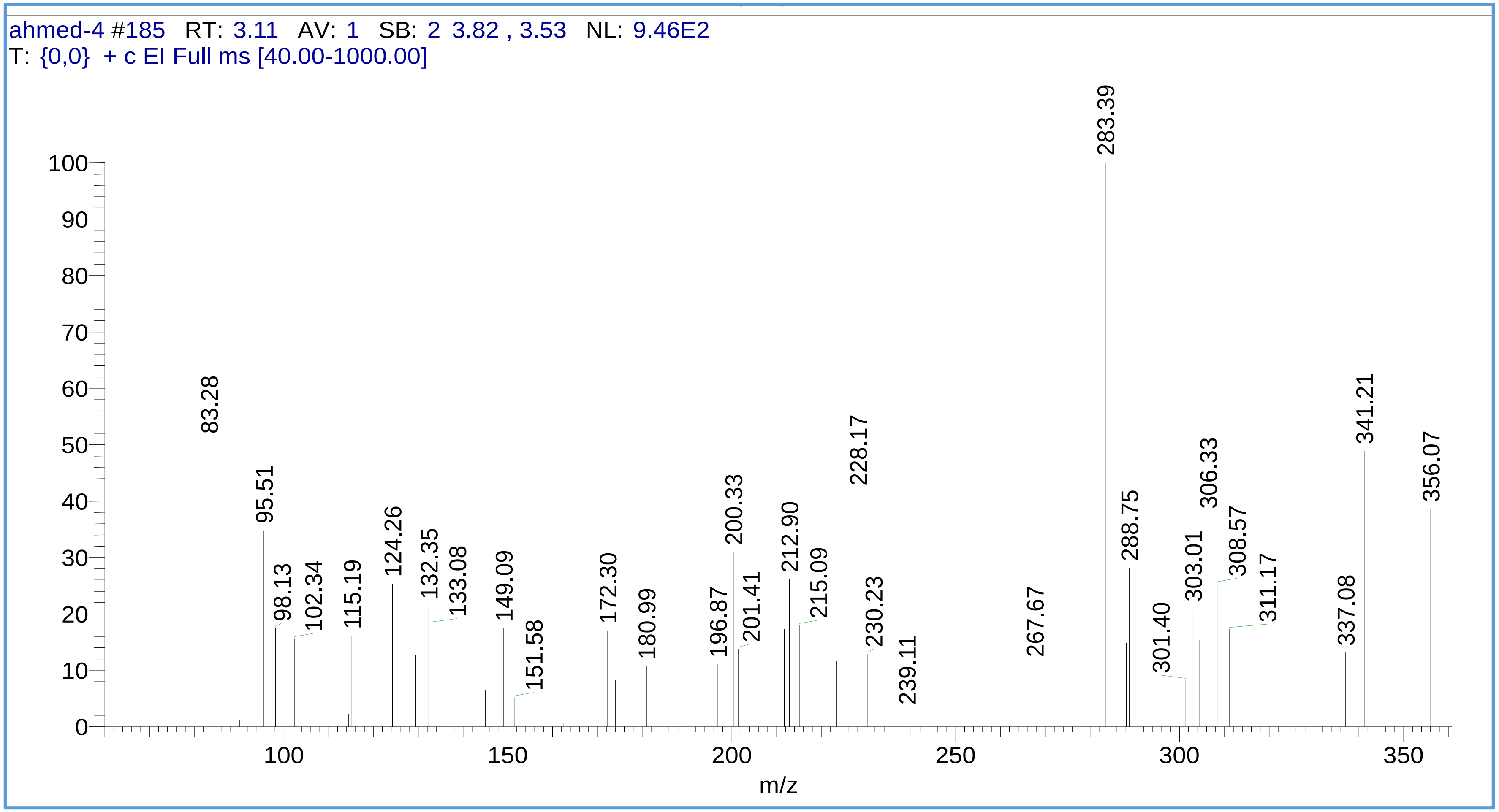

Fig. S26. EI-Mass spectrum of compound **4**

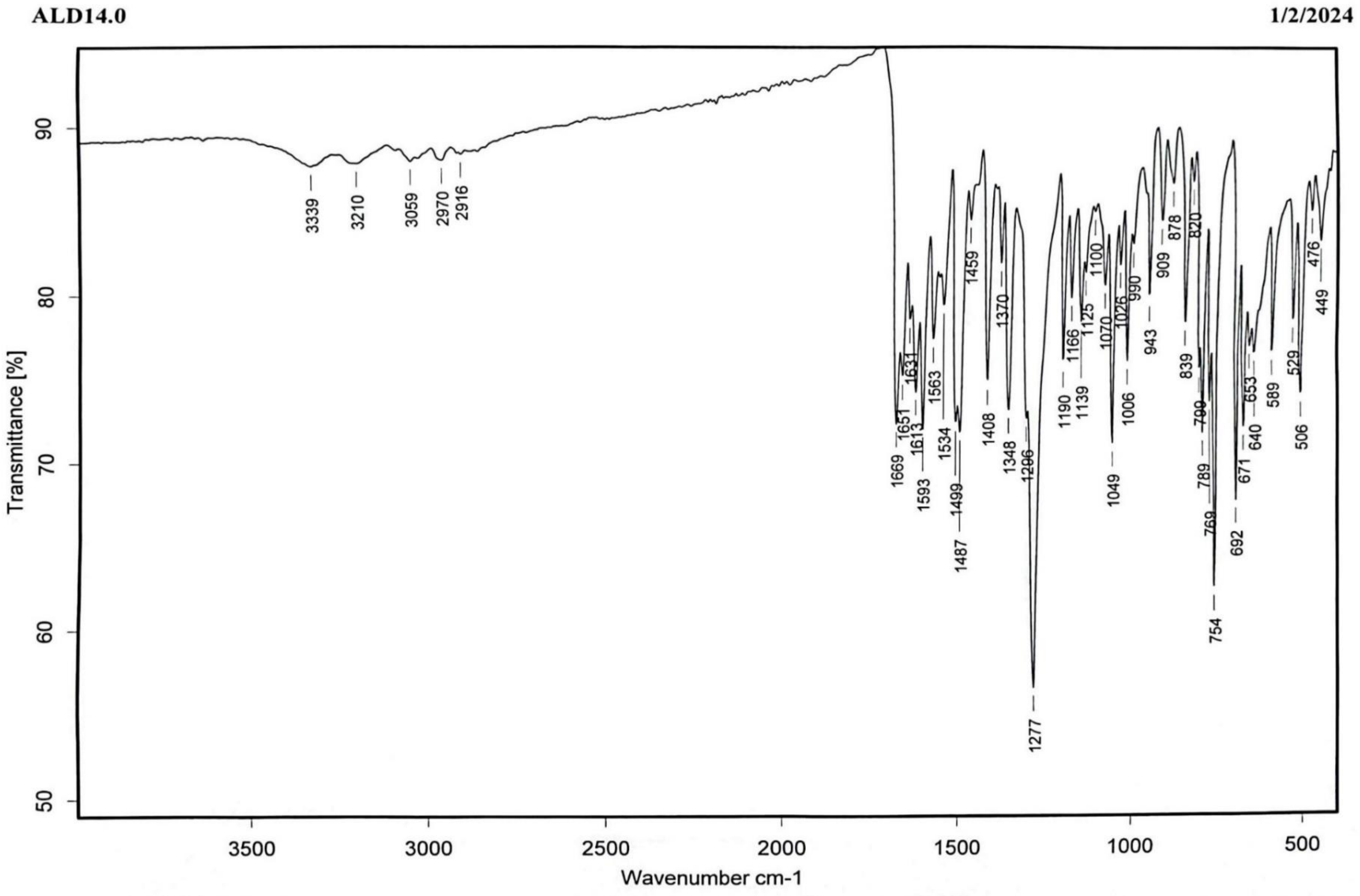

Fig. S27. IR spectrum of compound **6**

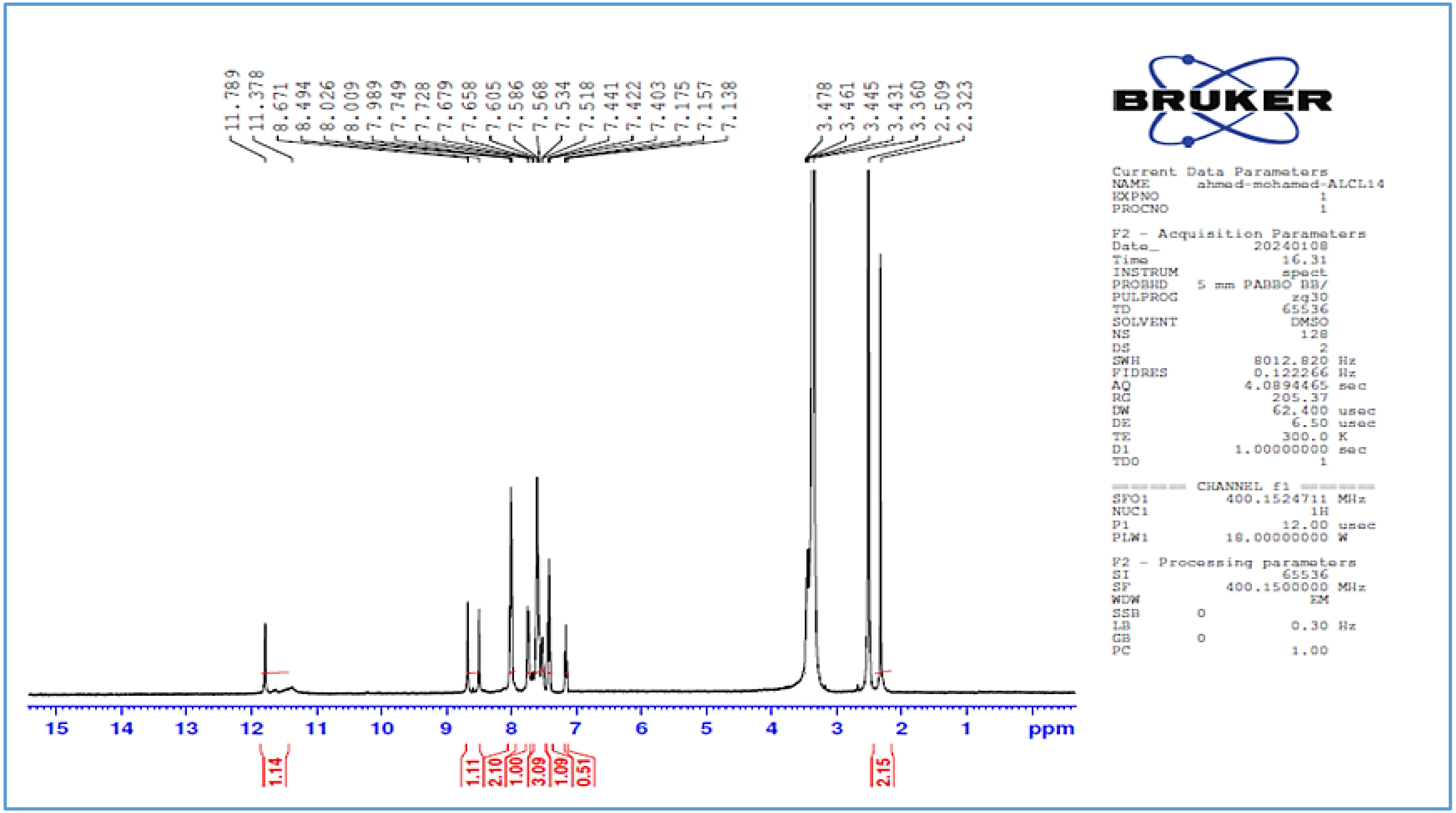

Fig. S28. ^{1}H NMR spectrum (DMSO-d_6) of compound **6**

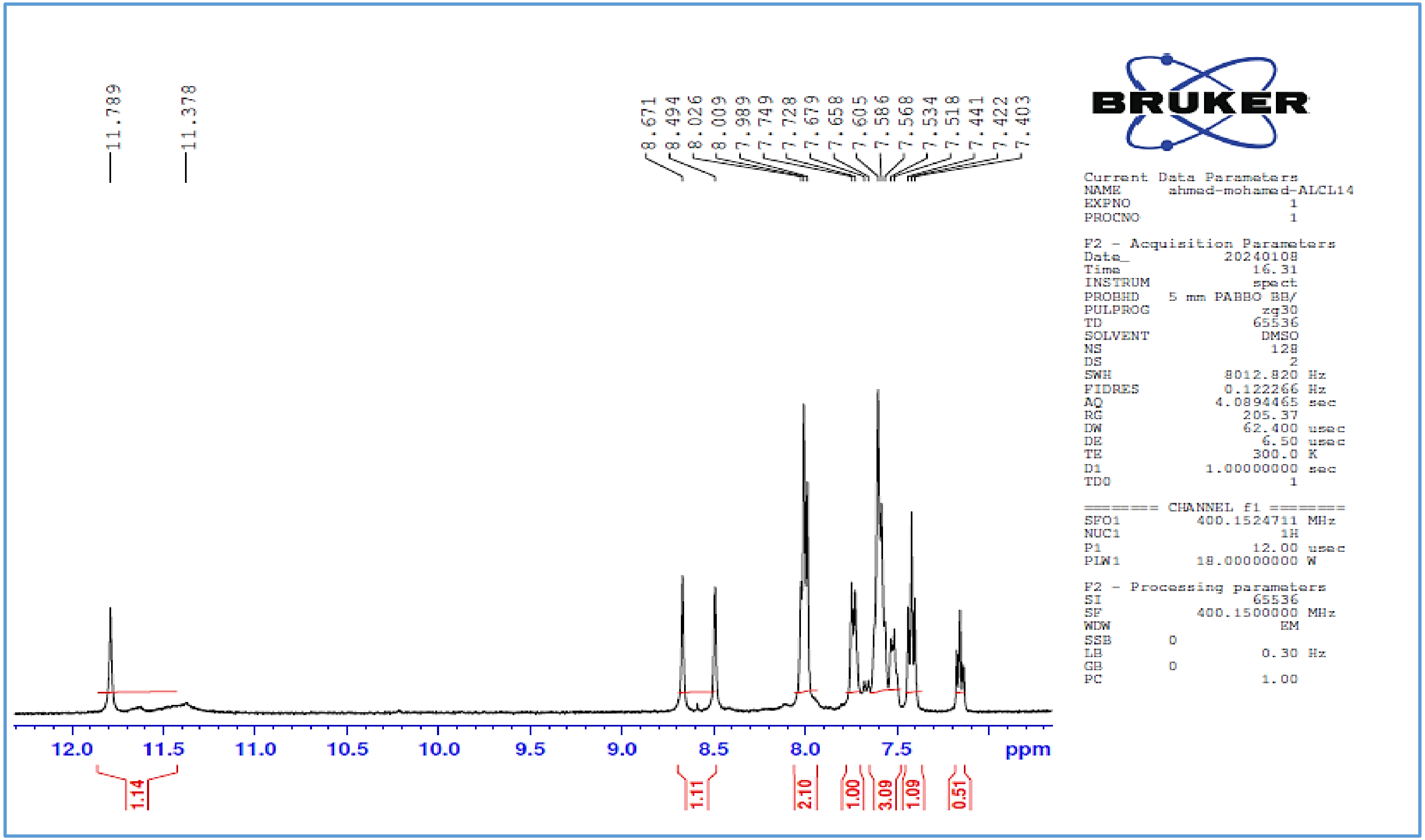

Fig. S29. Cont. ^{1}H NMR spectrum (DMSO-d_6) of compound **6**

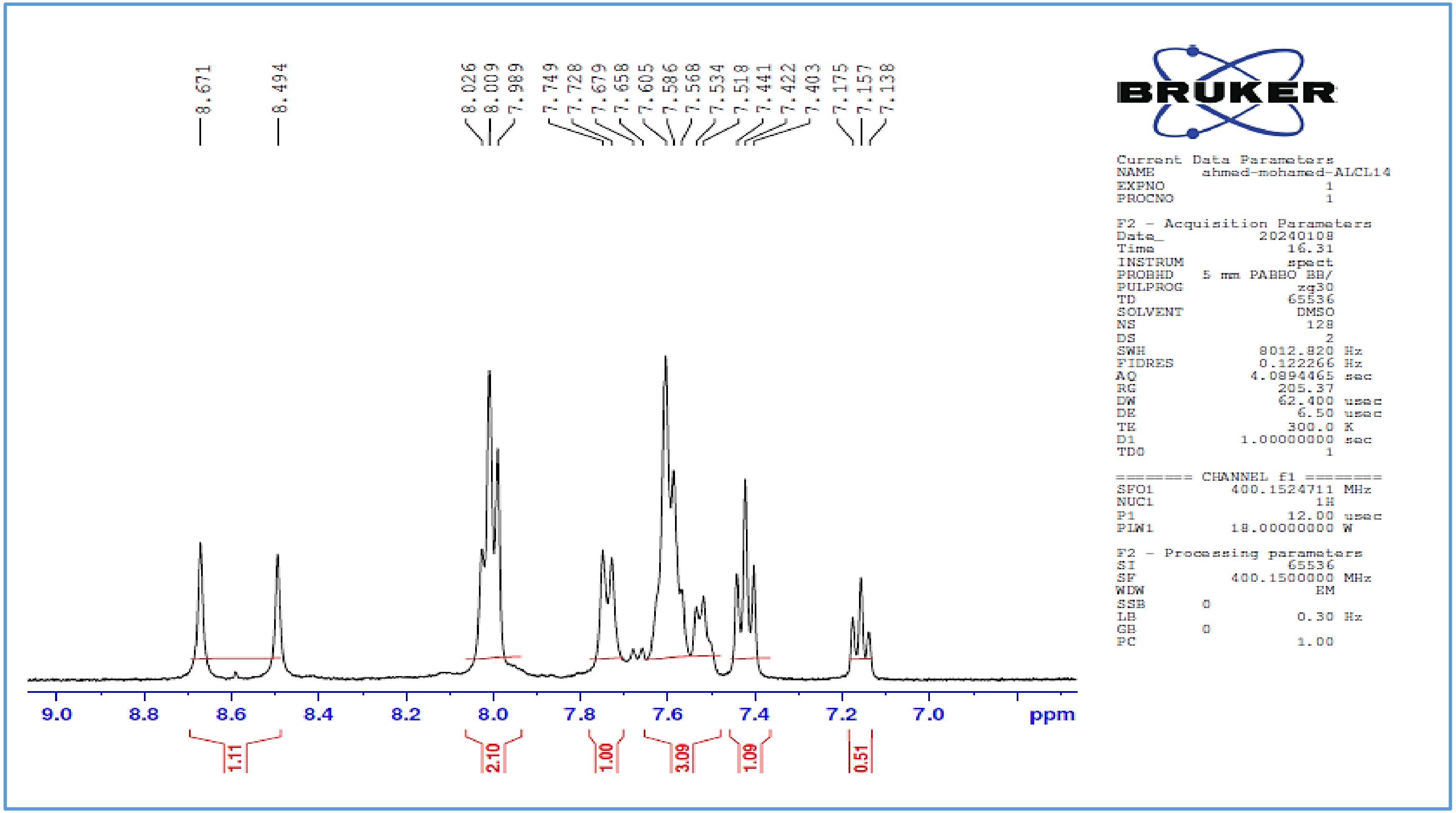

Fig. S30. Cont. ^{1}H NMR spectrum (DMSO-d_6) of compound **6**

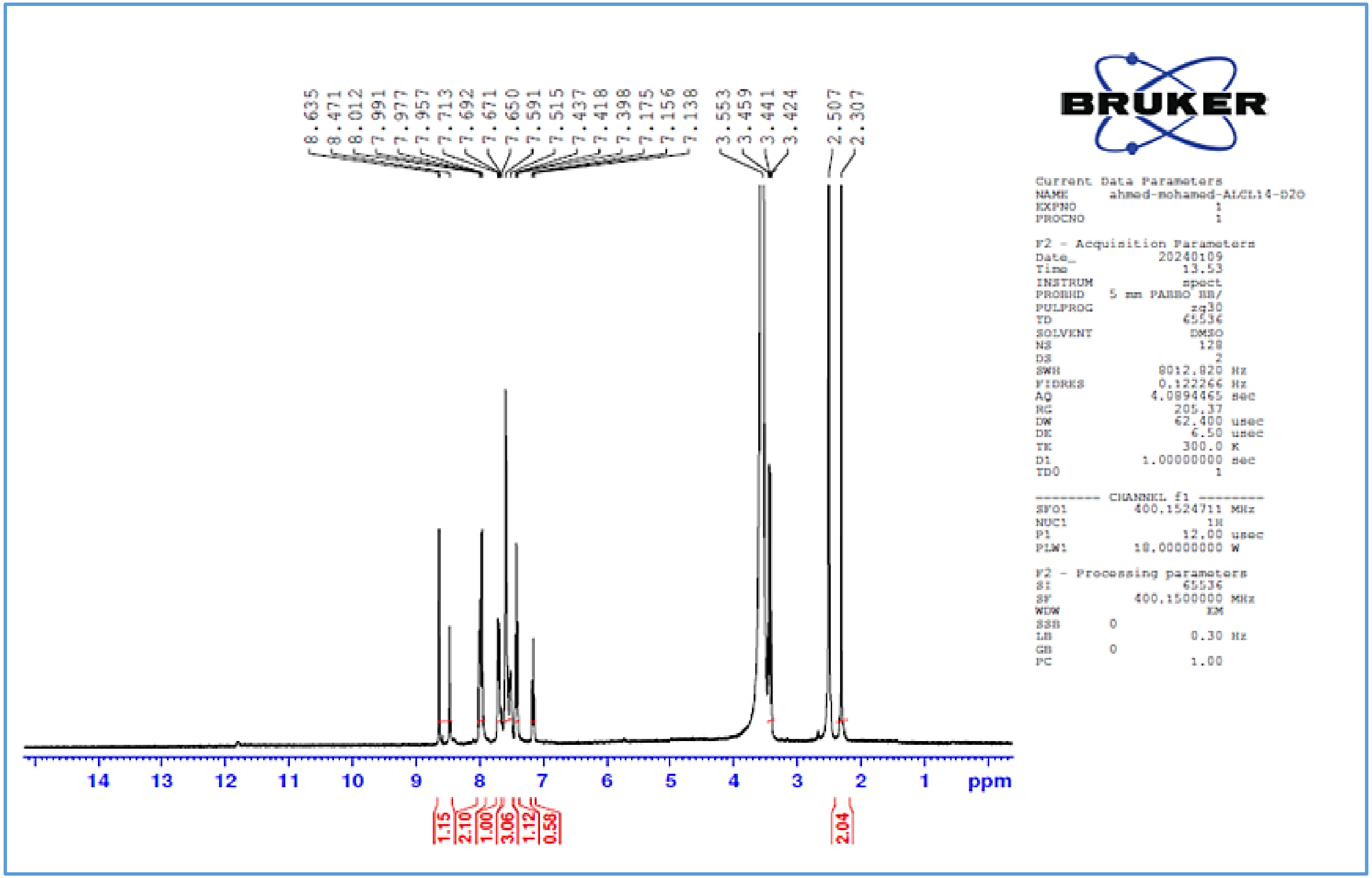

Fig. S31. ^{1}H NMR spectrum (DMSO-d_6+D_2O) of compound **6**

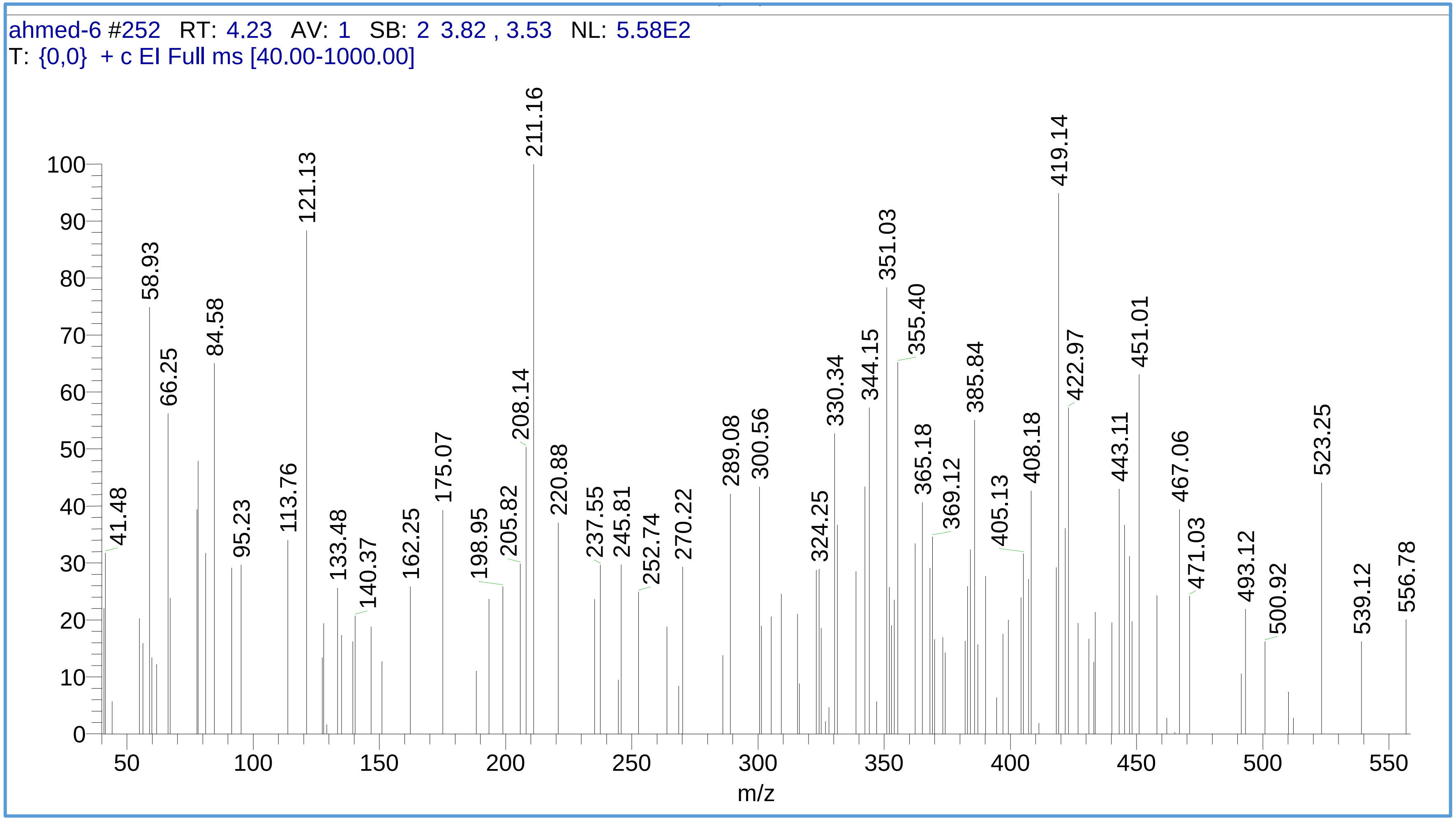

Fig. S32. EI-Mass spectrum (DMSO-d_6) of compound **6**

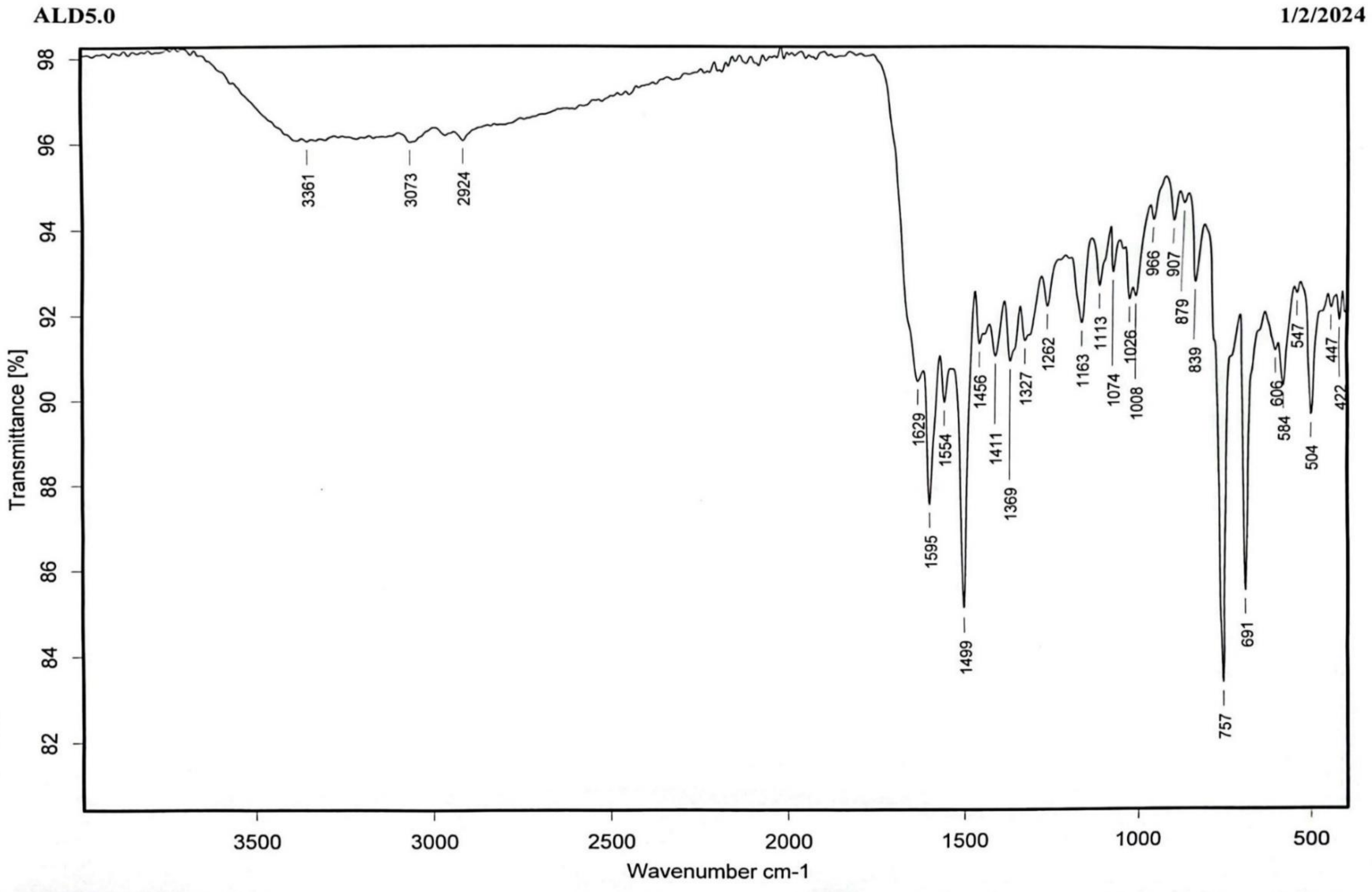

Fig. S33. IR spectrum of compound **7**

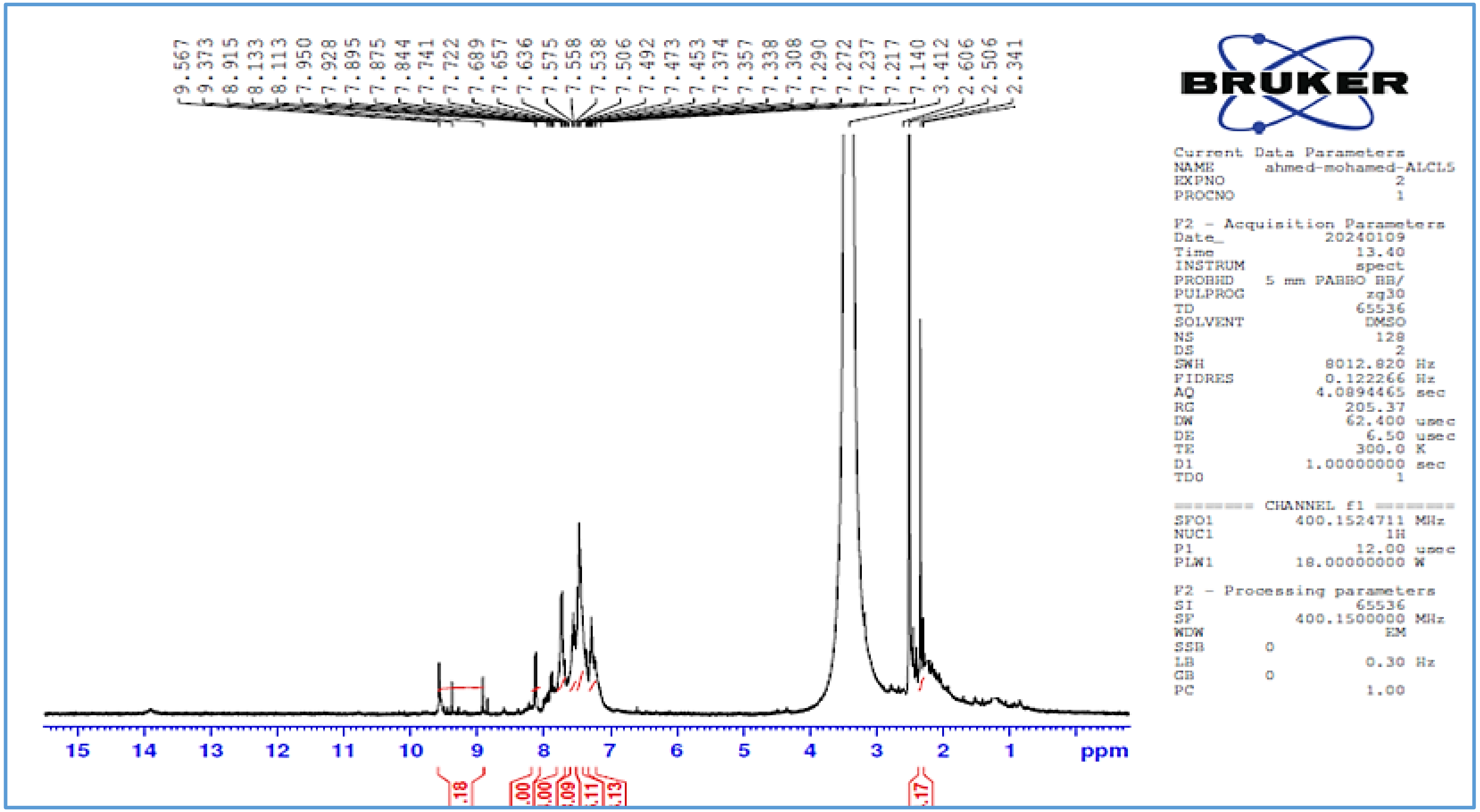

Fig. S34. ^{1}H NMR spectrum (DMSO-d_6) of compound **7**

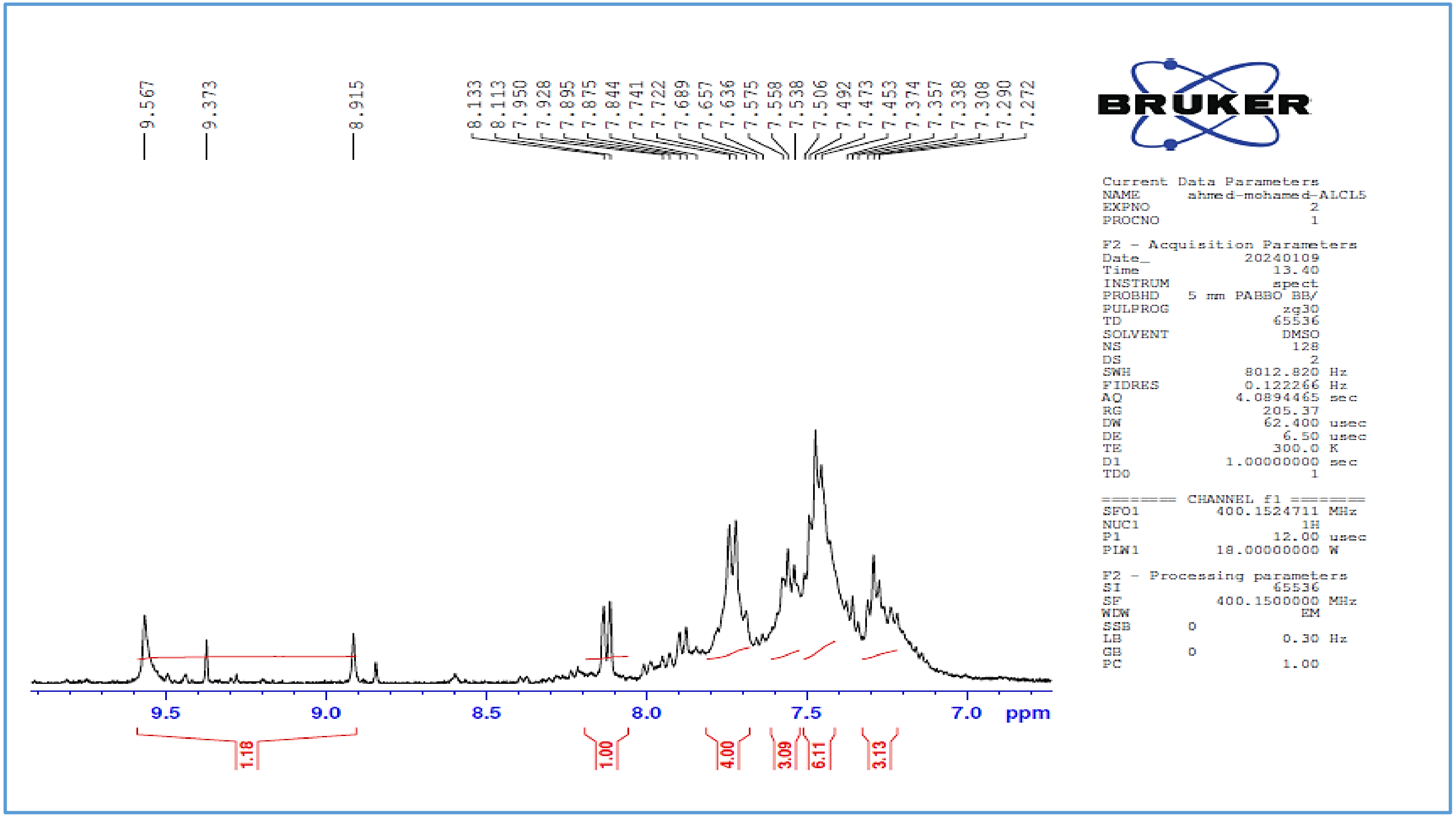

Fig. S35. Cont. ^{1}H NMR spectrum (DMSO-d_6) of compound **7**

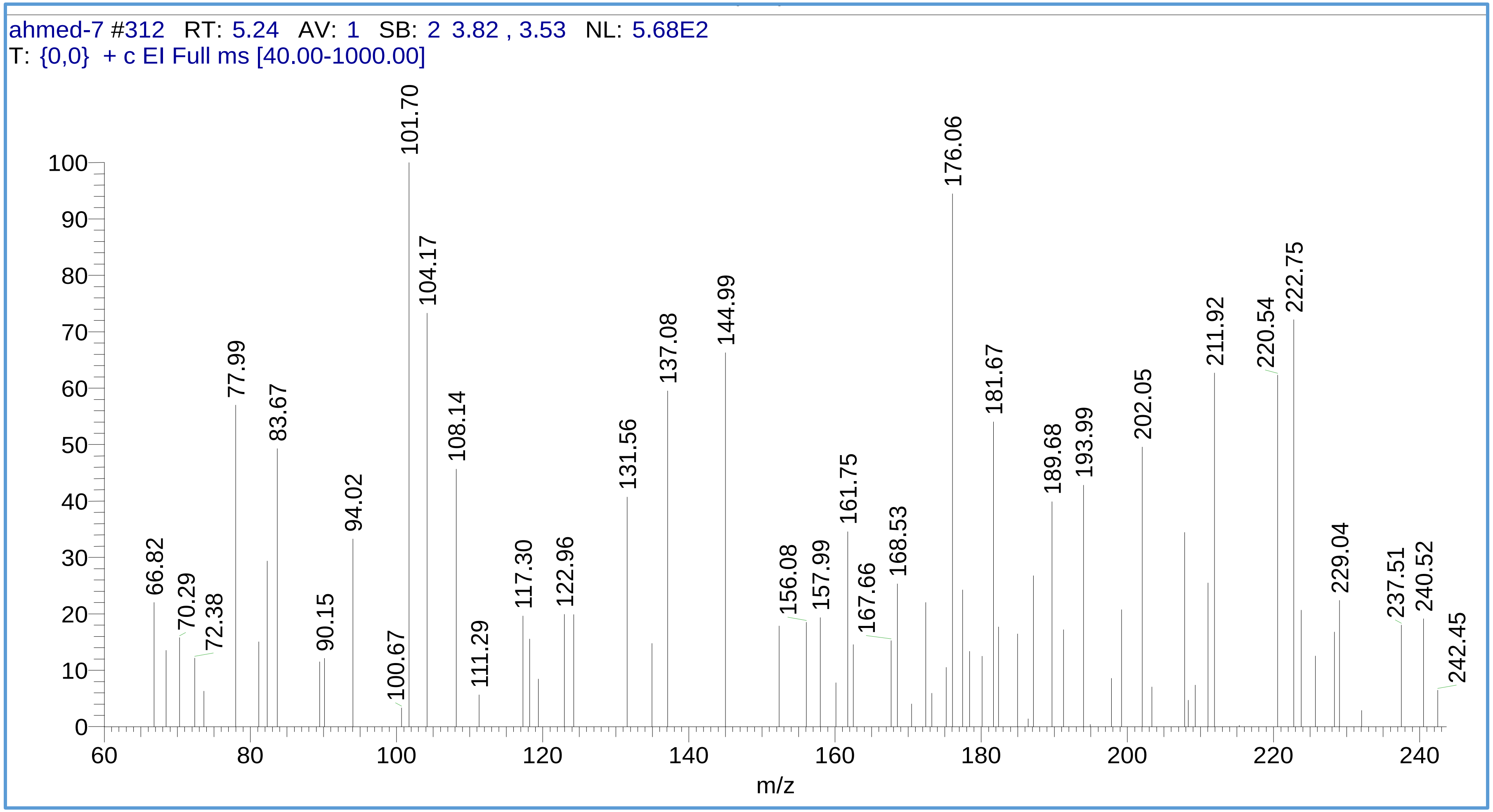

Fig. S36. EI-Mass spectrum of compound **7**

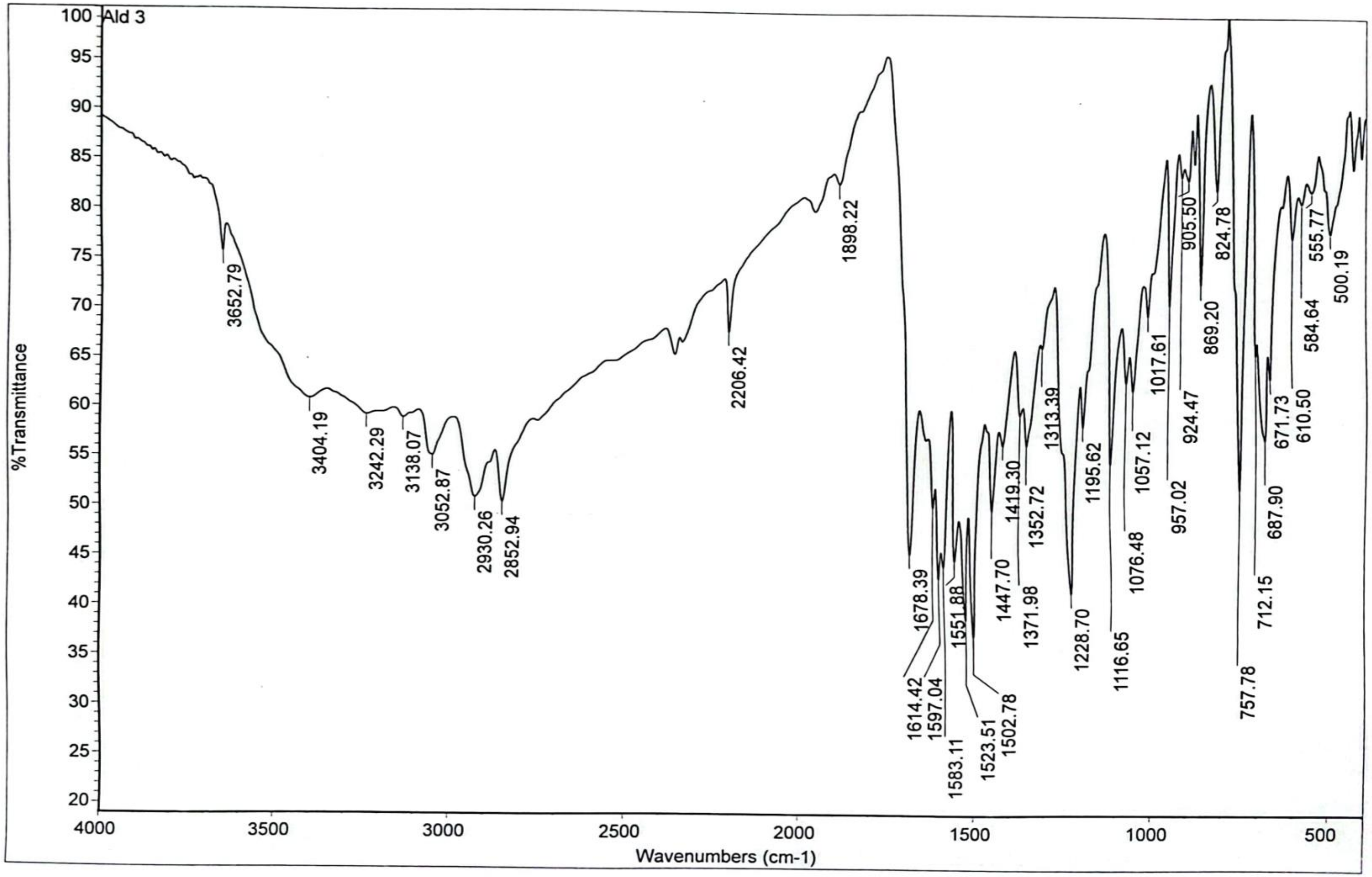

Fig. S37. IR spectrum of compound **8**

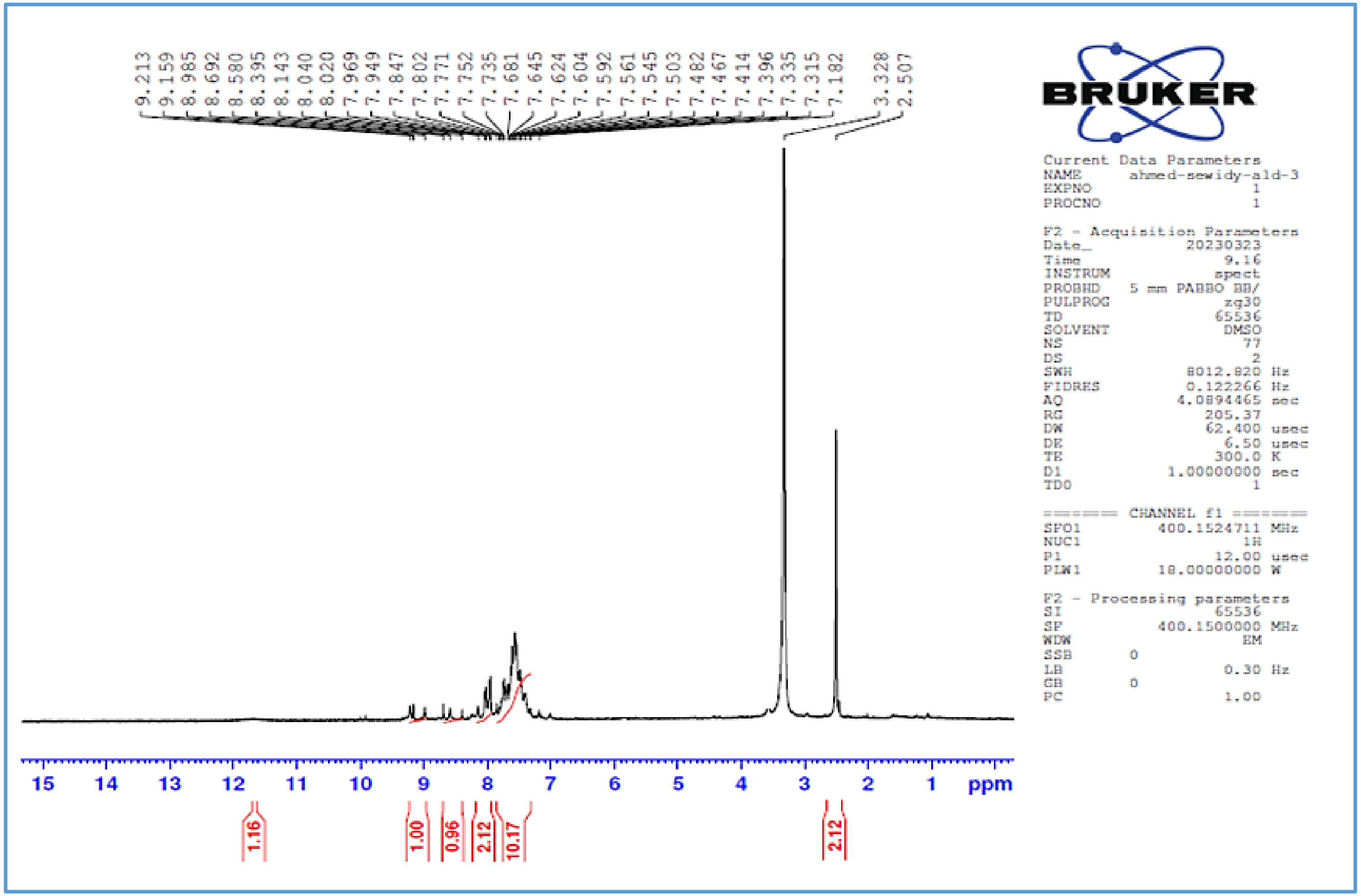

Fig. S38. ^{1}H NMR spectrum (DMSO-*d*$_6$) of compound **8**

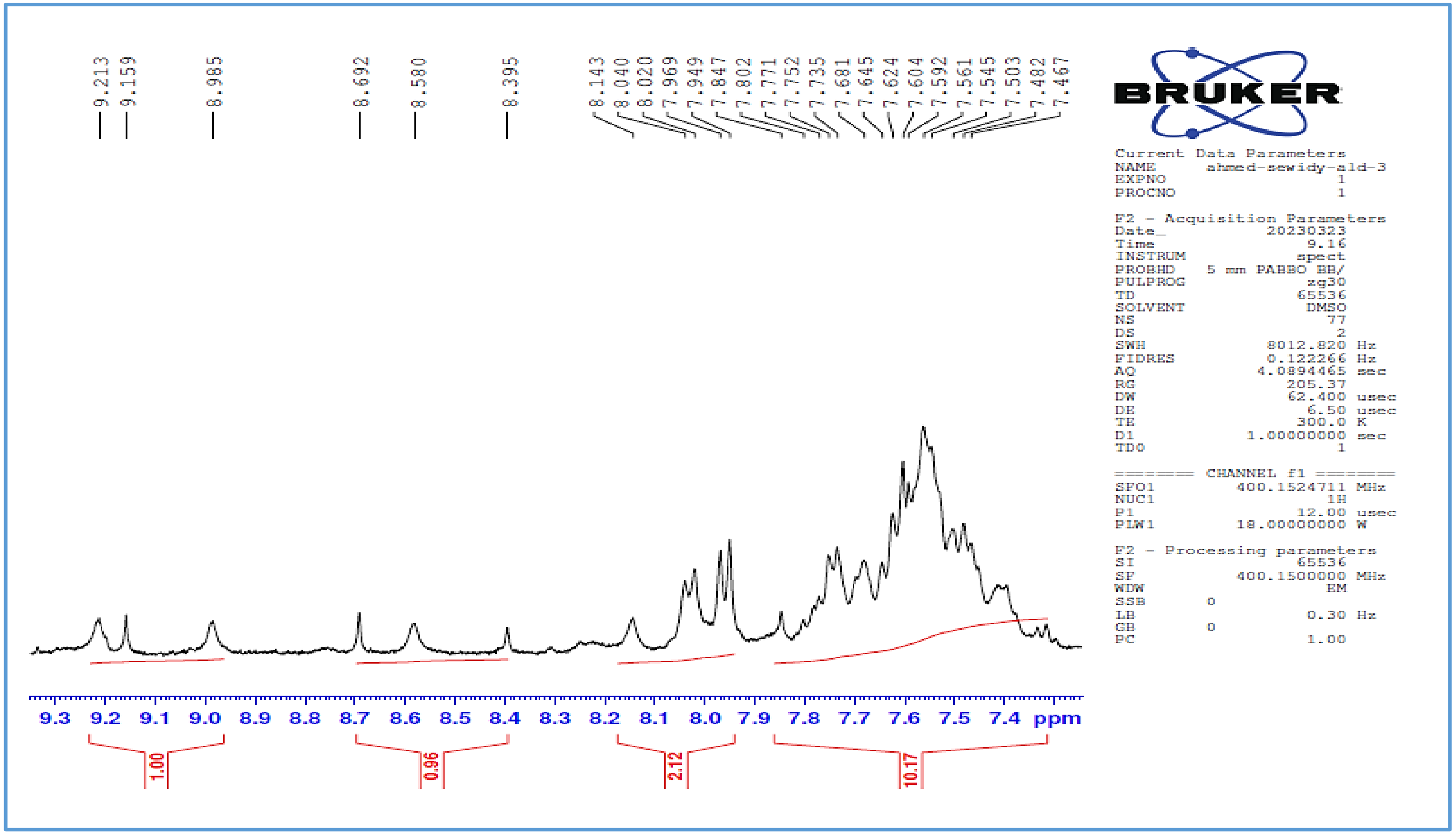

Fig. S39. Cont. ^{1}H NMR spectrum (DMSO-d_6) of compound **8**

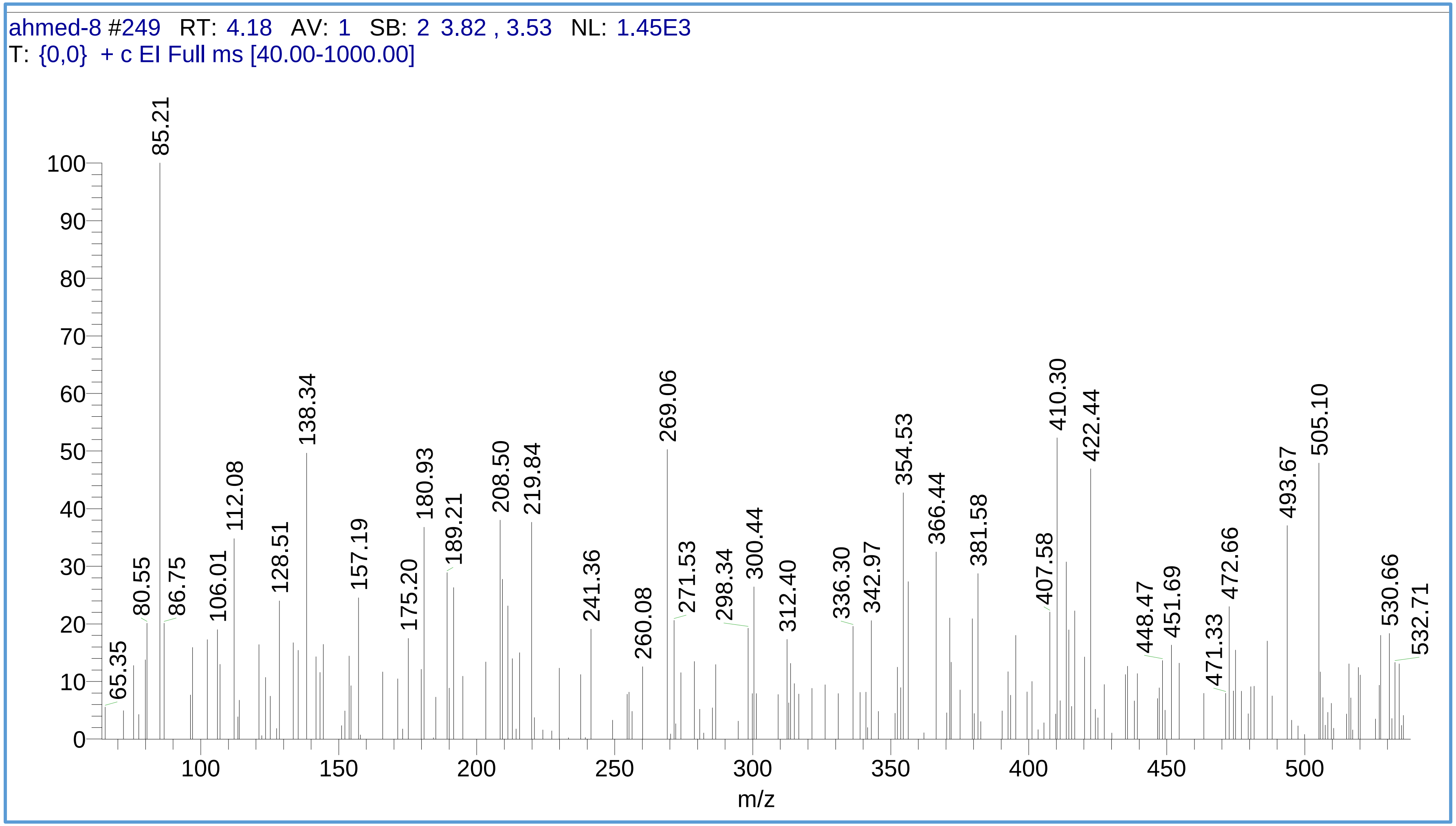

Fig. S40. EI-Mass spectrum of compound **8**

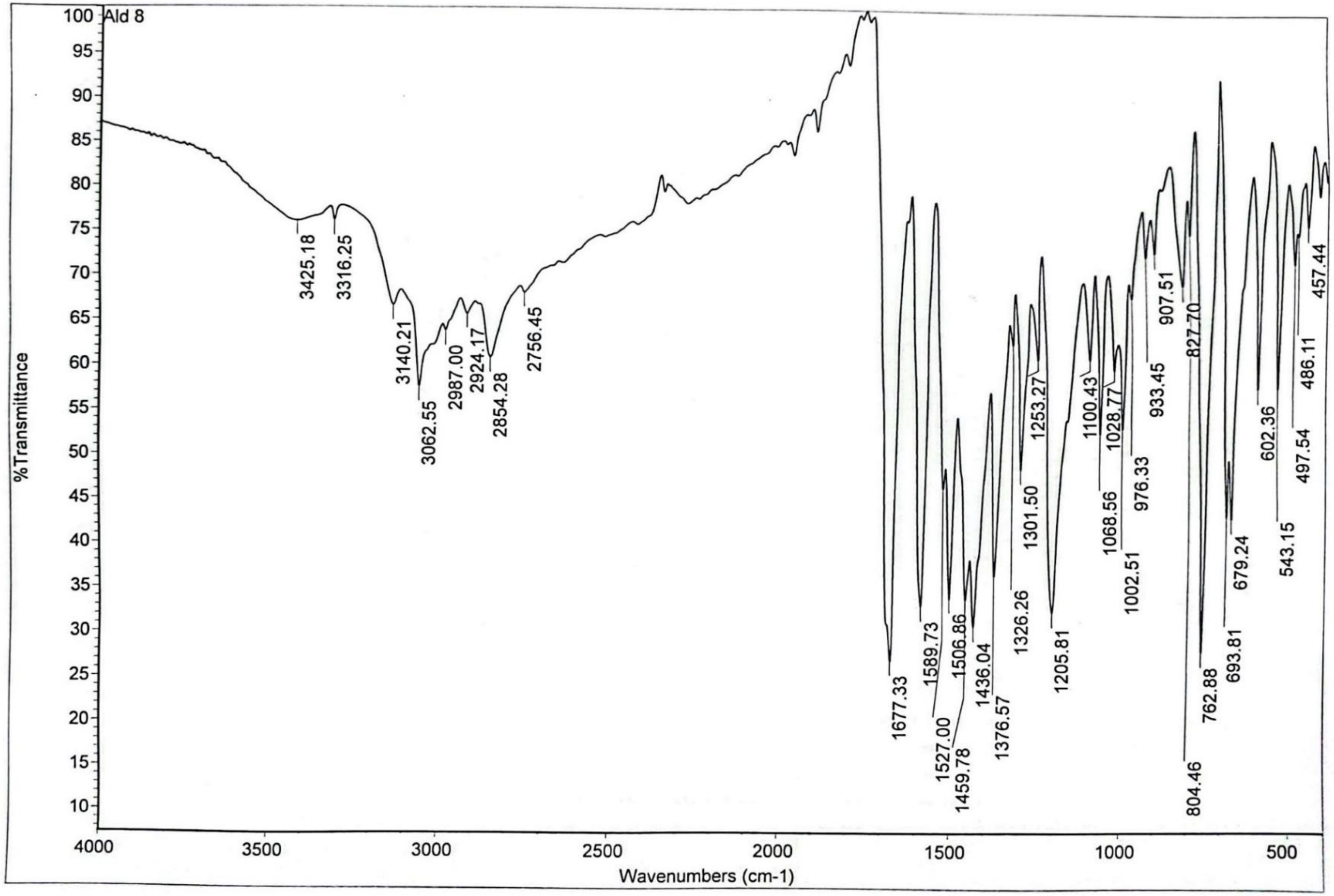

Fig. S41. IR spectrum of compound **9**

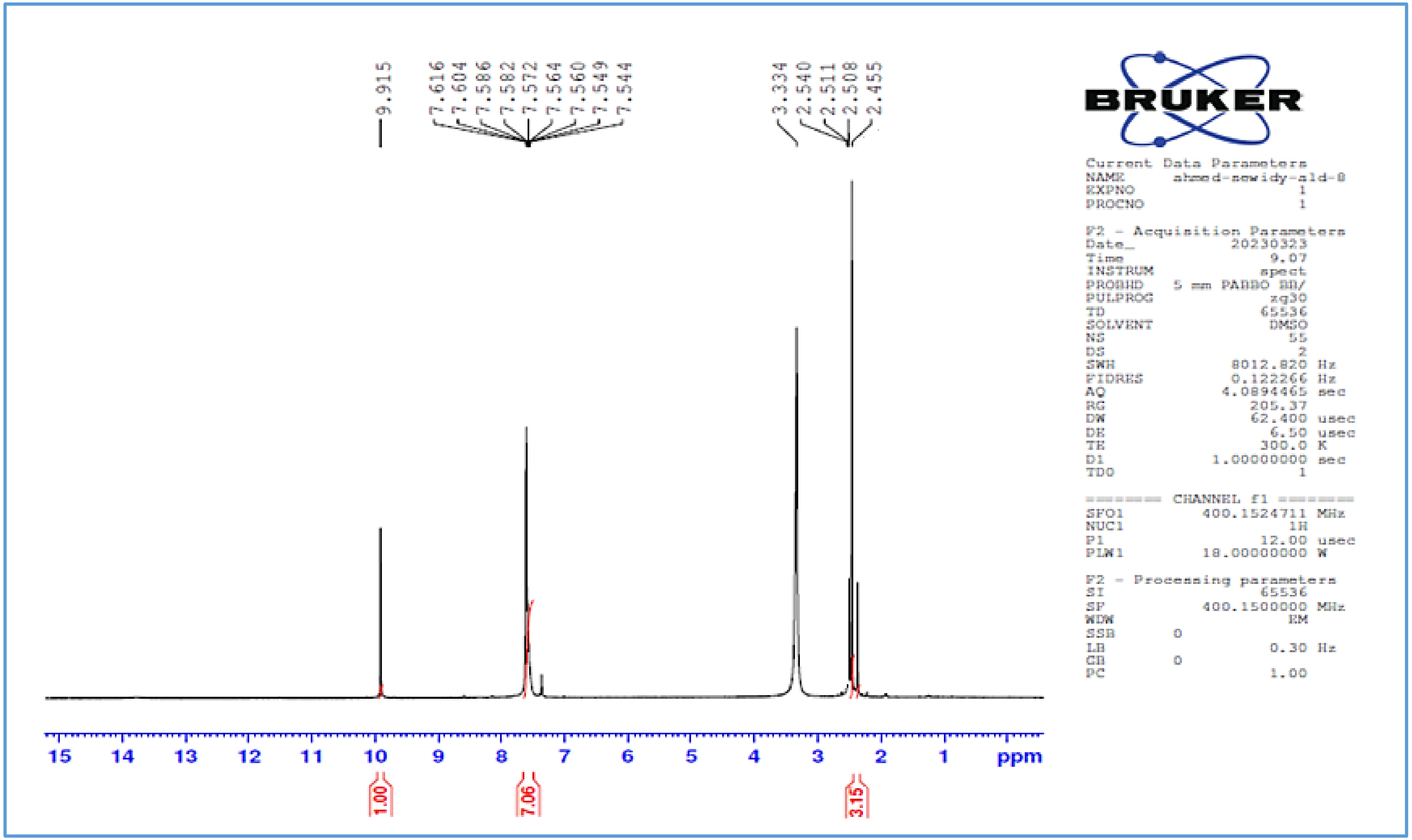

Fig. S42. ^{1}H NMR spectrum (DMSO-d_6) of compound **9**

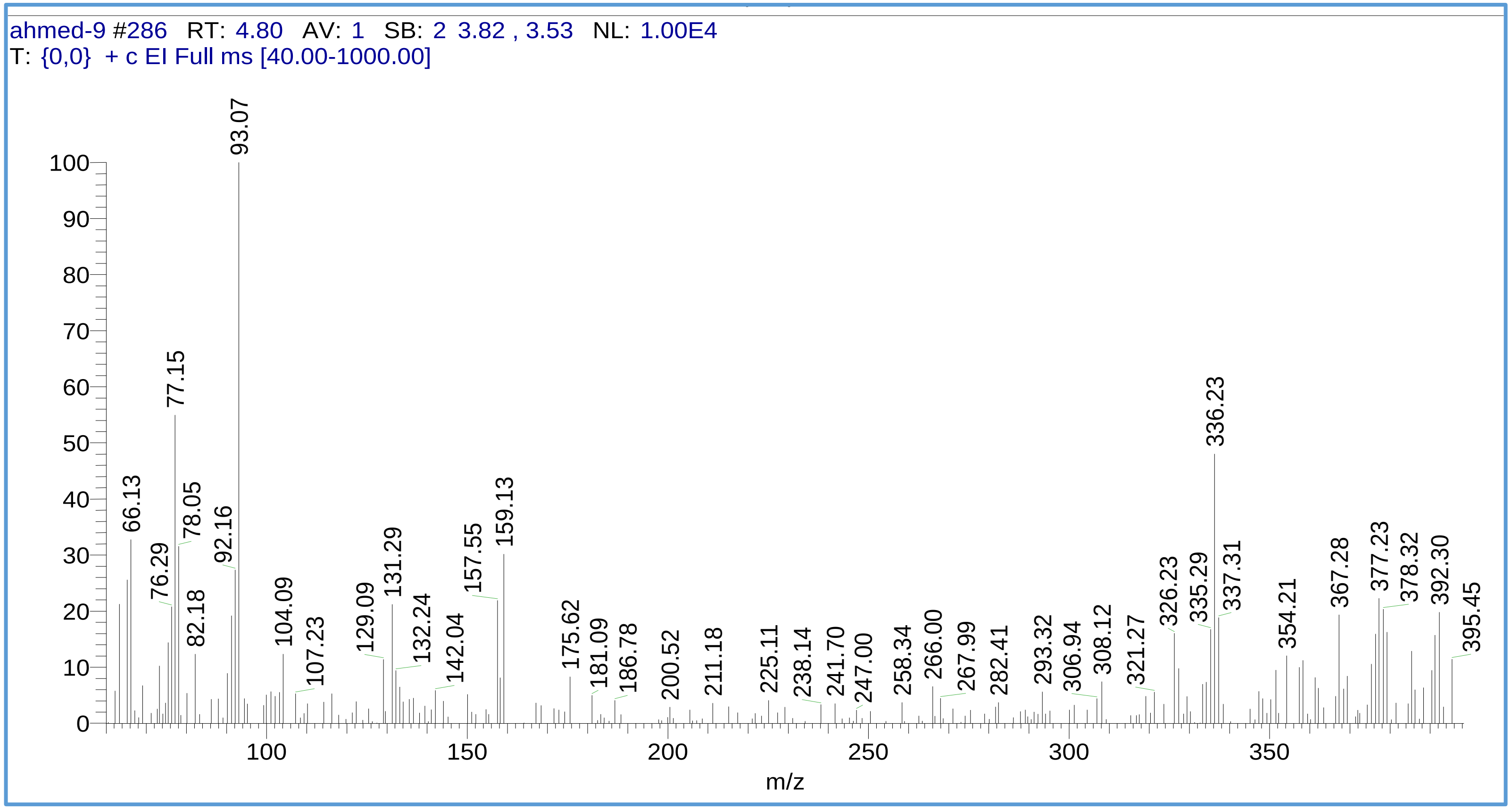

Fig. S43. EI-Mass spectrum of compound **9**

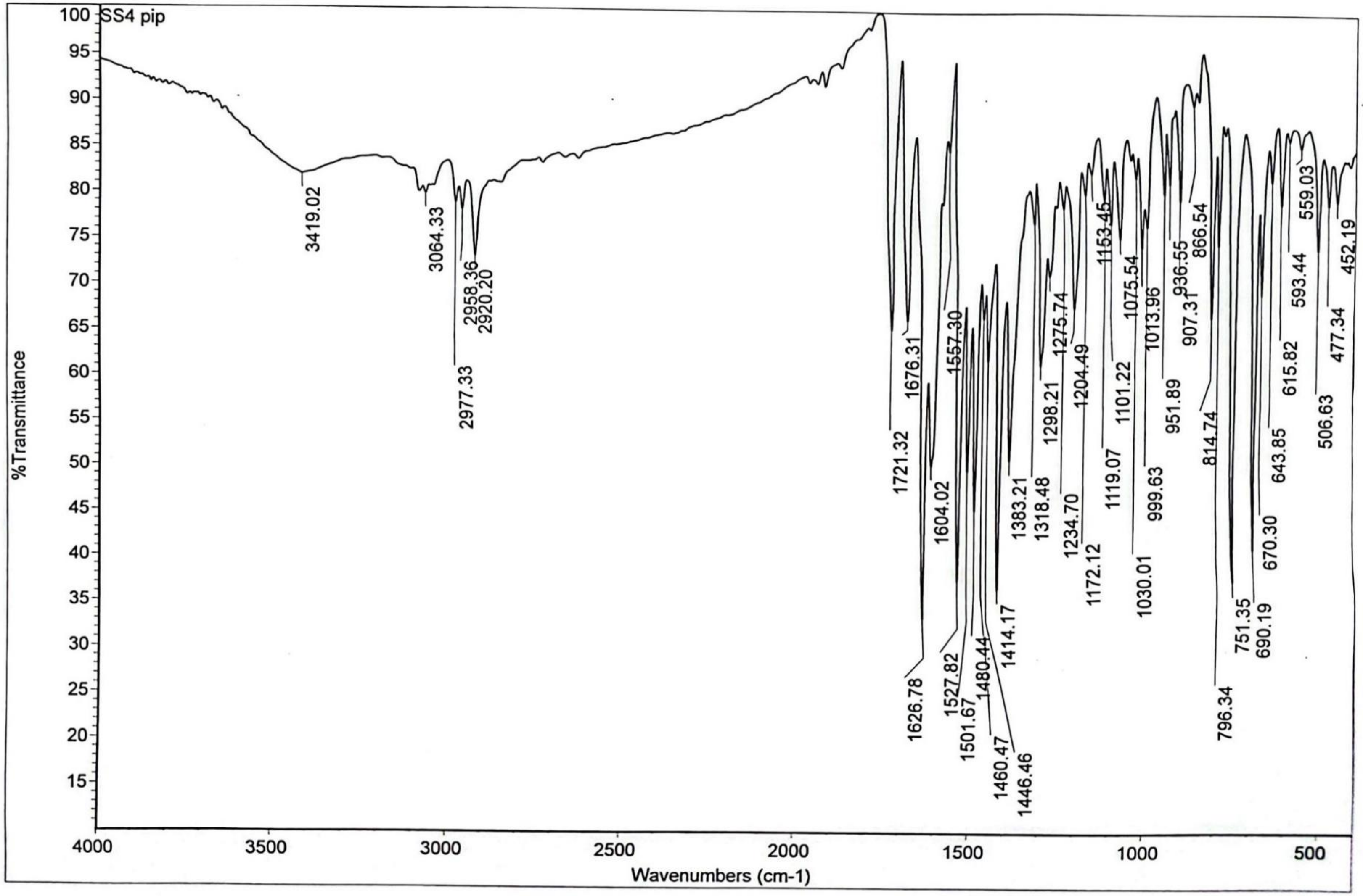

Fig. S44. IR spectrum of compound **10**

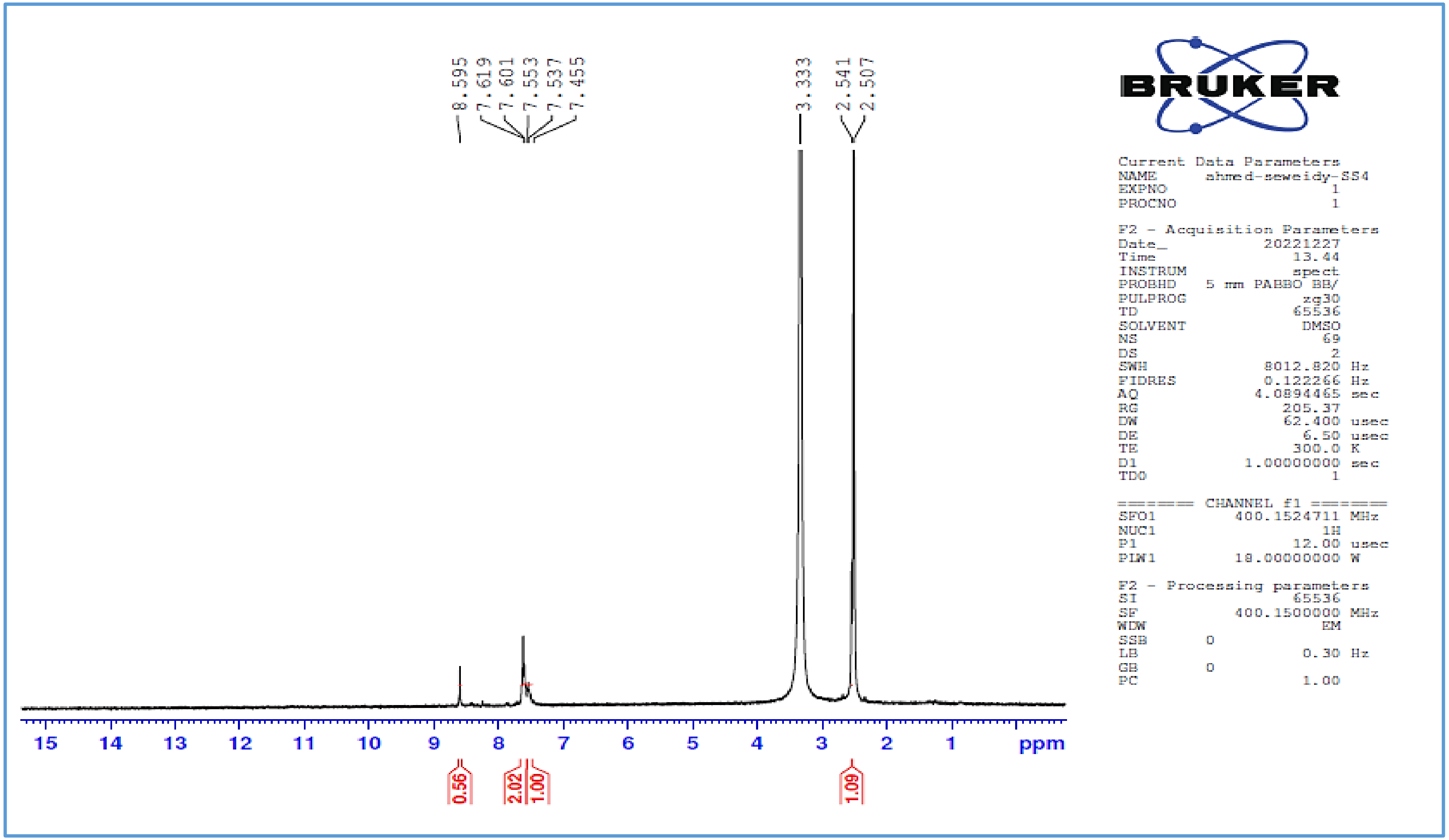

Fig. S45. ^{1}H NMR spectrum (DMSO-d_6) of compound **10**

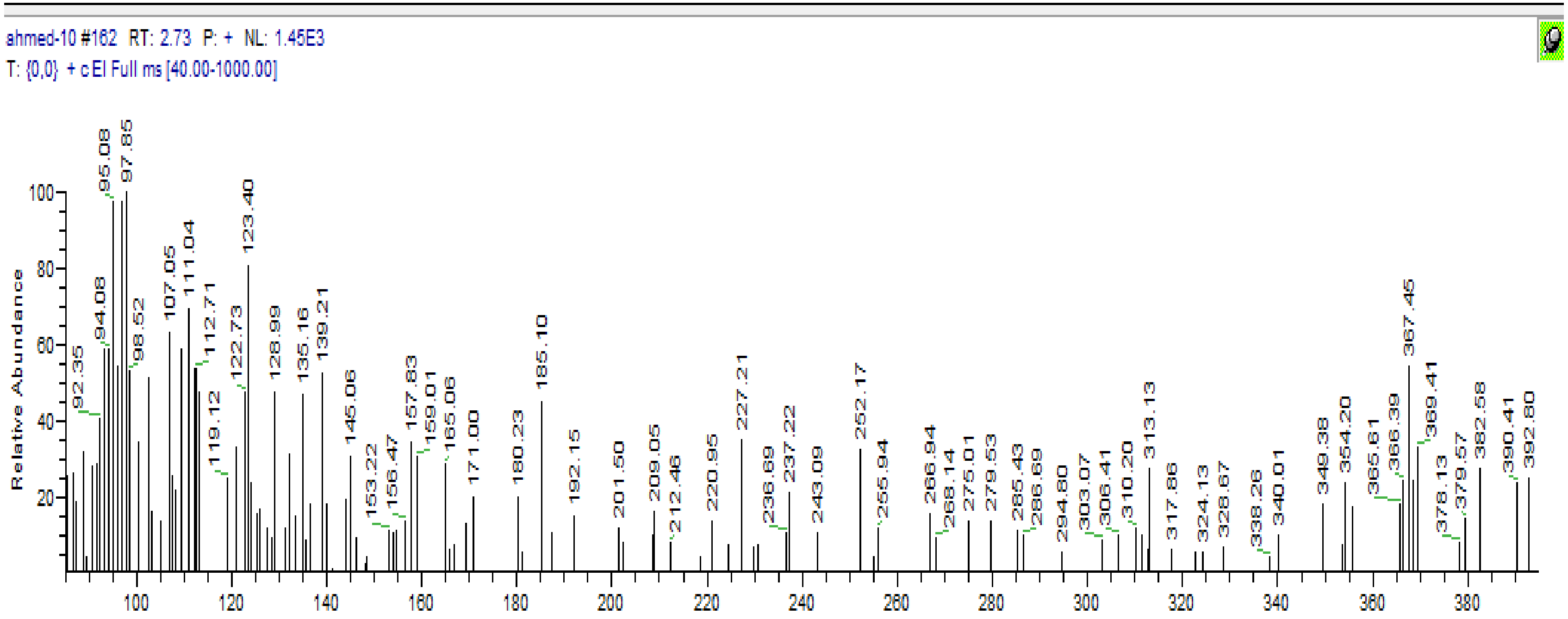

Fig. S46. EI-Mass spectrum of compound **10**

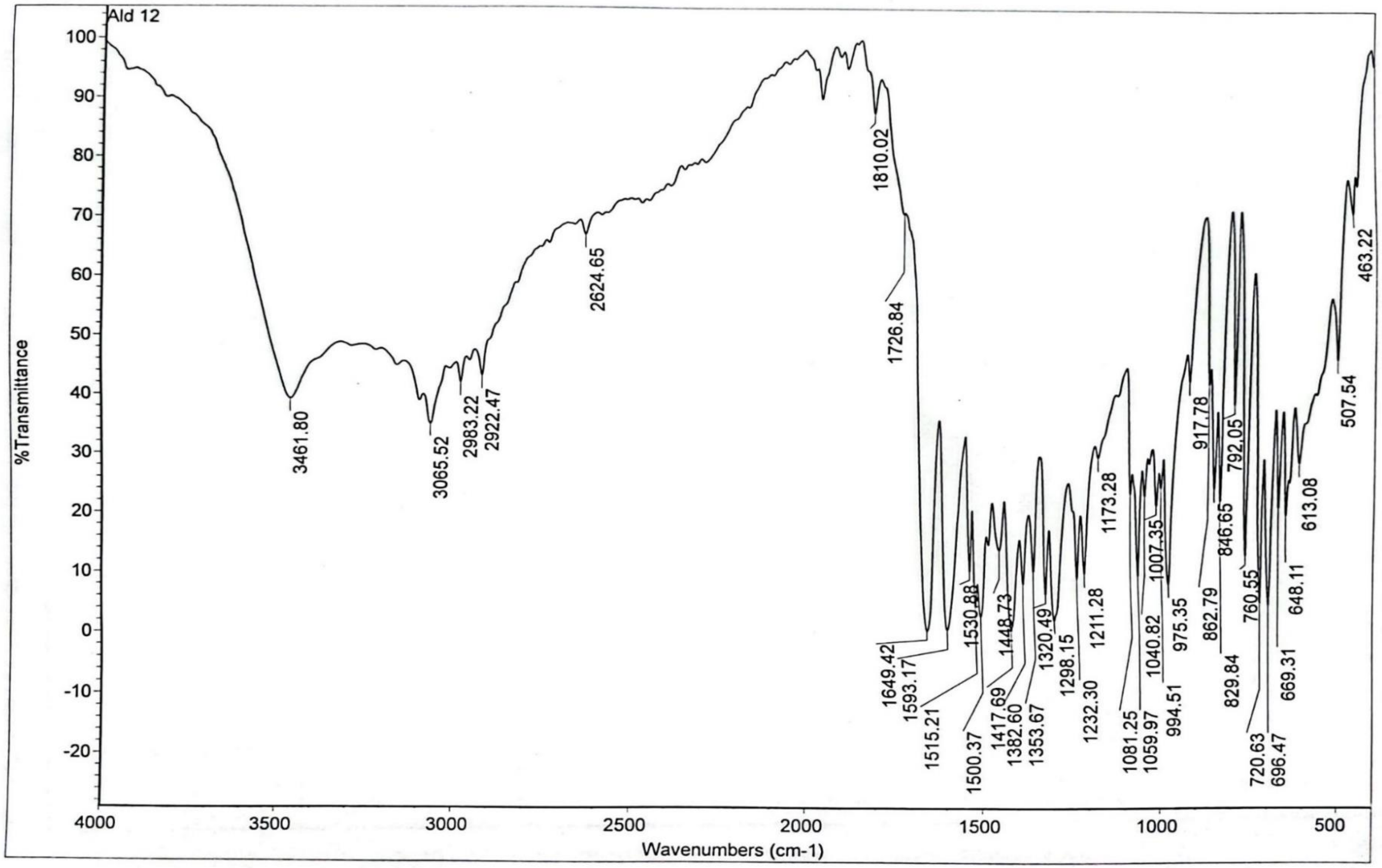

Fig. S47. IR spectrum of compound **11**

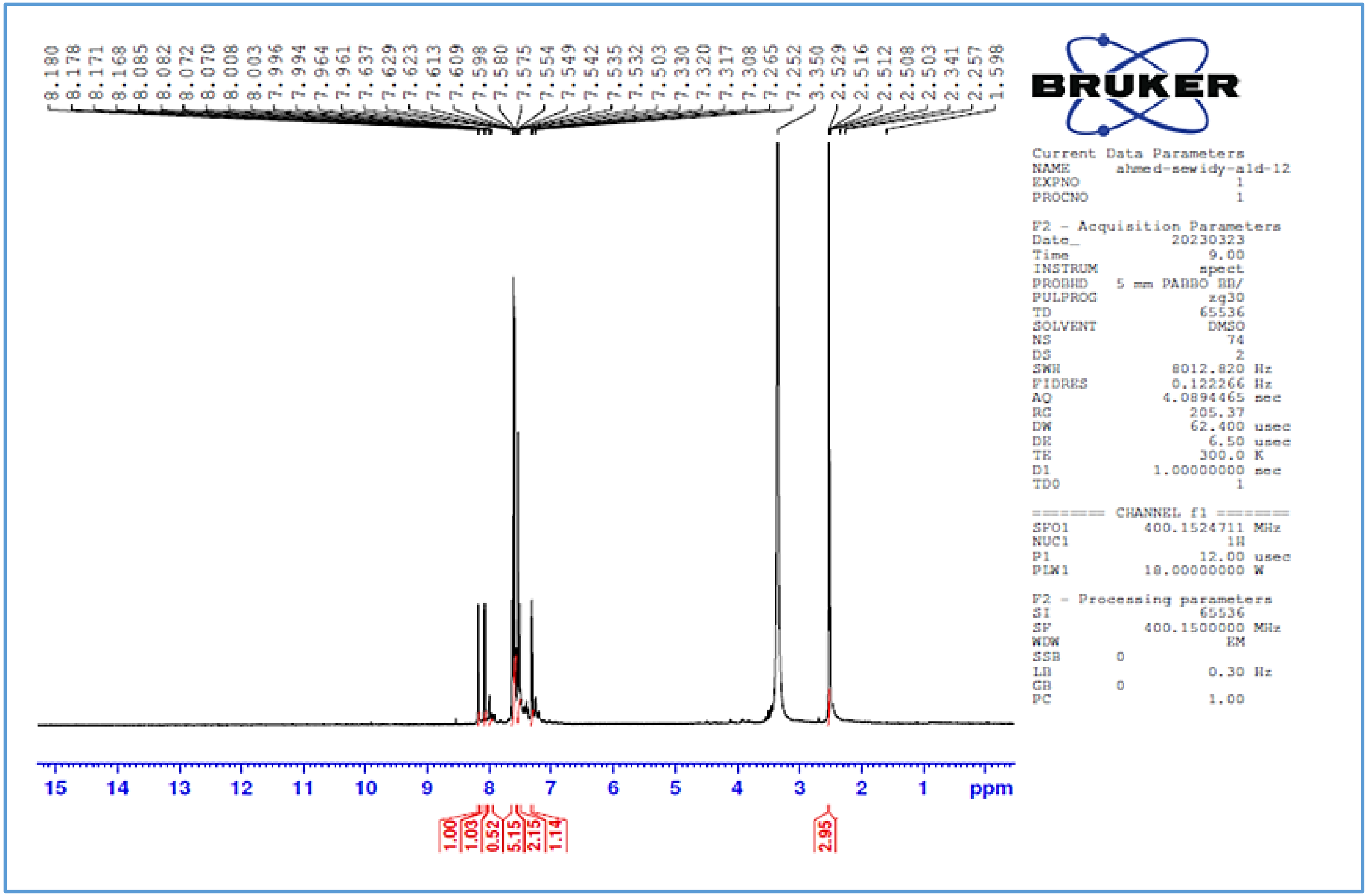

Fig. S48. ^{1}H NMR spectrum (DMSO-d_6) of compound **11**

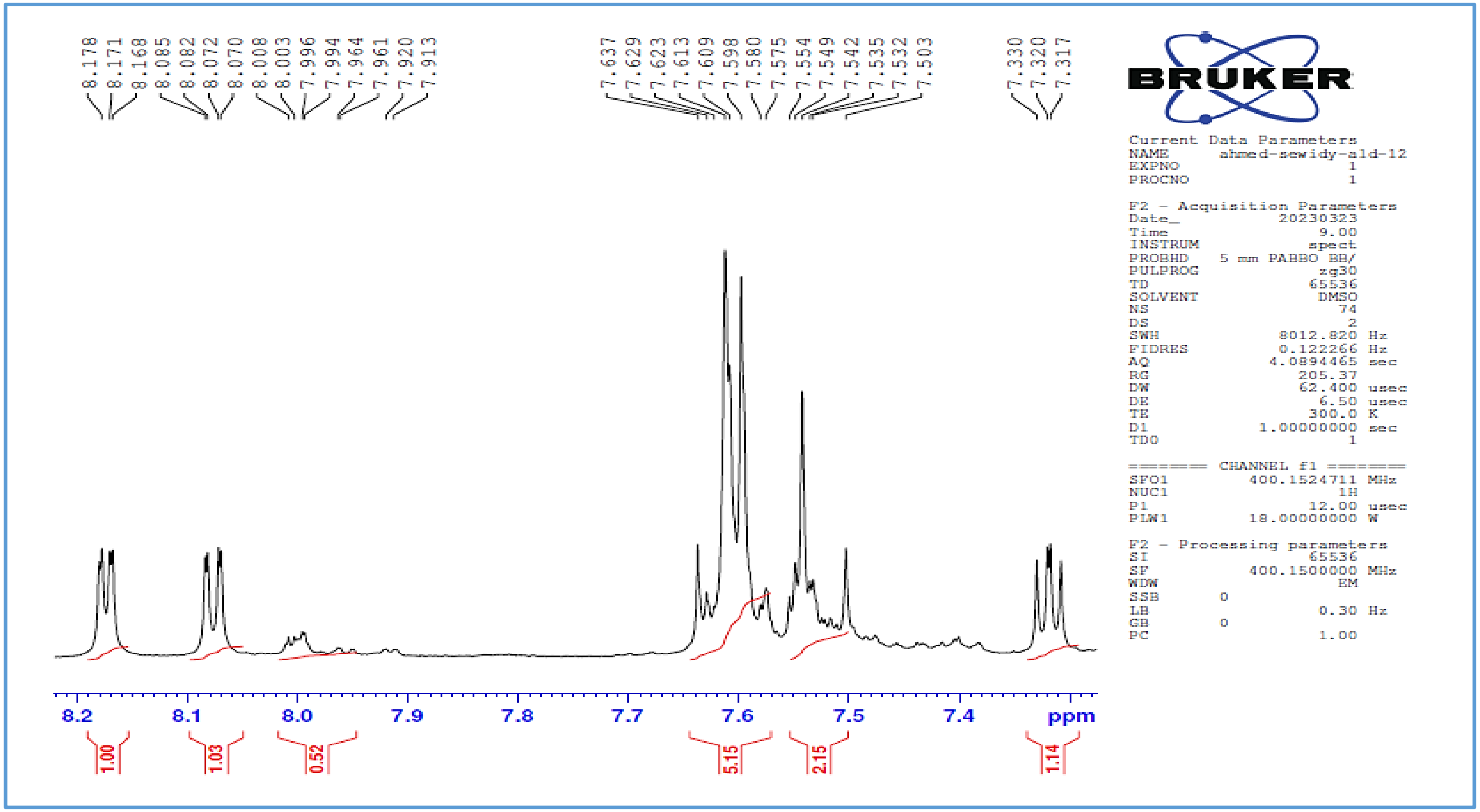

Fig. S49. Cont. ^{1}H NMR spectrum (DMSO-d_6) of compound **11**

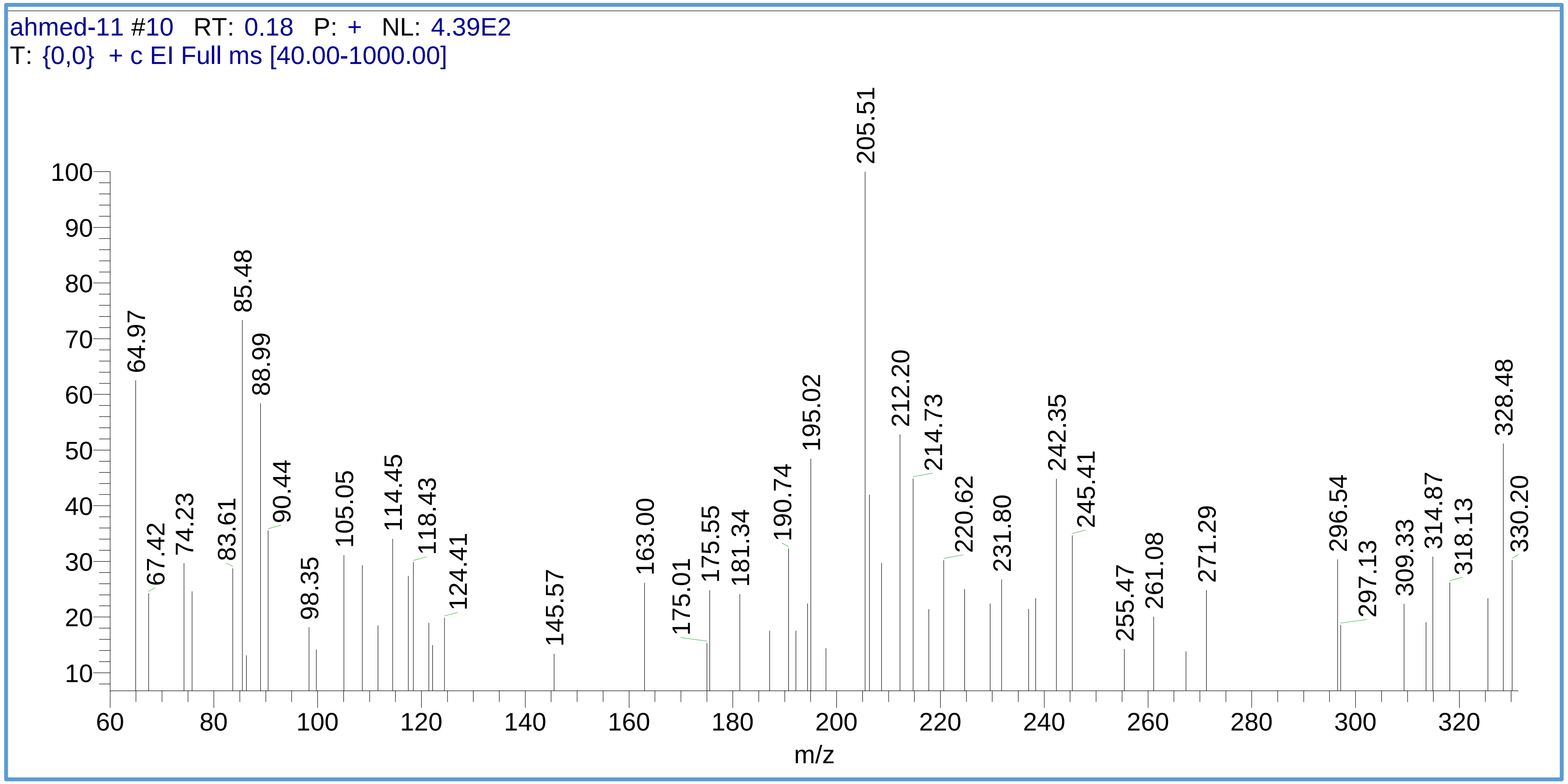

Fig. S50. EI-Mass spectrum of compound **11**
